# Gender Agreement Attraction in Greek Comprehension

**DOI:** 10.3389/fpsyg.2020.00717

**Published:** 2020-04-29

**Authors:** Anastasia Paspali, Theodoros Marinis

**Affiliations:** ^1^Department of English and American Studies, Humboldt University of Berlin, Berlin, Germany; ^2^Department of Linguistics, Universität Konstanz, Konstanz, Germany; ^3^School of Psychology and Clinical Language Sciences, University of Reading, Reading, United Kingdom

**Keywords:** gender attraction, Greek gender agreement, agreement processing, phonological matching, gender violations

## Abstract

This work explores gender agreement attraction in comprehension. Attraction occurs when an agreement error (such as, “the key to the cabinets are rusty”) goes unnoticed, leading to the illusion of grammaticality due to a mismatch between the value of the head and the value of a local intervening phase (attractor). According to retrieval accounts, these errors occur during cue retrieval from memory and predict illusions of grammaticality. Alternatively, representational accounts predict that the errors occur due to the faulty representation of certain features, thus, illusions of ungrammaticality are also expected. In four experiments we explore: (a) whether gender agreement attraction occurs in Greek and the strategy/-ies employed, (b) the role of the agreement target, (c) the timing of gender agreement attraction, (d) the role of phonological matching between the nominal inflectional morphemes of the attractor and the agreement target, and (e) participants’ sensitivity to agreement when there is no conflict from the attractor. In all four experiments, the grammaticality of the sentence and the attractor value (match or mismatch with the head) and also the phonological matching between the attractor and the agreement target in ungrammatical sentences were manipulated. The agreement target was either an adjectival predicate or an object-clitic and the gender value of the head was feminine or neuter. Attraction was found in all measures during the time-course of adjectival predicates (Experiment 1) and object-clitics (Experiment 2), and in timed (Experiment 3), and untimed (Experiment 4) judgments. Even more, both gender values showed attraction and the results mainly suggest that participants experience illusions of grammaticality, confirming retrieval accounts. Phonological matching did not modulate attraction in any of the experiments, suggesting that the similarity in the morphophonological realization between the agreement target and the attractor does not increase attraction. Furthermore, participants were sensitive to gender agreement violations in the absence of gender mismatch between the head and the attractor, suggesting that they respect agreement rules and have both neuter and feminine available in their feature content repertoire, although with some tendency in favor of neuter in feminine agreement contexts. The impact of these findings is discussed within the concept of attraction and sensitivity to agreement violations.

## Introduction

Agreement is one of the core linguistic operations where a head noun and its corresponding agreement target have to accord in their agreement feature specification. Agreement checking can be modulated by interference from a local and syntactically unavailable constituent, a phenomenon which is called *attraction*. Research has shown that the parser is highly accurate in certain structures with highly complex constraints, but less accurate in the implementation of some other simpler constraints (Phillips et al., 2011; Felser et al., 2017). Specifically, in attraction errors, the parser performs less accurately and experiences an “illusion effect,” during which these errors may fleetingly go unnoticed in comprehension. However, the parser appears to be rather selective on the types of interference it is vulnerable to. Thus, studying the cases under which the parser becomes prone to errors can provide psycholinguistic research with knowledge of how users encode and navigate complex linguistic representations in real-time (Phillips et al., 2011, p. 37).

Attraction errors, such as the sentence “The key to the cabinets are unsurprisingly rusty” (Bock and Miller, 1991), may arise due to the presence of a local intervening phrase (“the cabinets”), often called the attractor. Research has shown that people are prone to attraction errors in production, not only in spontaneous speech but also in well-edited texts and academic writing (see Pfau, 2009 for a corpus study), as well as in comprehension. More recently, it has been shown that number in subject-verb agreement is not the only case of attraction and that other features and agreement targets are subject to attraction as well (e.g., Badecker and Kuminiak, 2007; Franck et al., 2008; Slioussar and Malko, 2016).

In this paper, we explore gender agreement attraction at the nominal domain, a less explored case of attraction. Studying attraction errors is of great theoretical importance in the psycholinguistic literature because it is relevant to discussions on the relation between the parser and the grammar and whether they constitute one cognitive system (e.g., grammar constraints are implemented within noisy general memory architecture) or two separate cognitive systems or “snapshots” (see Lewis and Phillips, 2015 and Mancini, 2018 for discussion). We explore gender agreement attraction in Greek comprehension in real-time as well as in timed and untimed judgments in two different agreement targets.

## Background

### Agreement Attraction Accounts

Currently two major groups of accounts have been formulated to explain attraction: representational accounts and retrieval accounts. Representational accounts comprise models which argue that attraction occurs due to faulty or ambiguous representations (e.g., Nicol et al., 1997; Franck et al., 2002; Eberhard et al., 2005; Brehm and Bock, 2013) and can be divided into two distinct categories: models where attraction occurs due to feature movement/percolation (Vigliocco et al., 1995; Eberhard, 1997; Franck et al., 2002) and continuous valuation models (Eberhard et al., 2005) where attraction occurs due to spreading activation. In the percolation model, the marked feature (e.g., plural) of a phrase (attractor, i.e., “to the cabinets”) intervening between the subject and the verb, as in the sentence “The key to the cabinets are rusty,” can erroneously migrate upward to the subject noun phrase during the computation of the subject NP and then can be copied onto the verb. The first studies of agreement attraction in comprehension confirmed this account (e.g., Nicol et al., 1997; Pearlmutter et al., 1999). In the Marking and Morphing model (Eberhard et al., 2005), the hypothesis is that the more plural (conceptually) the subject NP is, the more likely it is for attraction to occur. Attraction takes place at stage two (morphing stage). Similarly to percolation accounts, the Marking and Morphing model predicts that in comprehension, both grammatical and ungrammatical sentences will be affected due to the representation of the complex NP and there is evidence confirming this prediction (e.g., Solomon and Pearlmutter, 2004; Hammerly et al., 2019). At the same time, representational accounts would also expect attraction to occur only when the attractor is morphologically marked, which has also been confirmed in certain studies (Bock and Miller, 1991; Nicol et al., 1997; Staub, 2010; Santesteban et al., 2017).

Alternatively, according to retrieval accounts (e.g., Lewis and Vasishth, 2005; Van Dyke and McElree, 2006), attraction is an error of process of the memory retrieval system, according to which the cues of a certain head should be retrieved on the agreement region and during retrieval the parser might select the wrong NP if there is a partial feature match. Retrieval accounts were initially proposed for comprehension but they were later extended to production. Despite the differences among different retrieval models, what they all seem to have in common is their prediction that interference effects arise when there is a match between multiple encodings in memory and certain cues that should be retrieved. The most well-known retrieval model of attraction is the one proposed by Wagers et al. (2009). The underlying hypothesis is that encountering the agreement target initiates a search through memory to retrieve the head. Misretrieval of the attractor might occur only when an ungrammatical sentence is encountered, such as “the key to the cabinets are rusty,” due to partial cue-matches between the agreement target and the previously identified NPs (e.g., Head noun: NOM, attractor: PL). On the contrary, grammatical sentences are predicted to remain unaffected (but see Cunnings and Sturt, 2018 and Jäger et al., 2017 for inhibitory interference in grammatical sentences with long-distance dependencies) given that the verb is not specified for number and the first NP offers a full match. Certain studies on comprehension have confirmed this hypothesis, mostly for number (e.g., Wagers et al., 2009; Lago et al., 2015, 2018; Slioussar, 2018).

The two groups of accounts make different predictions for attraction. Representational accounts predict attraction to affect both grammatical and ungrammatical sentences, while retrieval accounts predict that attraction affects only ungrammatical sentences. At the same time, the markedness of the attractor can be more easily explained under a representational account.

### Gender Agreement Attraction: Theoretical Considerations and Experimental Evidence

The above accounts have been based largely on number attraction. However, as it has been pointed out in the literature, they should be reconsidered for gender attraction because gender differs from number with respect to the involvement or non-involvement of meaning (Bock, 2004, p. 116): the gender of a noun in grammatical-gender languages can be either conceptual or grammatical, whereas number on most nouns has semantic motivation. Eberhard et al. (2005, p. 553) discuss the extent to which their Marking and Morphing model could account for gender attraction and they are open to the idea that this model is able to account for gender attraction when the noun bears a conceptual gender. When it is not, the Marking stage is cancelled (see also Acuña-Fariña et al., 2014; Slioussar and Malko, 2016). However, Eberhard et al. (2005) also argue that the model might be relevant to grammatical gender, although this relevance is restricted to the lexical level (p. 553-4). Taking the latter under consideration, it seems that they do not necessarily exclude grammatical gender from their model, although it is not clear how exactly gender agreement attraction would be explained.

Other researchers suggest alternative accounts for agreement attraction with grammatical gender. Slioussar and Malko (2016) point out that (number and/or gender) attraction is still possible without any semantic effects, and consequently, attraction should stem from a different process (e.g., from the formal properties of features). In a similar vein, Acuña-Fariña et al. (2014) claim for grammatical gender that only competing percolation (of form) inside a complex NP could explain gender attraction. They further argue that in percolation/copying models there are many reasons why the agreement mechanism could fail, such as it might simply select the gender feature of the wrong noun in the complex NP to pass to the predicate. Thus, percolation accounts could predict gender attraction even with inanimate nouns which hold arbitrary gender where a copying mechanism would apply lexically. Percolation would occur at the level of form, reflecting that the feature of the wrong NP has been selected. Thus, both grammatical and ungrammatical sentences are expected to be influenced by gender attraction and also the marked attractor is expected to cause (more) attraction.

On the other hand, retrieval accounts could apply for gender agreement attraction in both production and comprehension (e.g., Badecker and Kuminiak, 2007; Wagers et al., 2009) irrespective of the conceptual and grammatical gender distinction. Under this group of models, gender attraction is expected to influence ungrammatical sentences only, similarly to number, while both marked and unmarked attractors are expected to show attraction.

Most studies on gender attraction have focused on production and have reported the existence of the phenomenon in languages with a bipartite or tripartite gender distinction. These studies revealed that marked attractors do not significantly cause more attraction in a bipartite system (e.g., Vigliocco and Franck, 1999 for French and Italian). However, marked attractors seem to influence languages with a tripartite system, such as Slovak (Badecker and Kuminiak, 2007) and Russian (Slioussar and Malko, 2016, Experiment 1).

In comprehension, attraction studies are scarce. For example, there are currently only two studies testing gender attraction in a language with a tripartite gender distinction (Slioussar and Malko, 2016 for Russian; Tucker et al., 2016 for Arabic). Slioussar and Malko (2016) found no markedness effects in Russian comprehension and no attraction in grammatical sentences, confirming retrieval accounts of agreement attraction. In Arabic, Tucker et al. (2016) found markedness effects; however, attraction affected ungrammatical sentences only. The authors conclude that their results seem to be better explained under retrieval accounts of agreement attraction and that the effect of markedness may be more relevant to agreement itself.

There are also six attraction studies in Spanish that consistently found no markedness effects (Martin et al., 2012, 2014; Acuña-Fariña et al., 2014) or no attraction at all (Fuchs et al., 2015). However, results are mixed with respect to whether attraction influences grammatical sentences (e.g., Martin et al., 2012, 2014; Acuña-Fariña et al., 2014) or not (e.g., Cunnings et al., 2017). An important aspect that needs to be taken into consideration, though, is the fact that the structures and/or the factor of animacy of the head/attractor differed across these studies. Additionally, most studies have only measured the time-course of attraction. Only Fuchs et al. (2015) and Cunnings et al. (2017) (Experiment 1) measured gender attraction in untimed acceptability judgments and they failed to find attraction, reflecting that (gender) attraction may be dependent on timing. One methodology that has not been used with gender attraction so far is speeded judgments which are considered to be more sensitive to representational effects (Wagers et al., 2009). For French and Italian, Villata et al. (2018) and Villata and Franck (2019) found that grammatical sentences are affected by marked (feminine) attractors and they attribute their findings to predictive processing as well as retrieval.

Another important aspect which influences attraction is the type of attractor. It has been shown that certain manipulations on the attractor play an important role. One of those is the morphophonological similarity between the attractor and the head (Hartsuiker et al., 2003; Slioussar, 2018). However, some other manipulations of the attractor do not influence attraction in comprehension (e.g., notional number and plausibility of the attractor, Schlueter et al., 2018). To our knowledge, such attractor manipulations have only been scarcely explored for gender agreement attraction in comprehension (e.g., Fuchs et al., 2015). In our study, we do so by focusing on another morphophonological manipulation, this time between the attractor and the agreement target. We namely explore whether *phonological matching* (Agathopoulou et al., 2010) between the nominal inflectional morphemes of the attractor and the agreement target influences gender attraction; i.e., the agreement target not only shares the same gender value with the attractor but also the exact same morphophonological realization for this gender value, as in example (2). Agreement attraction occurs during certain syntactic computations (e.g., at the feature-checking stage). Thus, whether attraction is influenced by certain morphophonological encodings provides us with a window into the modularity vs. interactivity of language (see Franck et al., 2008 for discussion).

Furthermore, recent studies explore the role of agreement target itself in attraction. This body of research (e.g., Phillips et al., 2011; Dillon et al., 2013) has shown that agreement targets are not all equally vulnerable to attraction (e.g., reflexive pronouns are less prone to attraction due to the application of syntactic rather than morphological cues during retrieval). In the present study we test gender agreement attraction not only with adjectival predicates but also with object-clitics targets. Our main motivation is to better understand which agreement targets are vulnerable to gender attraction. To that end, we explore whether across all different measures employed in this study, clitic dependencies are prone to gender attraction effects similarly to those found for verbs and adjectival predication. To date, only one study has tested object-clitics in sentence comprehension and found number attraction (Santesteban et al., 2017).

Apart from attraction, a large part of the psycholinguistic literature has also focused on studying simple agreement violation paradigms. This body of research (e.g., Fuchs et al., 2015; Bañón and Rothman, 2016; Kaltsa et al., 2016) focused on the sensitivity to agreement rules during real-time processing and provided useful evidence regarding the structural organization of feature values within a certain language (see Fuchs et al., 2015 for discussion).

### Gender Agreement in Greek

Greek is a language with grammatical gender. At the same time, phonological criteria seem to partially predict gender values (Alexiadou, 2004; Alexiadou et al., 2007), while declension classes do not provide a safe criterion on the categorization of nouns into different gender values. Greek bears a three-gender distinction of masculine, feminine, and neuter with various agreement targets, such as determiners, attributive and predicate adjectives, adjectival participles, personal, demonstrative, definite, indefinite, interrogative, relative pronouns, and inflected numerals. However, there is no one-to-one match between a certain suffix and a gender value (see *dromos, harakas, fortistis* in (1a), many suffixes are distributed across (all or two) values on nouns (see *dromos, isodos, edafos* in (1a) as well as agreement targets, such as attributive adjectives (1b), adjectival predicates (1c), and object-clitic pronouns (1d). Additionally, number and case are marked on the same suffix with gender in both head nouns and targets (e.g., Anastasiadi-Simeonidi and Chila-Markopoulou, 2003; Ralli, 2003). Interestingly, the features of these suffixes can also interact with each other within the inflectional system leading to morphophonological ambiguities/syncretism (1b).

**Figure:**
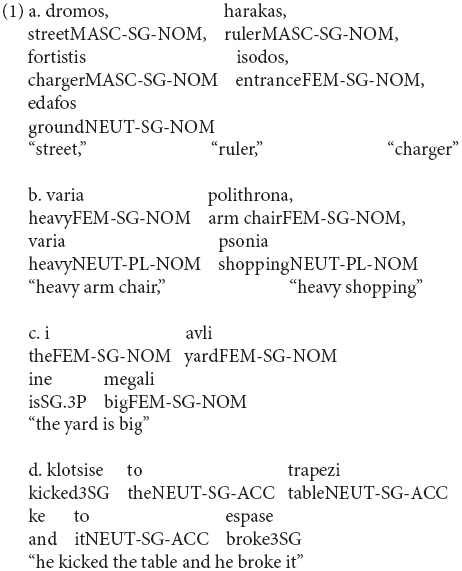


Another issue which is relevant to the present work is gender markedness in Greek. Theoretical accounts (e.g., Anagnostopoulou, 2017) as well as experimental data (e.g., Kazana, 2011) show that the default gender which holds unmarked properties in Greek is mediated by animacy; neuter is the default gender in inanimate nouns and masculine in animate nouns. Focusing on neuter, the majority of nouns and neologisms are neuter in Greek (Anastasiadi-Simeonidi and Chila-Markopoulou, 2003). Neuter is also the index of metalinguistic use as well as the value of impersonal structures and it also modifies genderless elements, such as sentences, prepositions, and “mentioned” words. Greek children overgeneralize neuter with inanimate nouns when errors occur, especially with feminine nouns (see Tsimpli and Hulk, 2013 and references therein). Neuter is the most ambiguous value, bearing the same marking on the determiner and the noun itself for NOM and ACC (*to kastro* “the castle”) while determiners disambiguate case in feminine nouns (*i limni* vs. *ti limni*, “the lake”). In cases of subject co-ordination with two inanimate nouns, feminine agreement occurs when the nouns are both feminine (but neuter may still be considered an option by some Greek speakers even in this case, see Kazana, 2011, p.79), while neuter agreement is the elsewhere condition when there is conflict between the gender values of the two inanimate nouns (Kazana, 2011; Anagnostopoulou, 2017; Manasis, 2019).

## The Present Research

The present research explores gender agreement attraction in Greek sentence comprehension in real-time (Study 1) as well as in timed and untimed acceptability judgments (Study 2) across two structurally different configurations, i.e., adjectival predicates and object-clitics. Thus, we investigate not only how participants process sentences in real-time but also how they judge the acceptability of sentences with or without time pressure. This is the first study to investigate gender attraction across two structurally different configurations by using similar methods and materials, and the first attraction study in Greek. The current experiments address the following questions:

(a) Does Greek show attraction during real-time sentence comprehension? (Experiment 1)
(b) Are object-clitics prone to gender agreement attraction? (Experiment 2)
(c) Does gender agreement attraction occur under time pressure, i.e., in timed (speeded) grammaticality judgments? (Experiment 3)
(d) Is gender attraction evident in the absence of time pressure, i.e., in untimed acceptability judgments? (Experiment 4).

Across all four experiments, the same topics are explored, that is the processing strategies the parser aligns with during the comprehension of agreement attraction: namely, the existence of attraction in grammatical vs. ungrammatical sentences, the markedness of the attractor, phonological matching, and the sensitivity to gender agreement rules in the absence of attraction.

## Study 1

Study 1 consisted of Experiment 1 and Experiment 2.

### Experiment 1

Experiment 1 investigated gender attraction in adjectival predicates.

#### Methodology

##### Participants

Fifty-two healthy adult native speakers of Greek (mean age = 23.2, range = 18–33, 23 females) completed Experiment 1. They were born and raised in Greece, they had never lived abroad, and both of their parents were Greek. In this and all other experiments reported, all participants provided informed consent and they received a fee of 10 Euros/hour for their participation. The participants were recruited via mailing lists, and they were tested individually in a quiet office of the Aristotle University of Thessaloniki in Greece.

##### Materials and Design

Sentences including a set of feminine and a set of neuter head nouns with their agreement targets were tested; the following variables were manipulated for each set in a 2 × 2 within-subjects design: (a) the Grammaticality of the sentence (*grammatical, ungrammatical*), depending on the gender value of the predicate; and (b) the Attractor (*match, mismatch*), depending on whether the gender value of the attractor matches or mismatches the value of the head. Table 1 shows a complete item for feminine and neuter heads.

**TABLE 1:** A complete item for feminine and neuter heads in Experiment 1. R stands for different regions.

R1-2 *Introduction*	´Oταν o $\Gamma \iota \overset{´}{\alpha }\nu \nu \eta \varsigma$$\mu \pi \overset{´}{\eta }\kappa \varepsilon$ στην κo$\upsilon \zeta \overset{´}{\iota }\nu \alpha$, $\beta \rho \overset{´}{\eta }\kappa \varepsilon \ldots$ When the John went in-the kitchen, was looking for…		
	
*Feminine heads*	R3 - HEAD	R4 - ATTRACTOR	R5 - TARGET
grammatical match	τη $\sigma \upsilon \nu \tau \alpha \gamma \overset{´}{\eta }$ the_(FEM)_ recipe_(FEM)_	γιατηνπ$\overset{´}{\iota }$τσα for the_(FEM)_ pizza_(FEM)_	σκισμ$\overset{´}{\varepsilon }$νη torn_(FEM)_
ungrammatical match	τησυνταγ$\overset{´}{\eta }$ the_(FEM)_ recipe_(FEM)_	γιατηνπ$\overset{´}{\iota }$τσα for the_(FEM)_ pizza_(FEM)_	σκισμ$\overset{´}{\varepsilon }$νo torn_(NEUT)_
grammatical mismatch	τησυνταγ$\overset{´}{\eta }$ the_(FEM)_ recipe_(FEM)_	γιατo ψωμ$\overset{´}{\iota }$ for the_(NEUT)_ bread_(NEUT)_	σκισμ$\overset{´}{\varepsilon }$νη torn_(FEM)_
ungrammatical mismatch	τησυνταγ$\overset{´}{\eta }$ the_(FEM)_ recipe_(FEM)_	γιατo ψωμ$\overset{´}{\iota }$ for the_(NEUT)_ bread_(NEUT)_	σκισμ$\overset{´}{\varepsilon }$νo torn_(NEUT)_
R6-8 Continuation	π$\overset{´}{\alpha }$νωστo τραπ$\overset{´}{\varepsilon }$ζιτησκoυζ$\overset{´}{\iota }$νασ.		
	on the table of the kitchen.		
“When John went into the kitchen, he found the recipe for the pizza/bread torn on the kitchen table.”			

**R1-2 *Introduction***	**´Oταν o Γι$\overset{´}{\alpha }$ννησμπ$\overset{´}{\eta }$κεστηνκoυζ$\overset{´}{\iota }$να, βρ$\overset{´}{\eta }$κε…** **When the John went in-the kitchen, was looking for…**		
	
***Neuter heads***	**R3 - HEAD**	**R4 - ATTRACTOR**	**R5 - TARGET**

grammatical match	τo κoυτ$\overset{´}{\alpha }$λι the_(NEUT)_ spoon_(NEUT)_ spoon_(NEUT)_	γιατo γλυκó for the_(NEUT)_ dessert_(NEUT)_	λερωμ$\overset{´}{\varepsilon }$νo stained_(NEUT)_
ungrammatical match	τo κoυτ$\overset{´}{\alpha }$λι the_(NEUT)_ spoon_(NEUT)_	γιατo γλυκó for the_(NEUT)_ dessert_(NEUT)_	λερωμ$\overset{´}{\varepsilon }$νη stained_(FEM)_
grammatical mismatch	τo κoυτ$\overset{´}{\alpha }$λι the_(NEUT)_ spoon_(NEUT)_ spoon_(NEUT)_	γιατησo$\overset{´}{\upsilon }$πα for the_(FEM)_ soup_(FEM)_	λερωμ$\overset{´}{\varepsilon }$νo stained_(NEUT)_
ungrammatical mismatch	τo κoυτ$\overset{´}{\alpha }$λι the_(NEUT)_ spoon_(NEUT)_ spoon_(NEUT)_	γιατησo$\overset{´}{\upsilon }$πα for the_(FEM)_ soup_(FEM)_	λερωμ$\overset{´}{\varepsilon }$νη stained_(FEM)_
R6-8 Continuation	π$\overset{´}{\alpha }$νωστo τραπ$\overset{´}{\varepsilon }$ζιτησκoυζ$\overset{´}{\iota }$νασ.		
	on the table of the kitchen.		
“When John went into the kitchen, he found the spoon for the dessert/soup stained on the kitchen table.”			

Twenty-four items (6 per condition, 24 for each gender value) were distributed across four lists in a Latin Square design after being combined with 48 grammatical fillers, which appeared in pseudo-random order. Consequently, 25% of the trials within each list were ungrammatical. Additionally, eight grammatical practice trials were also created and none of them included the target configuration. Feminine and neuter heads were chosen to test for markedness asymmetries in inanimate nouns between the most marked value (feminine) and the least marked one (neuter), see section “Gender agreement attraction: theoretical considerations and experimental evidence.” Sentences of the type *Introduction - Verb - Head - Attractor - Agreement target - Adverb - PP - Modifier* were constructed, where the Head is a DP in ACC, the Attractor is an intervening PP including an NP in ACC licensed by the preposition *ja* (“for”) and it is syntactically unavailable for agreement, and Agreement target is a past-participle. The structure of a small clause was selected because a configuration in which a noun intervenes between the head noun and the agreement target and in which all the elements are inflected for gender was needed. This configuration with the head noun in the object position - instead of the subject position - was selected because Greek bears morphological case, which could decrease attraction due to morphological disambiguation between ACC (i.e., attractor) and NOM (i.e., head) (Hartsuiker et al., 2003; Slioussar, 2018). Additionally, the ja-PP was intentionally selected because it is less ambiguous compared to other PPs with respect to its attachment point (Katsika, 2009). As for the suffixes of the heads, feminine heads ended in -*a* and -*i*, and neuter heads ended in -*o* and -*i* and -*ma*. Thus, both sets of heads included at least one suffix with a strong phonological predictive value (predictive values: feminine -*a* = 0.97, feminine -*i* = 0.45, neuter-*o* = 98, neuter-*i* = 0.55, neuter-*ma* = 0.75, Mastropavlou, 2006, p.134). Half of the suffixes of the attractors were creating a phonological match with the suffix of the agreement target in the ungrammatical conditions to test the role of phonological realization (Phonological matching: *matching, mismatching*) on attraction (2), i.e., whether phonological matching exhibits more attraction.

**Figure:**
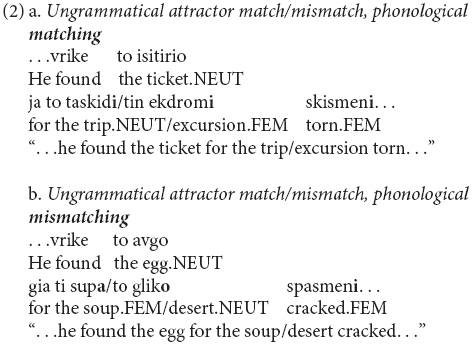


There were three verbs in past-tense and perfective aspect, equally distributed across items, and they consisted of two-syllables: βρ*$\overset{´}{\eta }$*κε “found,” ε*$\overset{´}{\iota }$*χε “had” and ε*$\overset{´}{\iota }$*δε “saw,” The agreement target was followed by certain prepositions which were also used an equal number of times. Frequency, length (in syllables), and plausibility were also taken under consideration. The length was between 2-4 syllables for heads, 2-3 syllables for attractors, 3-4 syllables for agreement targets, and 2 syllables for the adverbs following the target. Given that different nouns for attractors between the matching (attractor match) and mismatching (attractor mismatch) conditions were used, frequency was also measured by extracting and comparing the lemma frequency from the National Hellenic Corpus. The difference in frequency between the matching and mismatching attractors was not significant (Supplementary Material S1). Additionally, careful consideration was taken to normalize the items with respect to plausibility of head noun - attractor - agreement target between the match and mismatch conditions. This was done by receiving feedback for the naturalness of the items from both linguists as well as naïve native speakers.

All trials were followed by a yes or no comprehension question (with theta-role reversal or lexical replacement) and were never referring to the head, the attractor, or the agreement target. Grammatical sentences were recorded in a sound booth by a female native speaker of Greek in such a way to avoid co-articulation between the words that belonged to different segments. The comprehension questions were recorded by a male native speaker of Greek. The ungrammatical versions of the sentences were created by splicing out the agreement target and replacing it with the target from a grammatical sentence (Ferreira et al., 1996; Marinis, 2010; Papadopoulou et al., 2014).

#### Procedure

In Experiment 1, the self-paced listening methodology was employed (Ferreira et al., 1996; Marinis, 2010) and reaction-time and accuracy data were collected. In this and all other experiments of the present work, the presentation of the stimuli and the recording of end-of-sentence responses and/or reaction times across regions were controlled by the E-prime software (Psychology Software Tools Inc. [E-Prime 3.0], 2016 Pittsburgh, PA, United States). The stimuli were auditorily presented. We chose the auditory modality because we were interested in exploring the influence of attraction in auditory comprehension, which is underexplored and forms a more unplanned and spontaneous modality in daily communication compared to the visual (reading) one. Both the auditory method of Experiments 1 and 2 (see section “Experiment2”) as well as of Experiment 4 (see section “Experiment 4”) have been used extensively and successfully in the previous literature (see Fuchs et al., 2015 and also Papadopoulou et al., 2014 for discussion) and are also highly important for research in young children and heritage speakers (Paspali, 2019a, b) and/or for languages without a writing system. Also note, that the reading method of Experiment 3 (see section “Experiment 3”) showed similar results with the other two methods.

The sentences were divided in eight regions as indicated in the example (3) below; R3 shows the Region of the Head, R4 the Region of the Attractor, and R5 the Region of the agreement target. The duration of the sound files did not differ on the Attractor between the match and the mismatch condition and the same applies to the duration of the agreement target (Region 5) between the grammatical and the ungrammatical agreement target (Supplementary Materials S2.1.1, S2.2.1).

**Figure:**
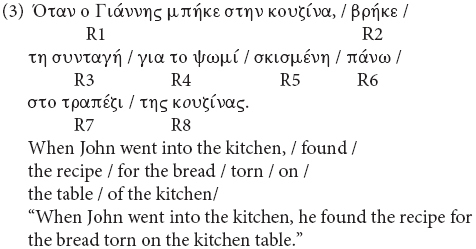


Participants were tested individually. They were seated comfortably at a desk and they listened to the sentences on a laptop via headphones. In each trial, they listened to a sentence region by region by pressing a button on a button box. Participants were instructed to listen to each region for comprehension and to press the button as soon as they were ready to listen to the next region. To proceed to the comprehension question, participants had to press the same button and then had to press the yes/no button to respond to the comprehension question. They received feedback on their accuracy in each trial; the LED lights on the button box flashed green three times for a correctly answered question and red for an incorrectly answered question. The underlying rationale of the task is that longer RTs for a certain condition on a specific region indicate relatively higher processing difficulty compared to a control condition. The experiment lasted approximately 40 min.

#### Predictions

If gender attraction occurs, a *facilitation* in RTs in the ungrammatical mismatch condition is expected on the region (R5) of the agreement target (or the spill-over regions) compared to the ungrammatical match condition. However, if grammatical sentences are also affected, an *inhibition* in the grammatical mismatch condition compared to the grammatical match condition is also expected with longer RTs for the Attractor region (R4) due to the representation of the complex NP itself (or the downstream regions), indicating processing difficulties due to mismatch^1^. Additionally, attraction is expected to be (more) evident with marked attractors (neuter heads with feminine attractors) than with unmarked attractors (feminine heads with neuter attractors) if markedness of the attractor plays a role. Moreover, if participants exhibit sensitivity to gender agreement violations in real-time, a main effect of Grammaticality is expected on the agreement region and/or the two post-critical regions (longer RTs in the ungrammatical match condition compared to the grammatical match one). Finally, if phonological matching plays a role in regulating attraction, an interaction between Attractor and Phonological matching is expected with shorter RTs in sentences with phonological matching compared to sentences without phonological mismatching.

#### Analysis

A 70% threshold of accuracy was set for participants in the filler sentences and for items in the experimental sentences. Thus, two items were removed in Experiment 1, and the data from one participant and three items in Experiment 2 (one from the dataset of the feminine heads, two from the dataset of the neuter heads). For the RT analysis, only trials in which participants had correctly responded to the comprehension question remained in the dataset. RTs were calculated by subtracting the duration of each region (sound file) from the raw RTs for each item to obtain the Difference Time (Ferreira et al., 1996, p. 327; Marinis and Saddy, 2013, p. 169; Papadopoulou et al., 2014, p. 60). Extreme values below 150 ms and above 2,500 ms were excluded from the analysis based on previous literature (Marinis and Saddy, 2013; Parker et al., 2015; Ratcliff, 1993; Schlueter et al., 2018). Additionally, values below or above 2.5 SD by condition were treated as outliers and deleted (Häussler, 2009; Franck et al., 2015; Ahn and Jiang, 2018). Overall, the data cleaning did not affect more than 15% of the data (Ratcliff, 1993) in Experiment 1 (Supplementary Material S3). The regions analyzed were R3 (the region of the Head/pre-critical region), R4 (the region of the Attractor/first critical region), R5 (the region of the agreement target/second critical region), and the post-critical regions, namely R6 and R7 (for potential spillover effects), similar to previous attraction studies (e.g., Wagers et al., 2009; Lago et al., 2015; Slioussar and Malko, 2016; Parker and An, 2018). RTs were log-transformed in all models.

The data analysis for this and all other experiments was conducted in R (R Development Core Team, 2017) in the library languageR with the lme4 package. For accuracy data, logistic mixed models were fit, and in the case of the log-transformed Reaction Times (RTs) linear mixed-effects models were fit. Grammaticality and Attractor were modeled as fixed effects, using effects coding with orthogonal contrasts (Grammaticality: *grammatical* = −0.5, *ungrammatical* = 0.5, Attractor: *match* = −0.5, *mismatch* = 0.5), as well as their interaction. Following Dillon et al. (2013) and Parker and An (2018), additional linear mixed-effects models were fit to focus on the effect of attraction (i.e., the amount of facilitation for ungrammatical sentences with attractor mismatch relative to ungrammatical sentences with attractor match) represented as “Attraction model” in Tables 2, 4. Effects coding was also used here for Attractor (*match* = −0.5 *mismatch* = 0.5). For comparison, analyses on the raw untrimmed data have also been conducted (Supplementary Material S8), given that certain studies have shown that data transformations may obscure attraction effects (e.g., Staub, 2010; Tucker and Almeida, 2017), and both analyses show similar results. To test whether phonological matching influences attraction in ungrammatical sentences, a model with Phonological matching (*phonological matching* = −0.5, *phonological mismatching* = 0.5) and Attractor (*match* = −0.5, *mismatch* = 0.5) as fixed effects as well as their interaction was also fit for ungrammatical sentences. Furthermore, all pairwise comparisons reported in this manuscript were conducted using similar contrasts (effects coding) and models (lmer/glmer) as the corresponding omnibus analysis. The initial random effects structure of all models included random intercepts and slopes for both participants and items. When the models failed to converge, the maximal random effects structure was gradually simplified following the recent literature (Barr et al., 2013) until convergence was reached.

**TABLE 2:** Linear mixed-effects model results in Experiment 1with feminine and neuter heads.

Feminine Heads								
	Region 4 (Attractor)				Region 5 (Agreement target)			
	β	SE	t	p	β	SE	t	p
Grammaticality	0.010	0.027	0.37	0.712	0.063	0.036	1.75	0.094
Attractor	–0.033	0.035	–0.95	0.351	–0.079	0.026	–3.06	0.003
Grammaticality:Attractor	–0.001	0.077	–0.02	0.985	–0.121	0.047	–2.60	0.010
*Attraction model*	–0.036	0.045	–0.81	0.430	–0.144	0.035	–4.14	<0.001

	**Region 6 (post-critical 1)**				**Region 7 (post-critical 2)**			
	**β**	**SE**	**t**	**p**	**β**	**SE**	**t**	**p**

Grammaticality	0.081	0.034	2.40	0.025	0.037	0.038	1.00	0.330
Attractor	–0.044	0.035	–1.27	0.217	–0.073	0.037	–1.99	0.058
Grammaticality:Attractor	–0.003	0.076	–0.04	0.970	–0.074	0.084	–0.88	0.387
*Attraction model*	–0.049	0.030	–1.67	0.097	–0.115	0.055	–2.10	0.047

**Neuter heads**								

	**Region 4 (Attractor)**				**Region 5 (Agreement target)**			
	**β**	**SE**	**t**	**p**	**β**	**SE**	**t**	**p**

Grammaticality	–0.052	0.032	–1.7	0.112	0.014	0.045	0.30	0.765
Attractor	0.038	0.036	1.07	0.297	0.051	0.036	1.41	0.173
Grammaticality:Attractor	0.211	0.076	2.77	0.011	0.186	0.078	2.38	0.027
*Attraction model*	0.143	0.060	2.37	0.028	0.139	0.035	4.04	<0.001

	**Region 6 (post-critical 1)**				**Region 7 (post-critical 2)**			
	**β**	**SE**	**t**	**p**	**β**	**SE**	**t**	**p**

Grammaticality	0.107	0.036	2.99	0.007	0.041	0.043	0.96	0.347
Attractor	0.039	0.035	1.12	0.277	0.089	0.029	3.03	0.006
Grammaticality:Attractor	0.070	0.047	1.49	0.141	0.072	0.061	1.19	0.240
*Attraction model*	0.078	0.064	1.22	0.237	0.122	0.040	3.05	0.002

#### Results

**FIGURE 1:**
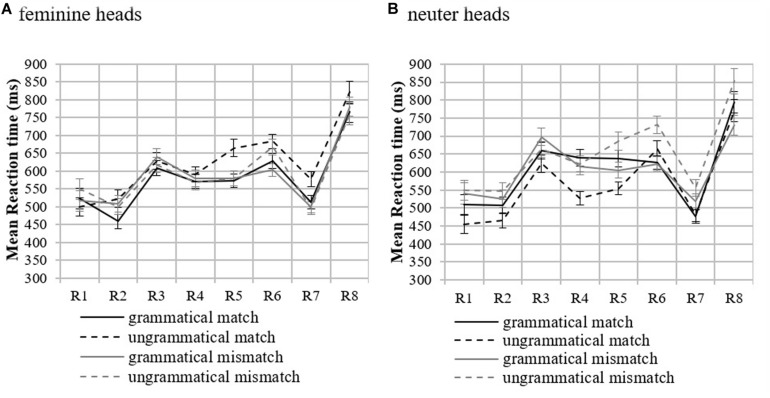
Mean reaction times by condition in feminine **(A)** and neuter **(B)** heads in Experiment 1. Error bars indicate the standard error of the mean across participants. R on the *x*-axis stands for Region. *Region 3*: Head, *Region 4*: Attractor, *Region 5*: Agreement target (adjectival predicate), *Region 6*: first post-critical region, *Region 7*: second post-critical region, and *Region 8*: sentence-final region.

No significant differences by condition were found in accuracy to comprehension questions (Supplementary Material S6). Thus, we proceeded with the RT analyses. Figure 1 shows participants’ mean RTs and the standard error of the mean by condition and region in both feminine and neuter heads (see Supplementary Material S4 for table with the means). Table 2 reports the results of the mixed-effects models in the log transformed RTs for Regions 4–7.

##### Feminine heads

**FIGURE 2:**
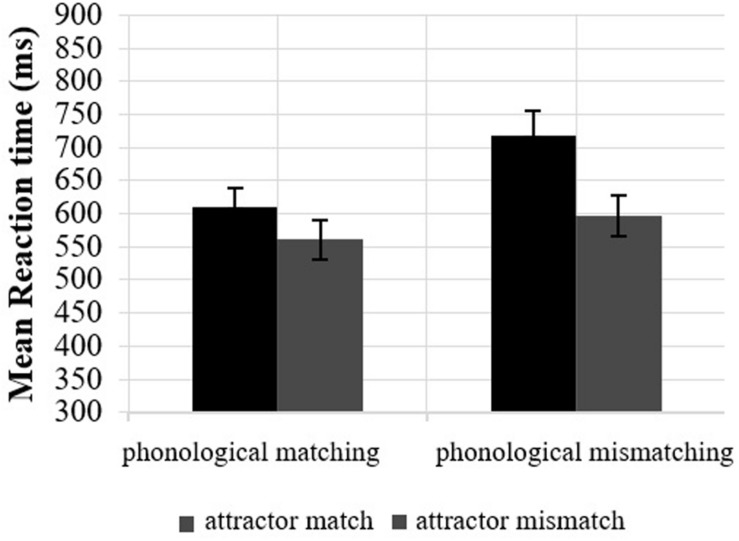
Mean reaction times in ungrammatical sentences by *Attractor* (match, mismatch) and *Phonological matching* (phonological matching/mismatching) in feminine heads on Region 5 (Agreement target) in Experiment 1. Error bars indicate the standard error of the mean across participants.

The pre-critical Region 3 (Supplementary Material S7) and Region 4 (Attractor) did not show significant effects. This demonstrates that attractor mismatch conditions do not show increased RTs compared to the match ones due to mismatch. In Region 5 (agreement target), a main effect of Attractor was observed reflecting shorter RTs in the attractor mismatch conditions compared to the attractor match conditions. Crucially, the interaction between Grammaticality and Attractor and the attraction model were both significant, reflecting a facilitation in RTs in the ungrammatical mismatch condition. Pairwise comparisons (significance level adjusted to *p* = 0.013 using Bonferroni correction) showed that an ungrammatical match received longer RTs than a grammatical match (β = 0.127, *SE* = 0.033, *t* = 3.83, *p* < 0.001), while this was not the case for the mismatch conditions (grammatical mismatch vs. ungrammatical mismatch: β = 0.009, *SE* = 0.032, *t* = 0.30, *p* = 0.767). Crucially, the attraction effect only occurred in the ungrammatical sentences, as Table 2 shows, with a facilitation in RTs for the ungrammatical mismatch compared to the ungrammatical match. The grammatical sentences were not affected by attractor mismatch (β = −0.013, *SE* = 0.049, *t* = −0.27, *p* = 0.793) confirming attraction in ungrammatical sentences only, as predicted by retrieval accounts. On Region 6 (first post-critical region), an effect of Grammaticality was found, such that ungrammatical sentences overall exhibited longer RTs compared to the grammatical ones. Since feminine heads with neuter attractors exhibited attraction on Region 5 (Agreement target), the role of phonological matching was also explored. Figure 2 shows mean RTs by condition.

The model detected a main effect of Attractor (β = −0.144, *SE* = 0.035, *t* = −4.117, *p* < 0.001) confirming the initial model of attraction. However, Phonological matching (β = 0.101, *SE* = 0.072, *t* = 1.40, *p* = 0.176), and, crucially, the interaction between Phonological matching and Attractor (β = −0.025, *SE* = 0.072, *t* = 0.10, *p* = 0.725) were not significant, reflecting that phonological matching between the attractor and the agreement target did not lead to greater facilitation in RTs when attraction occured.

##### Neuter heads

Figure 1 shows mean RTs and standard error of the mean by condition and region. In the pre-critical Region 3 (Head region), there was a significant effect of Attractor, such that attractor match conditions had shorter RTs than attractor mismatch conditions (Attractor: β = 0.080, *SE* = 0.032, *t* = 2.53, *p* = 0.016). Region 4 showed that this effect was facilitated by an interaction with Grammaticality. Pairwise comparisons confirmed this finding (grammatical match vs. ungrammatical match: β = −0.167, *SE* = 0.036, *t* = −4.63, *p* < 0.001; grammatical mismatch vs. ungrammatical mismatch: β = 0.049, *SE* = 0.037, *t* = 1.34, *p* = 0.180; grammatical match vs. grammatical mismatch: β = −0.064, *SE* = 0.038, *t* = −1.70, *p* = 0.089; ungrammatical match vs. ungrammatical mismatch: β = 0.140, *SE* = 0.036, *t* = 3.86, *p* < 0.001). Region 5 (Agreement target) showed a similar significant interaction too, which, however, was toward the opposite direction of attraction (facilitation in the ungrammatical match instead of the ungrammatical mismatch). Region 6 showed a main effect of Grammaticality demonstrating that the ungrammatical conditions exhibited longer RTs compared to the grammatical ones on this region. Region 7 also showed a main effect of Attractor showing that the mismatch conditions received longer RTs compared to the match ones.

#### Discussion

The results confirm the presence of attraction at the nominal domain in Greek comprehension. Furthermore, attraction affected only ungrammatical sentences confirming retrieval accounts. Moreover, the fact that attraction occurred with the unmarked value (neuter) could not be easily captured under a representational account which predicts attraction only with marked/feminine attractors. When looking at neuter heads, the results are less clear because the significant effect on the pre-critical region (Region 3) and the interaction in the Attractor region (Region 4) were unexpected. This interaction on Region 4 remained significant on Region 5 (agreement target) too, and its direction was the opposite one from what was predicted if attraction had occurred. Given that Region 3 occurs before the critical regions (Region 4 and Region 5) and that ungrammaticality occurs only when participants reach Region 5, no differences of Attractor/Grammaticality for Region 3 and of Grammaticality for Region 4 were expected. Consequently, it is likely that the effects found on these regions are spurious (Patson and Husband, 2016, p. 956) and possibly responsible for the absence of attraction on the critical region.

### Experiment 2

#### Methodology

##### Participants

The same participants who participated in Experiment 1 also participated in Experiment 2, however, in a different session. The order of experiments by session was counterbalanced and there was at least a 2-week interval between the two sessions.

##### Materials and Design

Experiment 2 had a similar design and materials to Experiment 1. The crucial modification here was the target of the agreement. In this experiment, agreement on pronominal reference was tested by using object-clitic targets. In this experiment, the verb of the introductory sentence was in past-tense and imperfective aspect (three verbs were equally distributed across items: *epsahne, anazituse, jireve* “was looking for”) and the verb following the object-clitic target was in past-tense and perfective aspect (five verbs were equally distributed across items: *vrike* “found,” *eide* “saw,” *entopise* “found,” *anaklipse* “discovered,” *adikrise* “countered”). The same variables as in Experiment 1 were manipulated. The object-clitic was presented in the same region with the verb because they form one phonological unit. Table 3 shows a complete item across conditions for feminine and neuter heads, respectively.

**TABLE 3:** A complete item for feminine and neuter heads in Experiment 2. R stands for regions.

R1-2 *Introduction*	´Oταν o Γι$\overset{´}{\alpha }$ννησμπ$\overset{´}{\eta }$κεστηνκoυζ$\overset{´}{\iota }$να, $\overset{´}{\varepsilon }$ψαχνε… When the John went in-the kitchen, was looking for…		
	
*Feminine heads*	R3 - HEAD	R4 - ATTRACTOR	R5 - TARGET
grammatical match	τησυνταγ$\overset{´}{\eta }$ the_(FEM)_ recipe_(FEM)_	γιατηνπ$\overset{´}{\iota }$τσα for the_(FEM)_ pizza_(FEM)_	καιτηβρ$\overset{´}{\eta }$κε and it_(FEM)_ found
ungrammatical match	τησυνταγ$\overset{´}{\eta }$ the_(FEM)_ recipe_(FEM)_	γιατηνπ$\overset{´}{\iota }$τσα for the_(FEM)_ pizza_(FEM)_	καιτo βρ$\overset{´}{\eta }$κε and it_(NEUT)_ found
grammatical mismatch	τησυνταγ$\overset{´}{\eta }$ the_(FEM)_ recipe_(FEM)_	γιατo ψωμ$\overset{´}{\iota }$ for the_(NEUT)_ bread_(NEUT)_	καιτηβρ$\overset{´}{\eta }$κε and it_(FEM)_ found
ungrammatical mismatch	τησυνταγ$\overset{´}{\eta }$ the_(FEM)_ recipe_(FEM)_	γιατo ψωμ$\overset{´}{\iota }$ for the_(NEUT)_ bread_(NEUT)_	καιτo βρ$\overset{´}{\eta }$κε and it_(NEUT)_ found
R6-8 Continuation	π$\overset{´}{\alpha }$νωστo τραπ$\overset{´}{\varepsilon }$ζιτησκoυζ$\overset{´}{\iota }$νασ.		
	on the table of the kitchen.		
“When John went into the kitchen, he was looking for the recipe for the pizza/bread and he found it on the kitchen table.”			

**R1-2 *Introduction***	**´Oταν o Γι$\overset{´}{\alpha }$ννησμπ$\overset{´}{\eta }$κεστηνκoυζ$\overset{´}{\iota }$να, $\overset{´}{\varepsilon }$ψαχνε…** **When the John went in-the kitchen, was looking for…**		
	
***Neuter heads***	**R3 - HEAD**	**R4 - ATTRACTOR**	**R5 - TARGET**

grammatical match	τo κoυτ$\overset{´}{\alpha }$λι the_(NEUT)_ spoon_(NEUT)_	γιατo γλυκó for the_(NEUT)_ dessert_(NEUT)_	καιτo βρ$\overset{´}{\eta }$κε and it_(NEUT)_ found
ungrammatical match	τo κoυτ$\overset{´}{\alpha }$λι the_(NEUT)_ spoon_(NEUT)_ spoon_(NEUT)_	γιατo γλυκó for the_(NEUT)_ dessert_(NEUT)_	καιτηβρ$\overset{´}{\eta }$κε and it_(FEM)_ found
grammatical mismatch	τo κoυτ$\overset{´}{\alpha }$λι the_(NEUT)_ spoon_(NEUT)_	γιατησo$\overset{´}{\upsilon }$πα for the_(FEM)_ soup_(FEM)_	καιτo βρ$\overset{´}{\eta }$κε and it_(NEUT)_ found
ungrammatical mismatch	τo κoυτ$\overset{´}{\alpha }$λι the_(NEUT)_ spoon_(NEUT)_	γιατησo$\overset{´}{\upsilon }$πα for the_(FEM)_ soup_(FEM)_	καιτηβρ$\overset{´}{\eta }$κε and it_(FEM)_ found
R6-8 Continuation	π$\overset{´}{\alpha }$νωστo τραπ$\overset{´}{\varepsilon }$ζιτησκoυζ$\overset{´}{\iota }$νασ.		
	on the table of the kitchen.		
“When John went into the kitchen, he was looking for the spoon for the dessert/soup and he found it on the kitchen table.”			

#### Procedure

Experiment 2 had the same procedure as Experiment 1. The sentences were also divided into eight regions, as in example (4). The duration of the sound files did not differ on the Attractor between the match and the mismatch condition and the same applies to the duration of the agreement target (Region 5) between the grammatical and ungrammatical agreement target (Supplementary Materials S2.1.2, S2.2.2). Experiment 2 lasted approximately 40 min.

**Figure:**
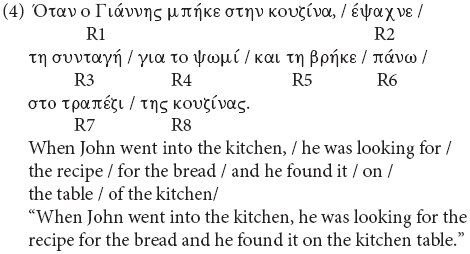


#### Predictions

The same predictions holding for Experiment 1 hold also for Experiment 2.

#### Analysis

The same analyses used for Experiment 1 were also used for Experiment 2.

#### Results

**FIGURE 3:**
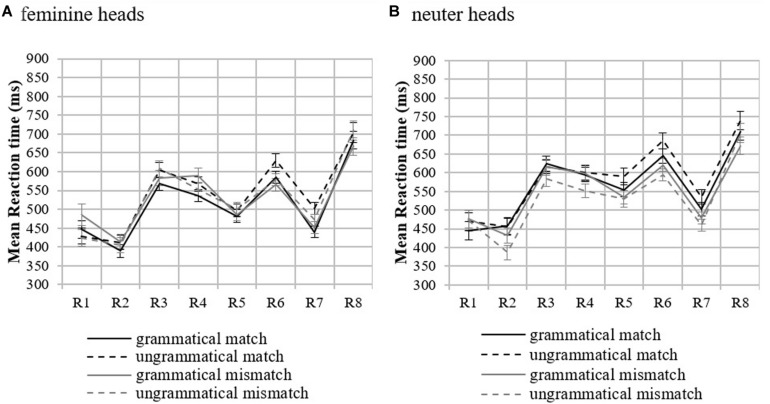
Mean reaction time by condition in feminine **(A)** and neuter **(B)** heads in Experiment 2. Error bars indicate the standard error of the mean across participants. R on the *x*-axis stands for Region. *Region 3*: Head, *Region 4*: Attractor, *Region 5*: Agreement target (past-participle), *Region 6*: first post-critical region, *Region 7*: second post-critical region and *Region 8*: sentence-final region.

No significant differences by condition were found in accuracy to the comprehension questions (Supplementary Material S6). Thus, we proceeded with the RT analyses. Figure 3 shows participants’ mean RTs and standard error of the mean by condition and region in both feminine and neuter heads. Table 4 reports the results of the mixed-effects models in the log-transformed RTs for Regions 4–7.

**TABLE 4:** Linear mixed-effects model results in Experiment 2 with feminine and neuter heads.

Feminine Heads								
	Region 4 (Attractor)				Region 5 (Agreement target)			
	β	SE	t	p	β	SE	t	p
Grammaticality	–0.003	0.032	–0.10	0.925	0.019	0.030	0.64	0.523
Attractor	0.023	0.032	0.71	0.485	–0.004	0.027	–0.16	0.873
Grammaticality:Attractor	–0.070	0.057	–1.23	0.227	–0.004	0.053	–0.83	0.407
*Attraction model*	–0.17	0.034	–0.49	0.624	–0.052	0.033	–1.57	0.118

	**Region 6 (post-critical 1)**				**Region 7 (post-critical 2)**			
	**β**	**SE**	**t**	**p**	**β**	**SE**	**t**	**p**

Grammaticality	0.028	0.023	1.23	0.224	0.082	0.037	2.22	0.037
Attractor	–0.040	0.024	–1.70	0.095	–0.010	0.028	–0.35	0.730
Grammaticality:Attractor	–0.073	0.040	–1.85	0.065	–0.084	0.062	1.35	0.192
*Attraction model*	–0.077	0.029	–2.65	0.008	–0.038	0.040	–0.96	0.354

**Neuter heads**								

	**Region 4 (Attractor)**				**Region 5 (Agreement target)**			
	**β**	**SE**	**t**	**p**	**β**	**SE**	**t**	**p**

Grammaticality	–0.029	0.032	–0.91	0.375	–0.020	0.050	–0.41	0.687
Attractor	–0.033	0.050	–0.66	0.518	–0.076	0.034	–2.25	0.036
Grammaticality:Attractor	–0.107	0.065	–1.65	0.117	–0.080	0.057	–1.41	0.159
*Attraction model*	–0.093	0.064	–1.46	0.160	–0.112	0.044	–2.54	0.011

	**Region 6 (post-critical 1)**				**Region 7 (post-critical 2)**			
	**β**	**SE**	**t**	**p**	**β**	**SE**	**t**	**p**

Grammaticality	0.006	0.033	0.18	0.856	–0.003	0.042	–0.08	0.940
Attractor	–0.058	0.037	–1.60	0.125	–0.072	0.038	1.89	0.073
Grammaticality:Attractor	–0.061	0.056	–1.09	0.287	–0.089	0.081	–1.10	0.284
*Attraction model*	–0.089	0.030	–2.96	0.005	–0.119	0.037	–3.23	0.001

##### Feminine heads

**FIGURE 4:**
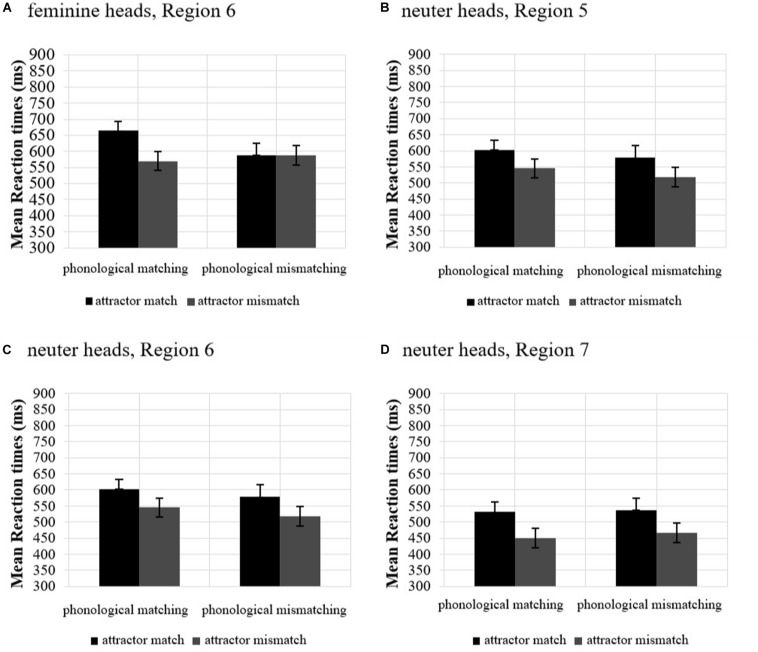
Mean reaction times in ungrammatical sentences by *Attractor* (match, mismatch) and *Phonological matching* (phonological matching/mismatching) in feminine heads, Region 6 **(A)**, neuter heads, Region 5 **(B)**, neuter heads, Region 6 **(C)**, and neuter heads, Region 7 **(D)** in Experiment 2. Error bars indicate the standard error of the mean across participants.

Region 3 (pre-critical region), Region 4 (Attractor), and Region 5 (Agreement target) did not show any significant effects. However, in Region 6 (post-critical 1) the interaction between Grammaticality and Attractor was approaching significance and the attraction model was significant, reflecting a facilitation in RTs in the ungrammatical mismatch condition. Grammatical sentences were not affected by attractor mismatch (grammatical match vs. grammatical mismatch: β = 0.001, *SE* = 0.027, *t* = 0.05, *p* = 0.957) confirming attraction only in ungrammatical sentences, as predicted by retrieval accounts. In Region 7 (post-critical 2), a main effect of Grammaticality showed that the ungrammatical sentences received longer RTs than the grammatical ones, reflecting that participants were sensitive to ungrammaticality. The role of Phonological matching in modulating the attraction pattern was also explored. Figure 4A shows mean RTs in ungrammatical match/mismatch conditions by Phonological matching.

There was an effect of Attractor (β = −0.078, *SE* = 0.033, *t* = −2.39, *p* = 0.021). However, Phonological matching (β = −0.070, *SE* = 0.048, *t* = −1.46, *p* = 0.156) and the interaction between Phonological matching and Attractor (β = 0.097, *SE* = 0.068, *t* = 1.44, *p* = 0.157) were not significant; Phonological matching did not facilitate RTs in the ungrammatical mismatch condition.

##### Neuter heads

No differences were found in Regions 3 and 4. In Region 5, there was a main effect of Attractor, such that attractor match received longer RTs than mismatch. Crucially, the attraction models were significant on Regions 5, 6, and 7, reflecting attraction in ungrammatical sentences (facilitation in RTs for the ungrammatical mismatch). Crucially, grammatical mismatch sentences did not show increased RTs compared to grammatical match sentences (Region 5: = −0.042, *SE* = 0.036, *t* = −1.56, *p* = 0.248, Region 6: = −0.031, *SE* = 0.031, *t* = −1.01, *p* = 0.312; Region 7: = −0.033, *SE* = 0.038, *t* = −0.86, *p* = 0.389). Since attraction was found, the role of Phonological matching was also explored. Figures 4B–D shows mean RTs in ungrammatical match/mismatch condition by Phonological matching. There was an effect of Attractor (Region 5: β = −0.113, *SE* = 0.044, *t* = −2.55, *p* = 0.011, Region 6: β = −0.094, *SE* = 0.030, *t* = −3.104, *p* = 0.003, Region 7: β = −0.120, *SE* = 0.037, *t* = −3.242, *p* = 0.001) confirming what the comparisons above had captured. Phonological matching (Region 5: β = −0.068, *SE* = 0.069, *t* = −0.986, *p* = 0.336, Region 6: β = 0.009, *SE* = 0.055, *t* = 0.162, *p* = 0.873, Region 7: β = 0.090, *SE* = 0.076, *t* = 1.181, *p* = 0.252) and crucially the interaction between Phonological matching and Attractor were not significant (Region 5: β = 0.027, *SE* = 0.089, *t* = 0.306, *p* = 0.760, Region 6: β = 0.105, *SE* = 0.061, *t* = 1.718, *p* = 0.090, Region 7: β = 0.014, *SE* = 0.076, *t* = 0.189, *p* = 0.850).

#### Discussion

The results of Experiment 2 showed that attraction occurs with feminine heads in line with what was found in Experiment 1, as well as with neuter heads. Thus, similarly to adjectival predicates, attraction on object-clitics occurred only in ungrammatical sentences in line with retrieval accounts, and both feminine and neuter heads were affected.

### Discussion Study 1

Overall, Study 1 showed that agreement attraction occurs in Greek during real-time sentence comprehension. Furthermore, most comprehension studies with gender attraction have focused on the verbal domain (e.g., Slioussar and Malko, 2016). Our study shows that the nominal domain is also prone to gender agreement attraction throughout the time course of sentence comprehension. Our study also shows that object-clitics are prone to gender attraction in comprehension as well (see Santesteban et al. (2017) for number attraction with clitics in Basque). Overall, our findings suggest that grammatical gender in inanimate nouns can induce attraction too, in line with previous studies (e.g., Slioussar and Malko, 2016). The fact that an uninterpretable feature (gender in inanimate nouns), i.e., without semantic content, can cause attraction speaks directly to the stage of processing where grammatical illusions arise, namely at the feature-checking stage. Consequently, our results suggest that attraction does not arise at the conceptual level and exhibits itself even in the absence of this level (e.g., Acuña-Fariña et al., 2014; Slioussar and Malko, 2016). The fact that phonological matching did not influence attraction reflects that the similarity of morphophonological cues between the attractor and the agreement target is not decisive in attraction. Finally, adult native speakers of Greek were sensitive to gender agreement violations in the ungrammatical match condition with both adjectival predicates (in both feminine and neuter heads) and object-clitics (in feminine heads), demonstrating that they process gender on both agreement targets.

## Study 2

Study 2 targeted participants’ timed and untimed judgments in order to test whether gender agreement attraction influences end-of-sentence measurements in comprehension. In the following experiments, the same materials as in Study 1 were used for reasons of consistency and comparability.

### Experiment 3

Experiment 3 was a speeded Grammaticality Judgement task (s-GJT) and targeted participants’ ability in judging sentences with gender agreement attraction under time pressure.

#### Methodology

##### Participants

The participants’ pool consisted of 37 healthy adult native speakers of Greek (mean age = 21.6, age range = 18–32, 18 female).

##### Materials and Design

The materials included the experimental sentences from Experiment 1 (adjectival predicates) and Experiment 2 (object-clitics). The design, manipulations, and number of items were all similar to Experiment 1 and Experiment 2 (Tables 1, 2). Thus, 48 out of 96 experimental sentences were ungrammatical. Additionally, 104 filler sentences were constructed; half of them were ungrammatical including a variety of violations. The agreement target was in sentence-final position following the previous literature of s-GJTs (e.g., Tanner et al., 2014; Lago et al., 2018; Hammerly et al., 2019) and an adverb (Supplementary Material S5) was also added before the agreement target to increase the processing demands of the task (“John was looking for the recipe for the bread and (he) eventually found it”).

#### Procedure

All participants were tested individually in a quiet room at the Aristotle-University of Thessaloniki. Participants were told that their task was to read and judge sentences in Greek fast. Then they read the written instructions on the computer screen. They were also instructed to judge the sentences as fast and accurate as they could. There were six practice trials at the beginning of the task, half of them ungrammatical. The practice trials did not include violations of gender agreement and participants were receiving feedback on their accuracy only during the practice trials. The stimuli were visually presented on a laptop. The sentences were presented one word at a time at the center of the screen. Each word remained on the screen for 250 ms plus 25 ms further for each character to compensate for length differences. The participants had to press the space button on the keyboard to start reading a sentence whenever they were ready. A fixation cross (remaining on the screen for 1,500 ms) was preceding the first word to warn participants that a new trial was about to start. After the end of the sentence, a question mark appeared at the center of the screen and participants had to indicate whether the sentence was grammatical or ungrammatical by pressing the green (grammatical) or the red (ungrammatical) button on the keyboard (K and D keys, respectively). If participants failed to respond within 1,700 ms from the end of a trial, a warning appeared on the screen to let them know that they were too slow. During the task, no feedback was given for their accuracy. Overall, the session lasted approximately 30 min.

#### Predictions

Attractor mismatches between the gender value of the head and the attractor are expected to influence accuracy scores if attraction occurs. If attraction affects ungrammatical sentences only, lower accuracy in rejecting the ungrammatical mismatch condition is expected compared to the ungrammatical match condition. Alternatively, if attraction affects grammatical sentences too, lower accuracy in accepting the grammatical mismatch condition compared to the grammatical match condition is also expected. Moreover, if gender markedness modulates attraction patterns, then attraction is more likely to occur with marked attractors (e.g., neuter heads-feminine attractors) than with unmarked attractors (feminine heads-neuter attractors). Also, based on the findings of Experiment 1, phonological matching should not modulate attraction.

#### Analysis

A 70% accuracy threshold was set as an inclusion criterion. All participants scored above this threshold. Additionally, trials in which participants exceeded the response deadline (RTs > 1,700 ms) were deleted, influencing less than 3.6% of the dataset (Supplementary Material S3). In all cases, following the current literature on agreement attraction we report (a) the results from the logistic regression models with Grammaticality and Attractor as fixed effects with orthogonal contrasts (Grammaticality: *grammatical* = 0.5, *ungrammatical* = −0.5, Attractor: *match* = 0.5, *mismatch* = −0.5) as well as their interaction, and (b) planned pairwise comparisons employing logistic regression models between the grammatical match and the grammatical mismatch conditions and between the ungrammatical match and the ungrammatical mismatch conditions, given that the theoretical questions of interest crucially depend on whether attraction influences both grammatical and ungrammatical sentences or only ungrammatical sentences (e.g., Lago et al., 2015; Hammerly et al., 2019;, p. 12; Tanner et al., 2014, p. 12). Attractor was coded as a fixed effect in the pairwise comparisons, and the significance level was corrected for multiple comparisons using Bonferroni correction (significance level adjusted to *p* = 0.025). A model of Phonological matching was also fit, when attraction in ungrammatical sentences was significant, including Attractor (*match* = 0.5, *mismatch* = −0.5) and Phonological matching (*phonological matching* = 0.5, *phonological mismatching* = −0.5) as well as their interaction.

#### Results

The mean accuracy in the filler sentences was 89% (SD = 31, range = 78–97). Table 5 shows participants’ accuracy by condition and Table 6 reports the results of the mixed-effects models.

**TABLE 5:** Participants’ mean accuracy and standard error in adjectival predicates and object-clitics in Experiment 3.

	Grammatical match	Ungrammatical match	Grammatical mismatch	Ungrammatical mismatch
**Adjectival predicates**				
Feminine heads	93 (1.79)	95 (1.41)	88 (2.23)	94 (1.61)
Neuter heads	90 (2.05)	98 (1.01)	83 (2.56)	89 (2.09)
**Object-clitics**				
Feminine heads	95 (1.49)	84 (2.45)	90 (2.04)	72 (3.05)
Neuter heads	92 (1.89)	94 (1.65)	90 (2.05)	80 (2.76)

**TABLE 6:** Linear mixed-effects model results of participants’ accuracy in adjectival predicates and object-clitics in Experiment 3.

	Feminine heads				Neuter heads			
	β	SE	z	*p*	β	SE	*z*	*p*
**Adjectival predicates**								
Grammaticality	–1.011	0.815	–1.24	0.215	–1.102	0.296	–3.72	<0.001
Attractor	0.482	0.773	0.62	0.533	1.147	0.295	3.88	<0.001
Grammaticality:Attractor	0.588	1.599	0.37	0.713	–1.023	0.589	–1.74	0.083
**Object-clitics**								
Grammaticality	1.113	0.346	3.22	0.001	0.252	0.242	1.04	0.297
Attractor	0.852	0.335	2.54	0.011	0.831	0.242	3.43	0.001
Grammaticality:Attractor	–0.134	0.630	–0.21	0.831	–1.245	0.489	–2.57	0.010

##### Adjectival predicates

###### Feminine heads

The logistic regression model and the pre-planned pairwise comparisons did not detect any significant differences.

###### Neuter heads

The model revealed a main effect of Grammaticality, such that participants were more accurate in rejecting ungrammatical sentences than accepting grammatical sentences and a main effect of Attractor, demonstrating that attractor mismatch decreased accuracy. The planned pairwise comparisons confirmed attraction in ungrammatical sentences; accuracy in ungrammatical sentences decreased due to attractor mismatch (β = 1.646; *SE* = 0.509; *z* = 3.23; *p* = 0.001), reflecting that participants incorrectly gave more “grammatical” responses for the ungrammatical mismatch condition. In grammatical sentences, accuracy lowered due to attractor mismatch by 7% (β = 0.636; *SE* = 0.299; *z* = 2.13; *p* = 0.033), although the difference was not significant (*p* > 0.025). Table 7 shows mean accuracy in ungrammatical match conditions grouped by Phonological matching.

**TABLE 7:** Participants’ mean accuracy and standard error in Experiment 3 grouped by Phonological matching.

	Adjectival predicates, neuter heads		Object-clitics, neuter heads		Object-clitics, feminine heads	
	Attractor match	Attractor mismatch	Attractor match	Attractor mismatch	Attractor match	Attractor mismatch
Phonological matching	96 (1.81)	88 (3.07)	87 (3.30)	74 (4.24)	91 (2.78)	78 (4.06)
Phonological mismatching	99 (0.91)	91 (2.83)	82 (3.61)	70 (4.39)	97 (1.67)	81 (3.76)

The model of Phonological matching in ungrammatical sentences revealed a main effect of Attractor (β = 1.860; *SE* = 0.61; *z* = 3.05; *p* = 0.002) confirming the existence of attraction. However, no effect of Phonological matching was detected (β = 0.873; *SE* = 0.657; *z* = 1.33; *p* = 0.184) and no interaction with Attractor (β = 1.169; *SE* = 1.219; *z* = 0.96; *p* = 0.338), i.e., Phonological matching did not increase attraction errors.

##### Object-clitics

###### Feminine heads

The model revealed a main effect of Grammaticality demonstrating that participants were less accurate in rejecting ungrammatical sentences than accepting grammatical sentences. There was a main effect of Attractor showing a decrease in the participants’ accuracy in the mismatch conditions. In the planned pairwise comparisons the ungrammatical sentences seemed to be modulated by attractor mismatch (β = 0.857; *SE* = 0.257; *z* = 3.34; *p* < 0.001), but this was not the case in grammatical sentences (β = 0.767; SE = 0.398; *z* = 1.99; *p* = 0.054). Table 7 shows mean accuracy in ungrammatical match conditions grouped by Phonological matching. The model of Phonological matching in ungrammatical sentences revealed a main effect of Attractor (β = 0.924; *SE* = 0.403; *z* = 2.29; *p* = 0.022) confirming the existence of attraction. However, no effect of Phonological matching was detected (β = −0.312; *SE* = 0.352; *z* = −0.89; *p* = 0.375) and no interaction with Attractor (β = −0.275; *SE* = 0.618; *z* = −0.45; *p* = 0.657).

###### Neuter heads

The model revealed a main effect of Attractor demonstrating that the mismatch condition lowered participants’ accuracy. Crucially, the effect was modulated by a significant interaction with Grammaticality. Planned pairwise comparisons revealed that attractor mismatch decreased the accuracy rate in the ungrammatical condition but not in the grammatical one (ungrammatical: (β = 1.52; *SE* = 0.359; *z* = 4.24; *p* < 0.001, grammatical: β = 0.216; *SE* = 0.337; *z* = 0.64; *p* = 0.521). Table 7 shows mean accuracy in the ungrammatical match conditions grouped by Phonological matching. The model of Phonological matching in ungrammatical sentences revealed a main effect of Attractor (β = 1.926; *SE* = 0.667; *z* = 2.89; *p* = 0.004) confirming the existence of attraction. However, no effect of Phonological matching was detected (β = 0.655; *SE* = 0.553; *z* = 1.19; *p* = 0.236) and no interaction with Attractor (β = 0.682; *SE* = 0.887; *z* = 0.77; *p* = 0.442).

#### Discussion

The results of Experiment 3 showed that gender attraction occurs in timed judgments, in both feminine and neuter heads, demonstrating that both marked and unmarked attractors cause attraction in line with Experiment 2. Attraction affected only ungrammatical sentences in line with retrieval accounts and with what was found in Study 1. Although grammatical sentences seemed to be affected by attractor mismatches (e.g., participants’ tendency to reject grammatical sentences with mismatch in the neuter heads), this effect was not statistically reliable. Thus, attractor mismatches affected ungrammatical sentences only, confirming retrieval accounts.

### Experiment 4

Experiment 4 was an auditory scaled Acceptability Judgment Task (AJT), similar to the one used by Fuchs et al. (2015) and tested whether gender attraction is evident in untimed scaled judgments.

#### Methodology

##### Participants

The participants’ pool consisted of 32 healthy adult native speakers of Greek (mean age = 20.4, age range = 18–24, 14 female).

##### Materials and design

Similarly to Experiment 3, the materials consisted of the materials of Experiment 1 and 2 (plus regions 6–8). Given that the task measured scaled judgments, some new marginally ungrammatical fillers were created for balance (e.g., nominal ellipsis, weak and strong islands, etc.). Overall, there were 201 trials, 1/3 were ungrammatical and 1/3 marginally ungrammatical. The design, the materials, and the number of experimental items were all the same as in Experiment 3.

#### Procedure

All participants were tested in a quiet room at the Aristotle-University of Thessaloniki. The sentences were auditorily presented via headphones. The participants had to press the space button on the keyboard to listen to each trial. During the auditory presentation of a trial, the screen remained blank. After the end of the trial, a question mark appeared on the screen and participants had to judge the sentences on a 1–7 Likert scale by pressing one of the relevant keys on the keyboard (1 not acceptable/bad - 7 completely acceptable/very good). The task was untimed and participants could spend as much time as they wanted on their response and no feedback was given for their judgments. Participants were instructed from the experimenter that their task was to listen to and judge sentences in Greek on a 1–7 scale. Participants then listened to the prerecorded instructions. There were 6 practice trials at the beginning of the task, 2 grammatical, 2 ungrammatical, 2 marginally ungrammatical. The practice trials did not include violations of gender agreement and no feedback was given to the participants for the way they rated the sentences. Overall, the session lasted 40 min approximately.

#### Predictions

If attraction occurs, attractor mismatch should increase acceptability ratings in the ungrammatical mismatch condition compared to the ungrammatical match condition. If attraction influences grammatical sentences too, then attractor mismatch should decrease ratings in the grammatical mismatch condition compared to the grammatical match condition. Additionally, if participants are sensitive to gender agreement violations overall (i.e., ungrammatical match condition), they are expected to give overall significantly higher acceptability ratings to grammatical than ungrammatical sentences. Based on the findings of Experiment 1, Phonological matching is not expected to modulate attraction with adjectival predicates.

#### Analysis

For participants’ ratings linear mixed effects models were fit as in Experiment 1. Analyses and contrast coding were similar to Experiment 3.

#### Results

The mean ratings in the fillers was 6.2 (SD = 1.58, range = 3–7) for grammatical sentences, 4.5 (SD = 2.2, range = 2.8–5.6) for marginally ungrammatical sentences, and 2.96 (SD = 1.9, range = 1.3–5) for ungrammatical sentences. Table 8 reports the mean acceptability ratings by condition in feminine and neuter heads for both adjectival predicates and object-clitics and Table 9 reports the results of the mixed-effects models by gender value and structure. An additional analysis on the *z*-transformed ratings was also conducted showing similar results (Supplementary Material S9).

**TABLE 8:** Participants’ mean ratings and standard error in adjectival predicates and object-clitics in Experiment 4.

	Grammatical match	Ungrammatical match	Grammatical mismatch	Ungrammatical mismatch
**Adjectival predicates**				
Feminine heads	5.59 (0.13)	2.6 (0.13)	5.63 (0.13)	2.69 (0.13)
Neuter heads	5.38 (0.14)	2.41 (0.12)	5.27 (0.14)	2.51 (0.12)
**Object-clitics**				
Feminine heads	6.15 (0.10)	3.03 (0.13)	6.11 (0.11)	3.38 (0.15)
Neuter heads	5.88 (0.11)	2.88 (0.14)	6.02 (0.11)	3.25 (0.15)

**TABLE 9:** Linear mixed-effects model results of participants’ ratings in adjectival predicates and object-clitics in Experiment 4.

	Feminine heads				Neuter heads			
	β	SE	z	*p*	β	SE	*z*	*p*
**Adjectival predicates**								
Grammaticality	2.966	0.304	9.75	<0.001	2.868	0.257	11.15	<0.001
Attractor	–0.075	0.099	–0.76	0.454	0.018	0.104	0.17	0.864
Grammaticality:Attractor	0.022	0.222	0.10	0.920	0.230	0.239	0.96	0.342
**Object-clitics**								
Grammaticality	2.922	0.332	8.79	<0.001	2.884	0.317	9.10	<0.001
Attractor	–0.159	0.103	–1.54	0.140	–0.250	0.115	–2.16	0.041
Grammaticality:Attractor	0.402	0.204	1.97	0.061	0.231	0.236	0.99	0.337

##### Adjectival predicates

###### Feminine heads

The model showed a main effect of Grammaticality; the difference between the grammatical and ungrammatical conditions was significant. The effect of Attractor and the interaction between Grammaticality and Attractor were not significant. The pairwise comparisons did not show any differences either.

###### Neuter heads

The model revealed a main effect of Grammaticality with significantly higher ratings for grammatical than ungrammatical sentences and no significant interaction between Grammaticality and Attractor. The pairwise comparisons did not show any significant differences either (*p* > 0.025).

##### Object-clitics

###### Feminine heads

The model revealed a main effect of Grammaticality with significantly higher ratings for grammatical than ungrammatical sentences. The interaction between Grammaticality and Attractor was marginally significant, reflecting higher acceptability ratings in the ungrammatical mismatch condition than the ungrammatical match condition. The pairwise comparisons confirmed this tendency: only the ungrammatical sentences seemed to be modulated by attractor mismatch (β = −0.351; *SE* = 0.114; *t* = −3.09; *p* = 0.002), with higher ratings for the ungrammatical mismatch condition compared to the ungrammatical match condition. The ratings in the grammatical mismatch condition were not significantly lower compared to the grammatical match condition (β = 0.041; *SE* = 0.125; *t* = 0.33; *p* = 0.748), indicating attraction only in ungrammatical sentences. Table 10 shows ratings in ungrammatical conditions grouped by Phonological matching.

**TABLE 10:** Participants’ mean ratings and standard error in Experiment 4 grouped by Phonological matching.

	Object-clitics, feminine heads		Object-clitics, neuter heads	
	Attractor match	Attractor mismatch	Attractor match	Attractor mismatch
Phonological matching	2.92 (0.19)	3.16 (0.20)	3.12 (0.21)	3.43 (0.21)
Phonological mismatching	3.15 (0.19)	3.6 (0.21)	2.64 (0.17)	3.06 (0.21)

The model of Phonological matching in ungrammatical sentences revealed a main effect of Attractor (β = −0.334; *SE* = 0.115; *z* = −2.89; *p* = 0.006), confirming the existence of attraction. However, no effect of Phonological matching was detected (β = −0.0002; *SE* = 0.149; *z* = −0.0001; *p* = 0.999) and no interaction with Attractor was seen (β = −0.059; *SE* = 0.247; *z* = −0.24; *p* = 0.811).

###### Neuter heads

The model revealed a main effect of Grammaticality and a main effect of Attractor. The interaction did not reach significance. The pairwise comparisons confirmed attraction only in ungrammatical sentences (higher ratings in the ungrammatical mismatch compared to the ungrammatical match condition; β = −0.361; *SE* = 0.137; *t* = −2.64; *p* = 0.009). Grammatical mismatch did not show lower ratings than grammatical match (β = −0.134; *SE* = 0.135; *t* = −0.99; *p* = 0.331). Table 10 shows acceptability ratings in ungrammatical conditions grouped by Phonological matching. The model of Phonological matching in ungrammatical sentences revealed a main effect of Attractor (β = −0.360; *SE* = 0.137; *z* = −2.63; *p* = 0.009), confirming the existence of attraction. However, no effect of Phonological matching was detected (β = −0.404; *SE* = 0.280; *z* = −1.44; *p* = 0.163) and neither was any interaction with Attractor (β = −0.159; *SE* = 0.282; *z* = −0.57; *p* = 0.573).

#### Discussion

Experiment 4 shows gender agreement attraction in untimed judgments in Greek comprehension. Object-clitics revealed attraction in ungrammatical sentences only in both feminine and neuter heads (as in Experiments 2 and 3), confirming retrieval accounts. On the other hand, adjectival predicates were more resistant to attraction and only some small numerical trends were detected. This reflects that agreement target may play a role in end-of-sentence assessments where the sentence-level acceptability is measured. Additionally, the ungrammatical sentences were rated significantly lower than the grammatical ones in both gender values and in both structures.

### Discussion Study 2

Overall, Study 2 shows that attraction occurs in end-of-sentence measurements of sentence comprehension both in timed and untimed judgments. Attraction was found in both feminine and neuter heads for object-clitics but only for neuter heads in adjectival predicates. The analyses also revealed that attraction significantly affected ungrammatical sentences, creating illusions of grammaticality in line with retrieval accounts. Attraction also affected grammatical sentences but only numerically and only in timed judgments (see section “General discussion”). Another finding was that the similarity in the morphophonological realization of gender between the attractor and the agreement target did not increase attraction. Finally, adult native speakers of Greek are sensitive to gender agreement violations and they seem to react to violations with both agreement targets and with both gender values. However, we observe a numerical tendency such that ungrammatical neuter agreement targets are overall more accepted (with feminine heads) in object-clitics (match and mismatch conditions) than ungrammatical feminine targets (with neuter heads). Specifically, focusing only on the attractor match conditions in Experiment 3, the participants were 10% less accurate with feminine heads when combining with ungrammatical neuter object clitics (error rate: 16%) compared to neuter heads combining with ungrammatical feminine object-clitics (error rate: 6%). At the same time, the equivalent differences in the error rates in adjectival predicates were much lower (feminine heads with feminine predicates vs. with ungrammatical neuter targets: 5%, neuter heads with neuter targets vs. with ungrammatical feminine targets: 2%). A similar pattern was also observed in the acceptability judgments (Experiment 4). Feminine heads with ungrammatical neuter object-clitics were accepted with a 3.03 rating out of 7, a difference of 3.12 from the grammatical counterpart (feminine heads with feminine object-clitics), while at the same time, neuter heads with ungrammatical feminine object-clitics were accepted with a 2.88 rating out of 7, a difference of 3.0 from the grammatical counterpart (neuter heads with ungrammatical feminine object-clitics). The equivalent differences in the ratings in adjectival predicates were much lower (feminine heads with feminine predicates vs. with ungrammatical neuter targets: 2.99, neuter heads with neuter targets vs. with ungrammatical feminine targets: 2.77).

## General Discussion

This is the first gender attraction study in Greek that investigated attraction across two structurally different configurations, i.e., adjectival predicates and object-clitics, using similar methods and materials. Study 1 used real-time comprehension tasks to address whether Greek shows attraction during real-time sentence comprehension (Experiment 1) and whether object-clitics are prone to gender agreement attraction (Experiment 2). Study 2 targeted end-of-sentence comprehension, employing timed (Experiment 3) and untimed judgment (Experiment 4) tasks in order to investigate whether gender agreement attraction occurs under time pressure as well as in the absence of time pressure. This allowed us to address not only how participants process sentences in real-time but also how they judge the acceptability of sentences with attraction manipulations. The four experiments explored the processing strategy the parser aligns with during the comprehension of agreement attraction (the existence of attraction in grammatical vs. ungrammatical sentences, the markedness of the attractor, phonological matching, and the sensitivity to gender agreement rules in the absence of attraction).

### Gender Agreement Attraction: Representations and Processes

The results from the current studies add Greek to the cross-linguistic puzzle of gender agreement attraction in comprehension (Spanish: Martin et al., 2012, 2014; Acuña-Fariña et al., 2014; Fuchs et al., 2015; Cunnings et al., 2017;, Russian: Slioussar and Malko, 2016, Arabic: Tucker et al., 2016, French: Villata and Franck, 2019). The current findings support the idea that attraction is a universal phenomenon occurring cross-linguistically irrespective of the morphological richness of its language (Lago et al., 2015). Our present studies mainly support the existence of attraction in all measures of comprehension. This is important because previous work primarily tested the real-time processing of the phenomenon and little has been known for the impact of gender attraction in other measures of sentences comprehension (e.g., speeded judgments). Specifically, our work shows that adjectival predicates show attraction in real-time and in timed judgments but not in untimed judgments, as in Fuchs et al. (2015). On the other hand, clitics consistently show attraction in timed and untimed measures. Thus, these results suggest that object-clitics may be overall more vulnerable to attraction. We believe that there are two main reasons for that: first, our sentences with object-clitics are not compatible (as opposed to adjectival predicates) with predictive processing - participants could not expect earlier that an object-clitic is coming, and thus, they were more vulnerable to attraction. Second, the position of the gender cue in clitics is not so prominent as compared to adjectival predicates (Tables 1, 3): gender is immediately before the verb in which the clitic is phonologically attached to, while adjectival predicates mark gender on the suffix. On the other hand, predictive processing in adjectival predicates may decrease the chances of attraction. In any case, this work adds object-clitics in the agreement targets modulated by gender attraction in comprehension (see Santesteban et al., 2017 for number attraction with clitics).

Focusing now on the attraction patterns observed, the picture seems to be highly informative. Our findings are in line with previous studies on gender agreement attraction in comprehension; first, markedness of the attractor does not seem to play a role (Martin et al., 2012, 2014; Acuña-Fariña et al., 2014; Slioussar and Malko, 2016). Furthermore, the results mainly suggest that attraction occurs only in ungrammatical sentences. This is in line with retrieval accounts (e.g., cue-based retrieval account: Wagers et al., 2009). Regarding adjectival predicates, attractor mismatch overall affected ungrammatical sentences only, as in object-clitics. However, in speeded judgments (Experiment 3) attraction numerically affected grammatical sentences too. This numerical pattern is in line with representational accounts of agreement attraction, but also with predictive processing which has an impact on grammatical sentences too (e.g., Martin et al., 2012, 2014; Villata et al., 2018; for gender). However, we would not like to overinterpret this finding, given that this pattern was observed once and numerically only across all four experiments. Meanwhile, the previous body of work on gender attraction in comprehension has shown a mixed picture. Structures with ellipsis (Martin et al., 2012, 2014) and adjectival predicates (Acuña-Fariña et al., 2014) showed that attraction affected both grammatical and ungrammatical sentences, while only ungrammatical sentences were affected on the verb (Slioussar and Malko, 2016; Tucker et al., 2016). As Wagers et al. (2009) point out, attraction in grammatical sentences possibly stems from a different source and reflects some later processes; its effect is delayed and smaller than in ungrammatical sentences and end-of-sentence measures are particularly sensitive to such late processes of agreement attraction.

With respect to the presence of attraction, one of the reviewers asked why the presence of attraction is reversed in Study 1 vs. Experiment 3. The results are not completely reversed: Object-clitics do show attraction with both gender values in both Experiment 2 and Experiment 3. As for adjectival predicates, they show attraction with feminine heads in Experiment 1, and attraction with neuter heads in Experiment 3. There are two possible reasons for this reversed pattern: first, attraction may have been hidden in Experiment 1 due to the presence of the spurious effect in neuter heads, and second, the overall good performance on ungrammatical sentences in Experiment 3 may have lowered the attraction error rates in feminine heads.

Another implication of the current work is the lack of phonological matching effects of the attractor in attraction configurations, suggesting that attraction has syntactic grounds and is not influenced by the morphophonological similarity between the suffix of an unavailable syntactic constituent (attractor) and the suffix of the agreement target. In line with the argument in Slioussar and Malko (2016), the head noun seems to be more important than the attractor and only certain features of the latter play a role in modulating attraction: crucially, these features mostly affect attraction when they are similar to certain features of the head noun (e.g., syncretism/case-disambiguation) and not the agreement target which may be related with agreement *per se*, as the study by Marinis et al. (2017) shows for monolingual and bilingual children.

Overall, adult native speakers of Greek showed attraction across the different tasks employed, in real-time, speeded judgments, and untimed acceptability measurements, suggesting that although the effects of attraction might be greater or smaller depending on the task, the structure, and/or the time-pressure, the existence of attraction is not expected to be necessarily dependent on time. This is in line with what was recently found in the production of number attraction (Linzen and Leonard, 2018).

### Sensitivity to Gender Agreement Violations

The findings of the two studies suggest that adult native speakers of Greek are sensitive to gender agreement violations in all measures of sentence comprehension. Both RTs (Study 1) and end-of-sentence judgments (Study 2) demonstrate that speakers use their implicit linguistic knowledge of gender agreement to perform these tasks and they respect agreement rules, at least when there is no conflict between the gender cues of the head and the gender cues of the attractor. These results are in line with previous studies on gender agreement in comprehension (e.g., Spanish: Sagarra and Herschensohn, 2011; Acuña-Fariña et al., 2014; Fuchs et al., 2015; Russian: Slioussar and Malko, 2016). Focusing on Greek, Tzovara (2017) found that native speakers of Greek are sensitive to gender agreement rules with adjectival predicates by means of a self-paced reading task combined with end-of-sentence yes/no judgments. Tzovara reports accuracy and RTs from judgments, while she does not report RTs from the critical region (agreement target). The current studies converge with what Tzovara found for gender agreement sensitivity on adjectival predicates in participants’ judgments, while at the same time they also capture this sensitivity throughout the time course of sentence processing.

Another finding was that, although participants were highly accurate in their speeded judgments (when there was no interference from the attractor), they were more accurate in rejecting an ungrammatical sentence than accepting a grammatical one when the agreement target was an adjectival predicate. Notice that participants did not show this effect in object-clitics; instead, the effect in object-clitics was toward the opposite direction. Both structures were presented in each single experiment and the item presentation was pseudo-randomized. One explanation for the fact that participants were overall more accurate in rejecting ungrammatical sentences than accepting grammatical sentences could be that they were alert to violations since they were asked to do so; the task was explicit since the instructions were to judge the acceptability of the sentence (by a yes/no answer) under time constraints. However, the fact that the opposite effect was observed for object-clitics within the same experiment is puzzling, although it is possible that overall, object-clitics impose more burden to the parser and this is perhaps why the opposite effect was found in the latter configuration. A similar pattern has been also found in Häussler (2009; Experiment 1) for number agreement on the verb in German by means of the same method. Furthermore, Tanner et al. (2014) also found a similar numerical pattern (11% mean difference) for number agreement in English. In order to avoid an over-interpretation of the data at this point, we leave this issue open to further investigation.

Overall, as expected, the two studies show that adult native speakers of Greek are sensitive to gender agreement violations in comprehension in real-time and in end-of-sentences measurements of grammatical sensitivity (timed and untimed). However, numerically we observe that ungrammatical neuter agreement targets (with feminine heads) tend to be more accepted in the object-clitic conditions (match and mismatch conditions) than ungrammatical feminine targets (with neuter heads), which was also observed in Bañón and Rothman (2016) and Fuchs et al. (2015) when the agreement target bears the default gender value. A similar pattern has also been observed in production errors during the acquisition of Greek object-clitics (Mastropavlou, 2006, p. 279–281). This tendency is perhaps related to the fact that object-clitics are also a spell-out of the phi-features (Tsimpli and Stavrakaki, 1999) where neuter bears a default value and may not trigger agreement checking or may impose delays in the parser (i.e., more time for agreement checking to be completed). This is also supported by the fact that object-clitics share the same forms with definite articles in Greek and are, thus, ambiguous at the level of form. Overall, it seems that both gender values are activated in Greek across all measures of comprehension.

## Conclusion

Study 1 and 2 share evidence for gender agreement attraction in Greek. The pattern found is primarily predicted by retrieval accounts of attraction; in ungrammatical sentences participants exhibited a facilitation in RTs due to attractor mismatch, lower accuracy in rejecting these sentences in speeded binary judgments, and also higher acceptability ratings in untimed acceptability judgments. Additionally, similar morphophonological cues between the agreement target and the attractor do not increase attraction, reflecting that the morphophonological realization of the attractor relative to the agreement target does not matter. Finally, both feminine and neuter seem to be available in the feature content repertoire of Greek adults, since participants are sensitive to these violations. However, the agreement target also seems to play a role in regulating this sensitivity with ungrammatical neuter object-clitics being relatively more acceptable, reflecting the default and underspecified status of neuter in Greek inanimate nouns.

## Data Availability Statement

The datasets generated for this study are available on request to the corresponding author.

## Ethics Statement

All participants gave written informed consent in accordance with the Declaration of Helsinki. The study has been reviewed by the Ethics Committee of the Deutsche Gesellschaft für Sprachwissenschaft (German Linguistics Association) and has been given a favorable ethical opinion for conduct.

## Author Contributions

Both authors conceived and designed the study and wrote the manuscript. AP transcribed and coded the data, oversaw the implementation of the study, collected the data, and conducted the statistical analyses of the data.

## Conflict of Interest

The authors declare that the research was conducted in the absence of any commercial or financial relationships that could be construed as a potential conflict of interest.

## Footnotes

1 Note that under inhibitory interference in the similarity-based interference accounts, the opposite direction is predicted here, as a reviewer points out, i.e., longer RTs for the grammatical match condition compared to the ungrammatical match condition. However, this prediction has not been confirmed with empirical findings or even in recent metanalyses for agreement dependencies as the ones we test here [see Jäger et al. (2017) and Cunnings and Sturt (2018) for discussion]. Rather, the prediction is more relevant to long-distance dependencies combined with processes and cues, such as, agent identification and animacy.
