# Supplementary material for: Gender Agreement Attraction in Greek Comprehension
Source: Front Psychol. 2020 Apr 29;11:717. doi: 10.3389/fpsyg.2020.00717 (PMC7201047; doi:10.3389/fpsyg.2020.00717)
Supplement: Supplementary file 1 [file Data_Sheet_1.docx]

Supplementary Material

# Differences in frequency between the nouns in the attractor match and the attractor mismatch conditions:

feminine nouns: mean matching attractors = 0.058 vs. mean mismatching attractors = 0.029, t(23) = 1.33, p = .205;

neuter nouns: mean matching attractors = 0.036 vs. mean mismatching attractors = 0.065, t(23) = -0.76, p = .457).

# Duration of sound files in Experiment 1 and 2 in the attractor match vs. the attractor mismatch conditions on Region 4 and Region 5.

## Region 4

### Experiment 1

Feminine head nouns: mean duration (in milliseconds) of attractor match = 1018, mean duration of attractor mismatch: = 999, (t = 1.60; p = .114); neuter head nouns: mean duration of attractor match = 1013, mean duration of attractor mismatch= 1016, (t = -0.24; p = .809);

### Experiment 2

Feminine head nouns: mean duration of attractor match= 999, mean duration of attractor mismatch= 979, (t = 0.620; p = .536); neuter head nouns: mean duration of attractor match = 999, mean duration of attractor mismatch = 996, (t = 3.22; p = .748).

## Region 5

### Experiment 1

Feminine head nouns: mean duration of grammatical target = 898, mean duration of ungrammatical target = 907, (t = -40.48; p = .630); neuter head nouns: mean duration of grammatical target = 899, mean duration of ungrammatical target = 895, (t = 0.19; p = .854).

### Experiment 2

Feminine head nouns: mean duration of grammatical target = 1030, mean duration of ungrammatical target = 984, (t = 1.29; p = .200); neuter head nouns mean duration of grammatical target = 968, mean duration of ungrammatical target = 1061, (t = 0.185; p = .854).

# Data loss in Experiment 1, 2, and 3.

Table 1

Percentage of data loss in Experiment 1, 2, and 3.

|  | Head | Grammatical match | Ungrammatical match | Grammatical mismatch | Ungrammatical mismatch | Mean |
| --- | --- | --- | --- | --- | --- | --- |
| **Exp. 1** | feminine | 5 | 5.4 | 4.9 | 5.4 | 5.2 |
|  | neuter | 4.9 | 5.7 | 4.3 | 5.2 | 5 |
| **Exp.2** | feminine | 7.8 | 6.8 | 7 | 7.5 | 7.3 |
|  | neuter | 7.4 | 6.8 | 7.1 | 7 | 7.1 |
| **Exp. 3** | feminine (adjectival predicates) | 3.2 | 0.9 | 1.4 | 2.3 | 2 |
|  | neuter (adjectival predicates) | 2.3 | 1.4 | 2.3 | 1.8 | 2 |
|  | feminine (object-clitics) | 1.8 | 1.4 | 3.6 | 1.8 | 2.2 |
|  | neuter (object-clitics) | 3.2 | 4.5 | 2.3 | 3.2 | 3.3 |

# Mean RTs by region by condition in Experiment 1 and Experiment 2 (feminine and neuter heads).

**Table 2**

Mean reaction times by condition and by region in feminine heads and neuter heads in Experiment 1.

| *Feminine heads* | | | | | | | | | |
| --- | --- | --- | --- | --- | --- | --- | --- | --- | --- |
|  | R1 | R2 | R3 | R4 | R5 | R6 | R7 | R8 |  |
| grammatical match | 444 | 460 | 608 | 572 | 573 | 629 | 512 | 766 |  |
| ungrammatical match | 448 | 523 | 629 | 591 | 665 | 684 | 577 | 821 |  |
| grammatical mismatch | 486 | 509 | 641 | 581 | 580 | 603 | 501 | 780 |  |
| ungrammatical mismatch | 426 | 500 | 618 | 568 | 580 | 668 | 496 | 759 |  |
|  |  |  |  |  |  |  |  |  |  |
| *Neuter heads* | | | | | | | | |  |
|  | R1 | R2 | R3 | R4 | R5 | R6 | R7 | R8 |  |
| grammatical match | 447 | 507 | 660 | 640 | 637 | 627 | 476 | 794 |  |
| ungrammatical match | 470 | 465 | 623 | 527 | 555 | 665 | 482 | 770 |  |
| grammatical mismatch | 477 | 526 | 698 | 615 | 605 | 623 | 519 | 730 |  |
| ungrammatical mismatch | 470 | 546 | 663 | 623 | 685 | 731 | 556 | 853 |  |

**Table 3**

Mean reaction times by condition and by region in feminine heads and neuter heads in Experiment 2.

| *Feminine heads* | | | | | | | | | |
| --- | --- | --- | --- | --- | --- | --- | --- | --- | --- |
|  | R1 | R2 | R3 | R4 | R5 | R6 | R7 | R8 |  |
| grammatical match | 446 | 559 | 567 | 537 | 482 | 584 | 440 | 683 |  |
| ungrammatical match | 429 | 580 | 605 | 567 | 497 | 629 | 503 | 704 |  |
| grammatical mismatch | 485 | 580 | 584 | 589 | 486 | 565 | 452 | 667 |  |
| ungrammatical mismatch | 424 | 578 | 607 | 553 | 500 | 579 | 472 | 710 |  |
|  |  |  |  |  |  |  |  |  |  |
|  |  |  |  |  |  |  |  |  |  |
| *Neuter heads* | | | | | | | | |  |
|  | R1 | R2 | R3 | R4 | R5 | R6 | R7 | R8 |  |
| grammatical match | 445 | 595 | 624 | 595 | 555 | 645 | 503 | 709 |  |
| ungrammatical match | 470 | 615 | 616 | 600 | 590 | 685 | 535 | 740 |  |
| grammatical mismatch | 476 | 593 | 615 | 600 | 534 | 620 | 479 | 672 |  |
| ungrammatical mismatch | 472 | 538 | 582 | 551 | 530 | 595 | 459 | 705 |  |

# Adverbs used in Experiment 3 and 4

## Adjectival predicates

εντελώς “completely”, πολύ προσεκτικά “very carefully”, τελείως “completely”, υπερβολικά “extremely”, πολύ καλά “very well”, αρκετά “quite”, πολύ σφιχτά “very tightly”, πολύ ψηλά “very high”, πολύ άσχημα “very bad”, πολύ πρόχειρα “very haphazardly”, πολύ σχολαστικά “very meticulously”, πολύ εντυπωσιακά “very impressively”.

## Object-clitics

ευτυχώς “fortunately”, τελικά “eventually”, πολύ γρήγορα “very fast”, λίγο αργότερα “a bit later”, επιτέλους “at last”, δυστυχώς “unfortunately”, πριν λίγο “shortly before”, λίγο νωρίτερα “a bit earlier”, μετά από λίγο “after a while”, ύστερα από λίγο “after a while”, εν τέλει “finally”, αργότερα “later”.

# Mean accuracy score in the comprehension questions of Experiment 1 and 2 followed by the corresponding linear logistic regression model.

**Table 4**

Participants’ mean accuracy and standard error to comprehension questions in feminine and neuter heads in Experiment 1 and 2.

|  | **Grammatical match** | **Ungrammatical match** | | **Grammatical mismatch** | | **Ungrammatical mismatch** | |  |  |
| --- | --- | --- | --- | --- | --- | --- | --- | --- | --- |
| **Experiment 1** | | | | | | | | |  |
| feminine heads | 96 (1.09) | | 97 (0.9) | | 97 (1.0) | | 96 (1.13) | | |
| neuter heads | 98 (0.92) | | 97 (1.3) | | 99 (0.60) | | 97 (1.09) | | |
| **Experiment 2** | | | | | | | | |  |
| feminine heads | 96 (1.11) | | 98 (0.89) | | 96 (1.16) | | 95 (1.24) | | |
| neuter heads | 98 (0.87) | | 96 (1.11) | | 96 (1.16) | | 96 (1.11) | | |

**Table 5**

Linear mixed-effects logistic regression model results of participants’ accuracy in Experiment 1 and 2.

|  | **Feminine heads** | | | | **Neuter heads** | | | |
| --- | --- | --- | --- | --- | --- | --- | --- | --- |
| **Experiment 1** | β | SE | z | p | β | SE | z | p |
| Grammaticality | 1.989 | 1.337 | 1.49 | .137 | -0.365 | 0.652 | -0.97 | **.**330 |
| Attractor | -1.314 | 1.303 | -1.01 | .313 | -0.020 | 0.922 | -0.02 | .982 |
| Grammaticality:Attractor | -4.724 | 2.663 | 1.79 | .073 | -1.136 | 1.393 | -0.82 | .415 |
|  |  | | | |  | | | |
| **Experiment 2** |  |  |  |  |  |  |  |  |
| Grammaticality | 1.165 | 1.612 | 0.72 | .470 | -0.233 | 0.363 | -0.64 | .521 |
| Attractor | -1.694 | 1.650 | -1.03 | .305 | -0.329 | 0.367 | -0.90 | .370 |
| Grammaticality:Attractor | -3.322 | 3.096 | -1.07 | .283 | 0.759 | 0.743 | 1.02 | .307 |

# Linear mixed-effects model results for Region 3 (head) and 8 (sentence-final region) in Experiment 1 and 2.

**Table 6**

Linear mixed-effects model results for Regions 3 and 8 with feminine and neuter heads in Experiment 1 and 2.

|  | **Region 3 (head)** | | | | | **Region 8 (sentence-final)** | | | | | | | |
| --- | --- | --- | --- | --- | --- | --- | --- | --- | --- | --- | --- | --- | --- |
| **Feminine Heads - Experiment 1** | | | | | | | | | | | | |  |
|  | | β | SE | t | p | | β | SE | t | | p | |  |
| Grammaticality | | -0.006 | 0.031 | -0.21 | .837 | | -0.007 | 0.049 | -0.137 | | .892 | |  |
| Attractor | | 0.008 | 0.027 | 0.29 | .773 | | -0.014 | 0.045 | -0.319 | | .752 | |  |
| Grammaticality:Attractor | | -0.060 | 0.078 | -0.77 | .448 | | -0.104 | 0.108 | -0.965 | | .343 | |  |
| **Neuter Heads - Experiment 1** | | | | | | | | | | | |  |  |
|  | | β | SE | t | p | | β | SE | t | | p | |  |
| Grammaticality | | -0.044 | 0.033 | -1.32 | .203 | | 0.017 | 0.048 | 0.35 | | .729 | |  |
| Attractor | | **0.080** | **0.032** | **2.53** | **.016** | | 0.004 | 0.045 | 0.10 | | .925 | |  |
| Grammaticality:Attractor | | 0.041 | 0.069 | 0.59 | .560 | | 0.167 | 0.092 | 1.82 | | .083 | |  |
| **Feminine heads - Experiment 2** | | | | | | | | | |  |  |  |  |
|  | | β | SE | t | p | | β | SE | t | | p | |  |
| Grammaticality | | 0.033 | 0.039 | 0.83 | .414 | | 0.035 | 0.033 | 1.05 | | .305 | |  |
| Attractor | | 0.004 | 0.037 | 0.10 | .919 | | -0.020 | 0.026 | -0.76 | | .447 | |  |
| Grammaticality:Attractor | | -0.018 | 0.053 | -0.33 | .743 | | 0.021 | 0.054 | 0.38 | | .704 | |  |
| **Neuter heads - Experiment 2** | | | | | | | | | | | |  |  |
|  | | β | SE | t | p | | β | SE | t | | p | |  |
| Grammaticality | | -0.023 | 0.034 | -0.68 | .502 | | 0.018 | 0.047 | 0.39 | | .703 | |  |
| Attractor | | -0.050 | 0.027 | -1.86 | .078 | | -0.066 | 0.047 | -1.41 | | .171 | |  |
| Grammaticality:Attractor | | -0.062 | 0.060 | -1.05 | .308 | | -0.014 | 0.055 | -0.26 | | .796 | |  |

# Linear mixed-effects model results of the untrimmed data on Region 5 (critical region/agreement target), 6 (post-critical 1), and 7 (post-critical 2).

**Table 7**

Linear mixed-effects model results on the untrimmed data in Experiment 1with feminine and neuter heads for Region 4 (attractor region), Region 5 (agreement target/region 5), Region 6 (post-critical 1), and Region 7 (post-critical 2).

| **Feminine Heads** | | | | | | | | | | | | |
| --- | --- | --- | --- | --- | --- | --- | --- | --- | --- | --- | --- | --- |
|  | Region 3 (Head) | | | | | Region 4 (Attractor region) | | | | | | |
|  | | β | SE | t | p | | | β | SE | t | p | |
| Grammaticality | | -3.34 | 25.77 | -0.13 | .244 | | | 20.04 | 20.11 | 0.997 | .319 | |
| Attractor | | -13.91 | 26.39 | -0.527 | .783 | | | 2.398 | 20.11 | 0.119 | .905 | |
| Grammaticality:Attractor | | -70.79 | 51.09 | -1.386 | .062 | | | -19.49 | 40.21 | -0.845 | .628 | |
| *Attraction model* | | -52.60 | 28.66 | -1.835 | .136 | | | -5.892 | 26.64 | -0.199 | .843 | |
|  | Region 5 (Agreement target) | | | | | | Region 6 (post-critical 1) | | | | |  |
|  | | β | SE | t | p | | | β | SE | t | p | |
| Grammaticality | | **60.68** | **47.** | **3.03** | **.003** | | | **58.929** | **21.88** | **2.694** | **.008** | |
| Attractor | | **-60.73** | **20.02** | **-3.03** | **.003** | | | -23.62 | 22.5 | -1.050 | .297 | |
| Grammaticality:Attractor | | **-95.59** | **40.11** | **-2.38** | **.017** | | | -3.893 | 41.35 | -0.094 | .925 | |
| *Attraction model* | | **-109.5** | **31.83** | **-2.38** | **.001** | | | -26.83 | 29.77 | -0.901 | .368 | |
|  | Region 7 (post-critical 2) | | | | | Region 8 (sentence-final region) | | | | | | |
|  | | β | SE | t | p | | | β | SE | t | p | |
| Grammaticality | | 35.36 | 28.21 | 0.696 | .491 | | | 41.32 | 30.31 | 1.36 | .173 | |
| Attractor | | 50.82 | 26.81 | -1.76 | .089 | | | -37.15 | 30.34 | -1.22 | .221 | |
| Grammaticality:Attractor | | 36.12 | 47.24 | -0.74 | .464 | | | -107.8 | 60.66 | -1.78 | .076 | |
| *Attraction model* | | **-67.97** | **27.11** | **-2.51** | **.015** | | | **-96.87** | **48.58** | **-1.99** | **.047** | |
| **Neuter Heads** | | | | | | | | | | | | |
|  | Region 3 (Attractor) | | | | | Region 4 (Agreement target) | | | | | | |
|  | | β | SE | t | p | | | β | SE | t | p | |
| Grammaticality | | -15.52 | 22.45 | -0.691 | .490 | | | **-50.77** | **21.66** | **-2.344** | **.019** | |
| Attractor | | 23.91 | 22.47 | 1.064 | .288 | | | -11.34 | 23.74 | -0.748 | .634 | |
| Grammaticality:Attractor | | 14.40 | 44.93 | 0.320 | .749 | | | 134.32 | 44.12 | 3.044 | .003 | |
| *Attraction model* | | 29.36 | 33.32 | 33.32 | .379 | | | 54.85 | 27.47 | 1.997 | .051 | |
|  | Region 5 (post-critical 1) | | | | | | Region 6 (post-critical 2) | | | | |  |
|  | | β | SE | t | p | | | β | SE | t | p | |
| Grammaticality | | 15.12 | 29.52 | 0.51 | .613 | | | **94.50** | **18.71** | **5.05** | **<.001** | |
| Attractor | | 25.26 | 31.59 | 0.80 | .432 | | | 23.09 | 18.72 | 1.23 | .218 | |
| Grammaticality:Attractor | | 117.68 | 61.0 | 1.93 | .067 | | | 20.21 | 34.43 | 0.54 | .589 | |
| *Attraction model* | | **86.93** | **27.93** | **3.11** | **.002** | | | 29.47 | 53.29 | 0.53 | .586 | |
|  | Region 7 (Attractor) | | | | | Region 8 (Agreement target) | | | | | | |
|  | | β | SE | t | p | | | β | SE | t | p | |
| Grammaticality | | 35.36 | 25.41 | 1.39 | .177 | | | 33.249 | 28.53 | 1.165 | .244 | |
| Attractor | | **50.82** | **22.91** | **2.22** | **.031** | | | 7.889 | 28.56 | 0.276 | .783 | |
| Grammaticality:Attractor | | 36.12 | 40.03 | .902 | .370 | | | 106.73 | 57.01 | 1.87 | .062 | |
| *Attraction model* | | **68.01** | **26.72** | **2.55** | **.011** | | | 59.260 | 39.67 | 1.494 | .136 | |

**Table 8**

Linear mixed-effects model results on the untrimmed data in Experiment 2 with feminine and neuter heads for Region 4 (attractor region), Region 5 (agreement target/region 5), Region 6 (post-critical 1), and Region 7 (post-critical 2).

| **Feminine Heads** | | | | | | | | | | | | |
| --- | --- | --- | --- | --- | --- | --- | --- | --- | --- | --- | --- | --- |
|  | Region 3 (Head) | | | | | Region 4 (Attractor region) | | | | | | |
|  | | β | SE | t | p | | | β | SE | t | p | |
| Grammaticality | | 38.19 | 26.18 | 1.459 | .151 | | | -4.547 | 24.03 | -0.189 | .851 | |
| Attractor | | 23.65 | 33.35 | 0.709 | .482 | | | 26.391 | 23.84 | 1.107 | .277 | |
| Grammaticality:Attractor | | -17.24 | 47.75 | -0.361 | .718 | | | -61.38 | 47.16 | -1.302 | .205 | |
| *Attraction model* | | -4.938 | 22.46 | -0.220 | .826 | | | -4.078 | 26.84 | -0.152 | .879 | |
|  | Region 5 (Agreement target) | | | | | | Region 6 (post-critical 1) | | | | |  |
|  | | β | SE | t | p | | | β | SE | t | p | |
| Grammaticality | | -24.93 | 20.09 | 1.24 | .215 | | | 38.130 | 23.74 | 1.606 | .120 | |
| Attractor | | -1.353 | 20.10 | -0.067 | .946 | | | **-40.62** | **20.05** | **-2.026** | **.048** | |
| Grammaticality:Attractor | | -28.36 | 40.18 | -0.706 | .480 | | | -51.60 | 34.13 | -1.512 | .134 | |
| *Attraction model* | | -13.16 | 31.34 | -0.420 | .676 | | | **-67.81** | **24.60** | **-2.757** | **.006** | |
|  | Region 7 (post-critical 2) | | | | | Region 8 (sentence-final region) | | | | | | |
|  | | β | SE | t | p | | | β | SE | t | p | |
| Grammaticality | | **43.144** | **15.47** | **2.79** | **.005** | | | 32.90 | 25.61 | 1.285 | .199 | |
| Attractor | | -5.127 | 15.49 | -0.33 | .741 | | | -27.56 | 25.64 | -1.08 | .283 | |
| Grammaticality:Attractor | | -50.71 | 30.95 | -1.64 | .102 | | | 51.25 | 51.20 | 1.001 | .317 | |
| *Attraction model* | | -31.33 | 23.90 | -1.31 | .190 | | | -30.11 | 32.30 | -0.93 | .356 | |
| **Neuter Heads** | | | | | | | | | | | | |
|  | Region 3 (Attractor) | | | | | Region 4 (Agreement target) | | | | | | |
|  | | β | SE | t | p | | | β | SE | t | p | |
| Grammaticality | | -32.97 | 20.67 | -1.595 | .111 | | | -30.92 | 21.64 | -1.429 | .157 | |
| Attractor | | -19.43 | 20.66 | -0.941 | .347 | | | -7.188 | 21.25 | -0.338 | .736 | |
| Grammaticality:Attractor | | -22.25 | 41.32 | -0.539 | .590 | | | -38.12 | 40.53 | -0.941 | .349 | |
| *Attraction model* | | -4.938 | 22.46 | -0.220 | .826 | | | -25.83 | 25.94 | -0.996 | .320 | |
|  | Region 5 (post-critical 1) | | | | | | Region 6 (post-critical 2) | | | | |  |
|  | | β | SE | t | p | | | β | SE | t | p | |
| Grammaticality | | 21.930 | 35.64 | 0.615 | .542 | | | 18.790 | 31.54 | 0.596 | .556 | |
| Attractor | | -16.63 | 35.49 | -0.469 | .642 | | | -42.31 | 29.58 | -1.431 | .162 | |
| Grammaticality:Attractor | | -87.31 | 59.59 | -1.465 | .149 | | | -71.53 | 54.77 | -1.306 | .200 | |
| *Attraction model* | | -60.45 | 35.34 | -1.710 | .088 | | | **-0.079** | **0.035** | **-2.238** | **.030** | |
|  | Region 7 (Attractor) | | | | | Region 8 (Agreement target) | | | | | | |
|  | | β | SE | t | p | | | β | SE | t | p | |
| Grammaticality | | 5.302 | 27.58 | 0.192 | .849 | | | 22.38 | 25.85 | 0.865 | .387 | |
| Attractor | | -32.29 | 24.45 | -1.32 | .200 | | | -29.28 | 25.83 | -1.13 | .257 | |
| Grammaticality:Attractor | | -9.612 | 47.05 | -0.21 | .840 | | | -70.24 | 51.62 | -1.36 | .174 | |
| *Attraction model* | | -41.27 | 23.31 | -1.77 | .077 | | | -30.11 | 32.30 | -0.93 | .356 | |

# Linear mixed-effects model results of the z-transformed ratings in Experiment 4.

**Table 9**

Linear mixed-effects model results of participants’ z-scored ratings in adjectival predicates and object-clitics in Experiment 4.

|  | **Feminine heads** | | | | **Neuter heads** | | | | | | | | | | |
| --- | --- | --- | --- | --- | --- | --- | --- | --- | --- | --- | --- | --- | --- | --- | --- |
| **Adjectival predicates** | β | SE | z | p | β | | SE | | z | | p | |  |  |  |
| Grammaticality | **1.279** | **0.131** | **9.74** | **<0.001** | | **1.237** | | **0.111** | | **11.15** | | **<0.001** | | |  |
| Attractor | -0.032 | 0.043 | -0.76 | 0.454 | | 0.009 | | 0.041 | | 0.212 | | 0.832 | | |  |
| Grammaticality:Attractor | 0.010 | 0.096 | 0.10 | 0.920 | | 0.100 | | 0.096 | | 1.04 | | 0.315 | | |  |
|  | **Feminine heads** | | | | **Neuter heads** | | | | | | | | |  |  |
| **Object-clitics** | Β | SE | z | p | β | | SE | | z | | p | |  |  |  |
| Grammaticality | **1.260** | **0.143** | **8.79** | **<0.001** | | **1.244** | | **0.137** | | **9.103** | | **<0.001** | | |  |
| Attractor | -0.068 | 0.045 | -1.54 | 0.140 | | **-0.108** | | **0.050** | | **-2.17** | | **0.040** | | |  |
| Grammaticality:Attractor | 0.173 | 0.088 | 1.97 | 0.061 | | 0.100 | | 0.101 | | 0.98 | | 0.356 | | |  |

**Adjectival predicates**

**Feminine heads:** The model showed a main effect of Grammaticality such that grammatical sentences received higher acceptability rating than ungrammatical sentences. The effect of Attractor and the interaction between Grammaticality and Attractor were not significant. The pairwise comparisons did not show any differences either (*p >*.05).

**Neuter heads:** The model revealed a main effect of Grammaticality such that grammatical sentences received higher ratings than ungrammatical sentences. No significant interaction between Grammaticality and Attractor was found. The pairwise comparisons did not show any significant differences either (*p >.*05).

**Object-clitics**

**Feminine heads:** The model revealed a main effect of Grammaticality such that grammatical sentences received higher acceptability ratings than ungrammatical sentences. The interaction between Grammaticality and Attractor was marginally significant reflecting a tendency towards higher acceptability ratings for the ungrammatical mismatch condition compared to the ungrammatical match condition. Pairwise comparisons confirmed this tendency, given that the ungrammatical sentences seemed to be modulated by attractor mismatch with higher ratings for the ungrammatical mismatch condition (*β* = -0.151; *SE* = 0.049; *t* = -3.085; *p* = .002), compared to the ungrammatical match condition, while grammatical sentences were not modulated by attractor mismatch (*β* = 0.018; *SE* = 0.054; *t* = 0.329; *p* = .748), indicating an asymmetrical pattern of attraction. The model of Harmony in ungrammatical sentences revealed a main effect of Attractor (*β* = -0.144; *SE* = 0.050; *z* = -2.89; *p* = .006), confirming the existence of attraction. However, no effect of Harmony was detected (*β* = -0.0001; *SE* = 0.064; *z* = -0.002; *p* = .999) and no interaction with Attractor (*β* = -0.025; *SE* = 0.107; *z* = -0.237; *p* = .813).

**Neuter heads:** The model revealed a main effect of Grammaticality such that grammatical sentences received higher acceptability ratings than ungrammatical sentences and a main effect of Attractor such that mismatch sentences received higher acceptability ratings than match sentences. The interaction did not reach significance. However, the pairwise comparisons confirmed an asymmetrical pattern with attraction occurring between the ungrammatical sentences and not between the grammatical ones: attractor mismatch increased acceptability ratings in the ungrammatical sentences (*β* = -0.156; *SE* = 0.059; *t* = -2.634; p = .009, but did not decrease ratings in the grammatical sentences: *β* = -0.058; *SE* = 0.058; *t* = -0.99; *p* = .331). The model of Harmony in ungrammatical sentences revealed a main effect of Attractor (*β* = -0.155; *SE* = 0.059; *z* = -2.626; *p* = .009), confirming the existence of attraction. However, no effect of Harmony was detected (*β* = -0.174; *SE* = 0.121; *z* = -1.44; *p* = .163) and no interaction with Attractor (*β* = -0.069; *SE* = 0.122; *z* = -0.57; *p* = .573).

# Wafeform of Experiment 2 on the critical region (2), agreement target: object clitic

| 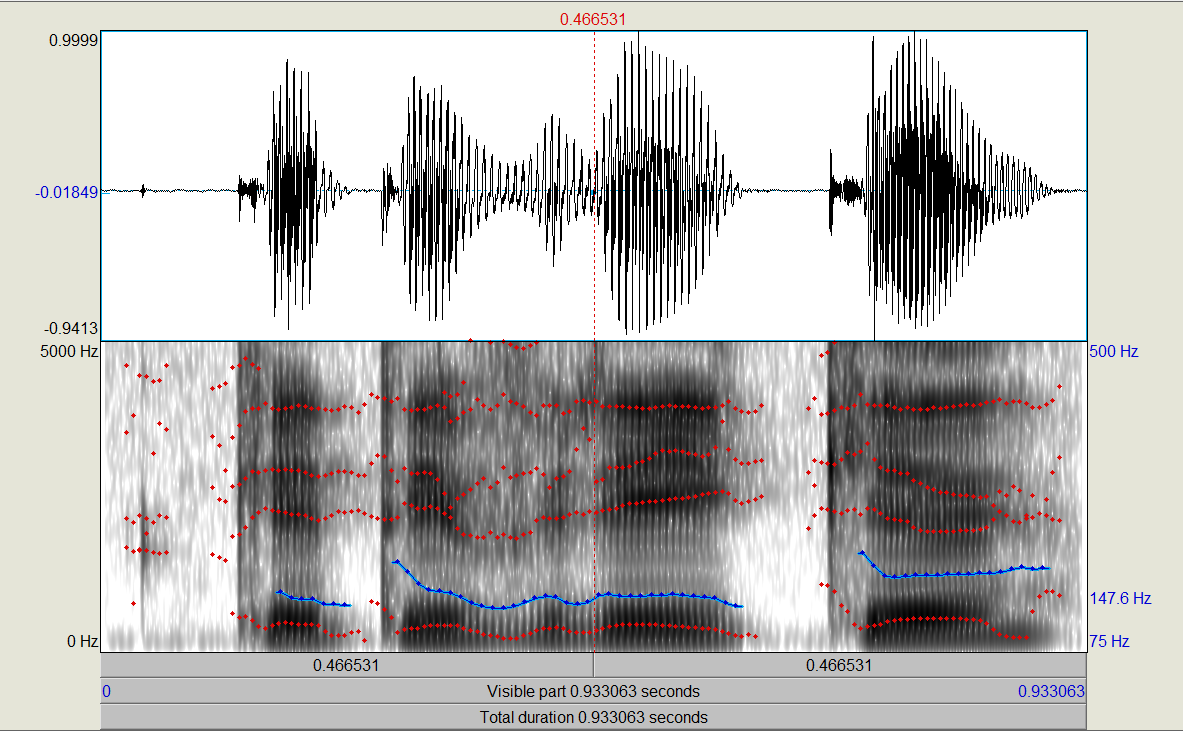 |
| --- |
| Ke (and) to (it) vri- ke (found) |

Figure 1. Waveform of the critical region with the object-clitic as the agreement target.

# Materials of Study 1 (same materials were used in Study 2).

Heads are in bold, Attractors are in italics, agreement targets are in bold and italics. Conditions: a.: attractor match, grammatical, b.: attractor match, ungrammatical, c.: attractor mismatch, grammatical, d.: attractor mismatch, ungrammatical

Item 1a

a. Όταν ο Πέτρος τελείωσε τις δουλειές του σπιτιού βρήκε το χαλί για το πάτωμα βρεγμένο δίπλα στο λάστιχο του κήπου.

b. Όταν ο Πέτρος τελείωσε τις δουλειές του σπιτιού βρήκε το χαλί για το πάτωμα βρεγμένη δίπλα στο λάστιχο του κήπου.

c. Όταν ο Πέτρος τελείωσε τις δουλειές του σπιτιού βρήκε το χαλί για την κουζίνα βρεγμένο δίπλα στο λάστιχο του κήπου.

d. Όταν ο Πέτρος τελείωσε τις δουλειές του σπιτιού βρήκε το χαλί για την κουζίνα βρεγμένη δίπλα στο λάστιχο του κήπου.

“When Peter finished the housework, he found the carpet for the floor/kitchen wet near the hose of the garden”.

Item 2a

a. Ο μάγειρας που διαφήμιζε στον κόσμο τις νέες συνταγές του είχε το φαγητό το φαγητό για το μαγαζί σκεπασμένο μέσα στο ταψί του βοηθού του.

b. Ο μάγειρας που διαφήμιζε στον κόσμο τις νέες συνταγές του είχε το φαγητό το φαγητό για το μαγαζί σκεπασμένη μέσα στο ταψί του βοηθού του.

c. Ο μάγειρας που διαφήμιζε στον κόσμο τις νέες συνταγές του είχε το φαγητό το φαγητό για την έκθεση σκεπασμένο μέσα στο ταψί του βοηθού του.

d. Ο μάγειρας που διαφήμιζε στον κόσμο τις νέες συνταγές του είχε το φαγητό το φαγητό για την έκθεση σκεπασμένη μέσα στο ταψί του βοηθού του.

“The cook, who was advertising his new recipes to the crowd, had the food for the store/exhibition covered in his assistant’s baking tray”.

Item 3a

a. Όταν ο Κώστας μπήκε στην κουζίνα είδε το αυγό για το γλυκό σπασμένο δίπλα στο κουτί με τις σοκολάτες.

b. Όταν ο Κώστας μπήκε στην κουζίνα είδε το αυγό για το γλυκό σπασμένη δίπλα στο κουτί με τις σοκολάτες.

c. Όταν ο Κώστας μπήκε στην κουζίνα είδε το αυγό για τη σούπα σπασμένο δίπλα στο κουτί με τις σοκολάτες.

d. Όταν ο Κώστας μπήκε στην κουζίνα είδε το αυγό για τη σούπα σπασμένη δίπλα στο κουτί με τις σοκολάτες.

“When Kostas went into the kitchen, he found the egg for the dessert/soup broken next to the box with the chocolates”.

Item 4a

a. Ο αργοπορημένος επιβάτης είδε το εισιτήριο για το ταξίδι σκισμένο μέσα στην τσάντα της γυναίκας του.

b. Ο αργοπορημένος επιβάτης είδε το εισιτήριο για το ταξίδι σκισμένη μέσα στην τσάντα της γυναίκας του.

c. Ο αργοπορημένος επιβάτης είδε το εισιτήριο για την εκδρομή σκισμένο μέσα στην τσάντα της γυναίκας του.

d. Ο αργοπορημένος επιβάτης είδε το εισιτήριο για την εκδρομή σκισμένη μέσα στην τσάντα της γυναίκας του.

“The late passenger saw the ticket for the trip/excursion torn in his wife’s bag”.

Item 5a

a. Καθώς ο Πέτρος έστρωνε το τραπέζι βρήκε το κουτάλι για το γλυκό λερωμένο πάνω στον πάγκο της κουζίνας.

b. Καθώς ο Πέτρος έστρωνε το τραπέζι βρήκε το κουτάλι για το γλυκό λερωμένη πάνω στον πάγκο της κουζίνας.

c. Καθώς ο Πέτρος έστρωνε το τραπέζι βρήκε το κουτάλι για τη σούπα λερωμένο πάνω στον πάγκο της κουζίνας.

d. Καθώς ο Πέτρος έστρωνε το τραπέζι βρήκε το κουτάλι για τη σούπα λερωμένη πάνω στον πάγκο της κουζίνας.

“While Kostas was setting the table, he found the spoon for the dessert/soup stained on the kitchen counter.”

Item 6a

a. Καθώς ο Κώστας ετοιμαζόταν να φύγει, είδε το κλειδί για το αμάξι κλεισμένο μέσα στο συρτάρι του κομοδίνου.

b. Καθώς ο Κώστας ετοιμαζόταν να φύγει, είδε το κλειδί για το αμάξι κλεισμένη μέσα στο συρτάρι του κομοδίνου.

c. Καθώς ο Κώστας ετοιμαζόταν να φύγει, είδε το κλειδί για τη μηχανή κλεισμένο μέσα στο συρτάρι του κομοδίνου.

d. Καθώς ο Κώστας ετοιμαζόταν να φύγει, είδε το κλειδί για τη μηχανή κλεισμένη μέσα στο συρτάρι του κομοδίνου.

“When Kostas was about to leave, he saw the key for the car/motorbike closed in the drawer of the bedside table.”

Item 7a

a. O Βασίλης που φοβόταν πολύ το σκοτάδι, βρήκε το φανάρι για το πατάρι χαλασμένο δίπλα στις λαμπάδες της Ανάστασης.

b. O Βασίλης που φοβόταν πολύ το σκοτάδι, βρήκε το φανάρι για το πατάρι χαλασμένη δίπλα στις λαμπάδες της Ανάστασης.

c. O Βασίλης που φοβόταν πολύ το σκοτάδι, βρήκε το φανάρι για την εξοχή χαλασμένο δίπλα στις λαμπάδες της Ανάστασης.

d. O Βασίλης που φοβόταν πολύ το σκοτάδι, βρήκε το φανάρι για την εξοχή χαλασμένη δίπλα στις λαμπάδες της Ανάστασης.

“Vasilis, who was afraid of the dark, found the lantern for the attic/countryside next to the lampades (candles of the Resurrection).”

Item 8a

a. Όταν ο Πέτρος έφτασε στο ακριβό ξενοδοχείο που θα απολάμβανε τις διακοπές του, βρήκε το μπουρνούζι για το δωμάτιο πλυμένο δίπλα στο γραφείο της ρεσεψιόν.

b. Όταν ο Πέτρος έφτασε στο ακριβό ξενοδοχείο που θα απολάμβανε τις διακοπές του, βρήκε το μπουρνούζι για το δωμάτιο πλυμένη δίπλα στο γραφείο της ρεσεψιόν.

c. Όταν ο Πέτρος έφτασε στο ακριβό ξενοδοχείο που θα απολάμβανε τις διακοπές του, βρήκε το μπουρνούζι για την πισίνα πλυμένο δίπλα στο γραφείο της ρεσεψιόν.

d. Όταν ο Πέτρος έφτασε στο ακριβό ξενοδοχείο που θα απολάμβανε τις διακοπές του, βρήκε το μπουρνούζι για την πισίνα πλυμένη δίπλα στο γραφείο της ρεσεψιόν.

“When Petros arrived at the expensive hotel where he would enjoy his vacation, his found the bathrobe for the room/pool washed near the reception desk”.

Item 9a

a. Ο Δημήτρης επέστρεψε στο σπίτι, είδε το φωτιστικό για το δάπεδο στολισμένο μέσα στο κουτί της μετακόμισης.

b. Ο Δημήτρης επέστρεψε στο σπίτι, είδε το φωτιστικό για το δάπεδο στολισμένη μέσα στο κουτί της μετακόμισης.

c. Ο Δημήτρης επέστρεψε στο σπίτι, είδε το φωτιστικό για την ταράτσα στολισμένο μέσα στο κουτί της μετακόμισης.

d. Ο Δημήτρης επέστρεψε στο σπίτι, είδε το φωτιστικό για την ταράτσα στολισμένη μέσα στο κουτί της μετακόμισης.

“When Dimitris went back home, he saw the lamp for the floor/terrace decorated in the box of the move.”

Item 10a

a. Ο Μάνος που έδωσε εντολή να τακτοποιηθούν όλα τα πράγματα, είδε το κιβώτιο για το πατάρι λασπωμένο πάνω στον καναπέ του σαλονιού.

b. Ο Μάνος που έδωσε εντολή να τακτοποιηθούν όλα τα πράγματα, είδε το κιβώτιο για το πατάρι λασπωμένη πάνω στον καναπέ του σαλονιού.

c. Ο Μάνος που έδωσε εντολή να τακτοποιηθούν όλα τα πράγματα, είδε το κιβώτιο για την πιλοτή λασπωμένο πάνω στον καναπέ του σαλονιού.

d. Ο Μάνος που έδωσε εντολή να τακτοποιηθούν όλα τα πράγματα, είδε το κιβώτιο για την πιλοτή λασπωμένη πάνω στον καναπέ του σαλονιού.

“Manos who ordered all things be arranged, saw the box for the attic/pilotis muddy on the living room sofa.”

Item 11a

a. Ο Πέτρος που ήθελε να διαβάσει περισσότερες πληροφορίες για τον τόπο που μεγάλωσε, βρήκε το περιοδικό για το νησί πεταμένο μέσα στον κάδο απορριμμάτων της πολυκατοικίας.

b. Ο Πέτρος που ήθελε να διαβάσει περισσότερες πληροφορίες για τον τόπο που μεγάλωσε, βρήκε το περιοδικό για το νησί πεταμένη μέσα στον κάδο απορριμμάτων της πολυκατοικίας.

c. Ο Πέτρος που ήθελε να διαβάσει περισσότερες πληροφορίες για τον τόπο που μεγάλωσε, βρήκε το περιοδικό για την πόλη πεταμένο μέσα στον κάδο απορριμμάτων της πολυκατοικίας.

d. Ο Πέτρος που ήθελε να διαβάσει περισσότερες πληροφορίες για τον τόπο που μεγάλωσε, βρήκε το περιοδικό για την πόλη πεταμένη μέσα στον κάδο απορριμμάτων της πολυκατοικίας.

“Petros who wanted to read more about the place he grew up, found the magazine for the island/town thrown into the garbage bin of the block of flats.”

Item 12a

a. Καθώς ο Μάνος περίμενε τον ηλεκτρολόγο, είχε το καλώδιο για το υπόγειο κρεμασμένο πάνω στον καλόγερο της εισόδου.

b. Καθώς ο Μάνος περίμενε τον ηλεκτρολόγο, είχε το καλώδιο για το υπόγειο κρεμασμένη πάνω στον καλόγερο της εισόδου.

c. Καθώς ο Μάνος περίμενε τον ηλεκτρολόγο, είχε το καλώδιο για την ταράτσα κρεμασμένο πάνω στον καλόγερο της εισόδου.

d. Καθώς ο Μάνος περίμενε τον ηλεκτρολόγο, είχε το καλώδιο για την ταράτσα κρεμασμένη πάνω στον καλόγερο της εισόδου.

“While Manos was waiting for the electrician, had the cable for the basement/terrace hung on coat hanger of the entrance.”

Item 13a

a. Ο ιδιοκτήτης της επιχείρησης που ήταν απατεώνας, είχε το εμπόρευμα για το μαγαζί ληγμένο πάνω στο καρότσι του σούπερ-μάρκετ.

b. Ο ιδιοκτήτης της επιχείρησης που ήταν απατεώνας, είχε το εμπόρευμα για το μαγαζί ληγμένη πάνω στο καρότσι του σούπερ-μάρκετ.

c. Ο ιδιοκτήτης της επιχείρησης που ήταν απατεώνας, είχε το εμπόρευμα για την έκθεση ληγμένο πάνω στο καρότσι του σούπερ-μάρκετ.

d. Ο ιδιοκτήτης της επιχείρησης που ήταν απατεώνας, είχε το εμπόρευμα για την έκθεση ληγμένη πάνω στο καρότσι του σούπερ-μάρκετ.

“The owner of the company who was a scammer, had the commodity for the store/exhibition expired on the supermarket stroller.”

Item 14a

a. Ο μάγειρας που προσπαθούσε να εκπαιδεύσει τους τεμπέληδες βοηθούς του, είδε το ράφι για το γάλα σκονισμένο δίπλα στον φούρνο μικροκυμάτων.

b. Ο μάγειρας που προσπαθούσε να εκπαιδεύσει τους τεμπέληδες βοηθούς του, είδε το ράφι για το γάλα σκονισμένη δίπλα στον φούρνο μικροκυμάτων.

c. Ο μάγειρας που προσπαθούσε να εκπαιδεύσει τους τεμπέληδες βοηθούς του, είδε το ράφι για την κρέμα σκονισμένο δίπλα στον φούρνο μικροκυμάτων.

d. Ο μάγειρας που προσπαθούσε να εκπαιδεύσει τους τεμπέληδες βοηθούς του, είδε το ράφι για την κρέμα σκονισμένη δίπλα στον φούρνο μικροκυμάτων.

“The cook, who was trying to train his lazy assistants, saw the shelf for the milk dusty next to the microwave.”

Item 15a

a. Καθώς ο Παύλος ετοίμαζε το βραδινό του, είχε το μέλι για το γάλα ανοιγμένο δίπλα στο κέικ σοκολάτας.

b. Καθώς ο Παύλος ετοίμαζε το βραδινό του, είχε το μέλι για το γάλα ανοιγμένη δίπλα στο κέικ σοκολάτας.

c. Καθώς ο Παύλος ετοίμαζε το βραδινό του, είχε το μέλι για την κρέμα ανοιγμένο δίπλα στο κέικ σοκολάτας.

d. Καθώς ο Παύλος ετοίμαζε το βραδινό του, είχε το μέλι για την κρέμα ανοιγμένη δίπλα στο κέικ σοκολάτας.

“While Paul was prepairing his dinner, he had the honey for the milk/cream opened next to the chocolate cake.”

Item 16a

a. Ο Αλέξης που περίμενε περισσότερα υλικά για την επιδιόρθωση της ζημιάς. είδε το πλακάκι για το πάτωμα ραγισμένο δίπλα στο κέικ σοκολάτας.

b. Ο Αλέξης που περίμενε περισσότερα υλικά για την επιδιόρθωση της ζημιάς. είδε το πλακάκι για το πάτωμα ραγισμένη δίπλα στο κέικ σοκολάτας.

c. Ο Αλέξης που περίμενε περισσότερα υλικά για την επιδιόρθωση της ζημιάς. είδε το πλακάκι για την πισίνα ραγισμένο δίπλα στο κέικ σοκολάτας.

d. Ο Αλέξης που περίμενε περισσότερα υλικά για την επιδιόρθωση της ζημιάς. είδε το πλακάκι για την πισίνα ραγισμένη δίπλα στο κέικ σοκολάτας.

“Alexis, who was waiting for more materials to repair the damage, saw the tile for the floor/swimming pool cracked next to the chocolate cake.”

Item 17a

a. Όταν ο Βασίλης μπήκε στο σαλόνι, βρήκε το πρόστιμο για το αμάξι πεσμένο δίπλα στο τραπεζάκι της τηλεόρασης.

b. Όταν ο Βασίλης μπήκε στο σαλόνι, βρήκε το πρόστιμο για το αμάξι πεσμένη δίπλα στο τραπεζάκι της τηλεόρασης.

c. Όταν ο Βασίλης μπήκε στο σαλόνι, βρήκε το πρόστιμο για τη μηχανή πεσμένο δίπλα στο τραπεζάκι της τηλεόρασης.

d. Όταν ο Βασίλης μπήκε στο σαλόνι, βρήκε το πρόστιμο για τη μηχανή πεσμένη δίπλα στο τραπεζάκι της τηλεόρασης.

“When Vasilis went into the living room, he found the fine for the car/motorbike dropped next to the television table.”

Item 18a

a. O Βασίλης που είχε αρχίσει να πακετάρει, είχε το ρολόι για το δωμάτιο καρφωμένο πάνω στον τοίχο του σαλονιού.

b. O Βασίλης που είχε αρχίσει να πακετάρει, είχε το ρολόι για το δωμάτιο καρφωμένη πάνω στον τοίχο του σαλονιού.

c. O Βασίλης που είχε αρχίσει να πακετάρει, είχε το ρολόι για την κουζίνα καρφωμένο πάνω στον τοίχο του σαλονιού.

d. O Βασίλης που είχε αρχίσει να πακετάρει, είχε το ρολόι για την κουζίνα καρφωμένη πάνω στον τοίχο του σαλονιού.

“Vasilis, who had begun packing, had the clock for the room/kitchen nailed on the living room wall.”

Item 19a

a. O έμπειρος ορειβάτης που σκαρφάλωνε στα πιο ψηλά βουνά, είχε το σχοινί για το ταξίδι δεμένο πάνω στο σακίδιο της γυναίκας του.

b. O έμπειρος ορειβάτης που σκαρφάλωνε στα πιο ψηλά βουνά, είχε το σχοινί για το ταξίδι δεμένη πάνω στο σακίδιο της γυναίκας του.

c. O έμπειρος ορειβάτης που σκαρφάλωνε στα πιο ψηλά βουνά, είχε το σχοινί για την εκδρομή δεμένο πάνω στο σακίδιο της γυναίκας του.

d. O έμπειρος ορειβάτης που σκαρφάλωνε στα πιο ψηλά βουνά, είχε το σχοινί για την εκδρομή δεμένη πάνω στο σακίδιο της γυναίκας του.

“The experienced climber climbing on the highest mountains had the rope for the trip/excursion tied up on his wife's backpack.”

Item 20a

a. Όταν ο Σπύρος έφτιαχνε τη διακόσμηση του σπιτιού, βρήκε το καλάθι για το σαλόνι κομμένο μέσα στον κάδο απορριμμάτων.

b. Όταν ο Σπύρος έφτιαχνε τη διακόσμηση του σπιτιού, βρήκε το καλάθι για το σαλόνι κομμένη μέσα στον κάδο απορριμμάτων.

c. Όταν ο Σπύρος έφτιαχνε τη διακόσμηση του σπιτιού, βρήκε το καλάθι για την εξοχή κομμένο μέσα στον κάδο απορριμμάτων.

d. Όταν ο Σπύρος έφτιαχνε τη διακόσμηση του σπιτιού, βρήκε το καλάθι για την εξοχή κομμένη μέσα στον κάδο απορριμμάτων.

“When Spyros made the home decoration, he found the basket for the living room/countryside cut into the trash.”

Item 21a

a. Καθώς ο Γιάννης περίμενε τους οικοδόμους να τελειώσουν τη δουλειά, είδε το πατάκι για το δάπεδο ξεχασμένο δίπλα στην πόρτα της αυλής.

b. Καθώς ο Γιάννης περίμενε τους οικοδόμους να τελειώσουν τη δουλειά, είδε το πατάκι για το δάπεδο ξεχασμένη δίπλα στην πόρτα της αυλής.

c. Καθώς ο Γιάννης περίμενε τους οικοδόμους να τελειώσουν τη δουλειά, είδε το πατάκι για τη βεράντα ξεχασμένο δίπλα στην πόρτα της αυλής.

d. Καθώς ο Γιάννης περίμενε τους οικοδόμους να τελειώσουν τη δουλειά, είδε το πατάκι για τη βεράντα ξεχασμένη δίπλα στην πόρτα της αυλής.

“While John was waiting for the builders to finish the job, he saw the mat for the floor/veranda forgotten near the courtyard door.”

Item 22a

a. Όταν ο Δημήτρης μπήκε στο σπίτι, βρήκε το έπιπλο για το υπόγειο καμμένο δίπλα στην πολυθρόνα του σαλονιού.

b. Όταν ο Δημήτρης μπήκε στο σπίτι, βρήκε το έπιπλο για το υπόγειο καμμένη δίπλα στην πολυθρόνα του σαλονιού.

c. Όταν ο Δημήτρης μπήκε στο σπίτι, βρήκε το έπιπλο για την ταράτσα καμμένο δίπλα στην πολυθρόνα του σαλονιού.

d. Όταν ο Δημήτρης μπήκε στο σπίτι, βρήκε το έπιπλο για την ταράτσα καμμένη δίπλα στην πολυθρόνα του σαλονιού.

“When Dimitris entered the house, he found the furniture for basement/roof burnt next to the lounge chair.”

Item 23a

a. Όταν ο Λάμπρος έδινε οδηγίες στους εργάτες που κουβαλούσαν τα πράγματα της μετακόμισης, είδε το κουτί για το σαλόνι τυλιγμένο μέσα σε μια σακούλα σκουπιδιών.

b. Όταν ο Λάμπρος έδινε οδηγίες στους εργάτες που κουβαλούσαν τα πράγματα της μετακόμισης, είδε το κουτί για το σαλόνι τυλιγμένη μέσα σε μια σακούλα σκουπιδιών.

c. Όταν ο Λάμπρος έδινε οδηγίες στους εργάτες που κουβαλούσαν τα πράγματα της μετακόμισης, είδε το κουτί για την πιλοτή τυλιγμένο μέσα σε μια σακούλα σκουπιδιών.

d. Όταν ο Λάμπρος έδινε οδηγίες στους εργάτες που κουβαλούσαν τα πράγματα της μετακόμισης, είδε το κουτί για την πιλοτή τυλιγμένη μέσα σε μια σακούλα σκουπιδιών.

“When Lambros instructed the workers who were carrying the things of the move, he saw the box for the living room/pilotis wrapped in a garbage bag.”

Item 24a

a. Ο μεγάλος ποιητής που αγαπούσε πολύ τον τόπο του, είχε το ποίημα για το νησί γραμμένο μέσα σε ένα τετράδιο ποιημάτων.

b. Ο μεγάλος ποιητής που αγαπούσε πολύ τον τόπο του, είχε το ποίημα για το νησί γραμμένη μέσα σε ένα τετράδιο ποιημάτων.

c. Ο μεγάλος ποιητής που αγαπούσε πολύ τον τόπο του, είχε το ποίημα για την πόλη γραμμένο μέσα σε ένα τετράδιο ποιημάτων.

d. Ο μεγάλος ποιητής που αγαπούσε πολύ τον τόπο του, είχε το ποίημα για την πόλη γραμμένη μέσα σε ένα τετράδιο ποιημάτων.

“The great poet who loved his place, had the poem about the island/city written in a poem notebook.

Feminine heads

Item 1b

a. Ο Αντώνης που συμμάζευε το σπίτι, είχε τη μοκέτα για την είσοδο πλυμένη πάνω στο τραπέζι της κουζίνας.

b. Ο Αντώνης που συμμάζευε το σπίτι, είχε τη μοκέτα για την είσοδο πλυμένο πάνω στο τραπέζι της κουζίνας.

c. Ο Αντώνης που συμμάζευε το σπίτι, είχε τη μοκέτα για το ισόγειο πλυμένη πάνω στο τραπέζι της κουζίνας.

d. Ο Αντώνης που συμμάζευε το σπίτι, είχε τη μοκέτα για το ισόγειο πλυμένο πάνω στο τραπέζι της κουζίνας.

“Antonis, who was clearing the house up, had the carpet for the entrance/ground floor washed on the kitchen table.”

Item 2b

a. Όταν ο Αλέξανδρος επέστρεψε από το σούπερ-μάρκετ, είδε την ζύμη για την πίτσα ληγμένη μέσα στη σακούλα με τα ψώνια.

b. Όταν ο Αλέξανδρος επέστρεψε από το σούπερ-μάρκετ, είδε την ζύμη για την πίτσα ληγμένο μέσα στη σακούλα με τα ψώνια.

c. Όταν ο Αλέξανδρος επέστρεψε από το σούπερ-μάρκετ, είδε την ζύμη για το ψωμί ληγμένη μέσα στη σακούλα με τα ψώνια.

d. Όταν ο Αλέξανδρος επέστρεψε από το σούπερ-μάρκετ, είδε την ζύμη για το ψωμί ληγμένο μέσα στη σακούλα με τα ψώνια.

“When Alexandros returned from the supermarket, he saw the dough for the pizza/bread expired into the shopping bag.”

Item 3b

a. Ο διάσημος δικηγόρος της πολυεθνικής που γιόρταζε είκοσι χρόνια γάμου είχε την τούρτα για την επέτειο τυλιγμένη μέσα σε φύλλα αλουμινόχαρτου.

b. Ο διάσημος δικηγόρος της πολυεθνικής που γιόρταζε είκοσι χρόνια γάμου είχε την τούρτα για την επέτειο τυλιγμένο μέσα σε φύλλα αλουμινόχαρτου.

c. Ο διάσημος δικηγόρος της πολυεθνικής που γιόρταζε είκοσι χρόνια γάμου είχε την τούρτα για το γραφείο τυλιγμένη μέσα σε φύλλα αλουμινόχαρτου.

d. Ο διάσημος δικηγόρος της πολυεθνικής που γιόρταζε είκοσι χρόνια γάμου είχε την τούρτα για το γραφείο τυλιγμένο μέσα σε φύλλα αλουμινόχαρτου.

“The famous lawyer of the multinational company who celebrated twenty years of marriage had the cake for the anniversary/office wrapped in aluminium foil sheets.”

Item 4b

a. Ο γραμματέας του υπουργού που έφτασε νωρίτερα από τους καλεσμένους είδε την αφίσα για τη γιορτή καμμένη μέσα σε μια σακούλα σκουπιδιών.

b. Ο γραμματέας του υπουργού που έφτασε νωρίτερα από τους καλεσμένους είδε την αφίσα για τη γιορτή καμμένο μέσα σε μια σακούλα σκουπιδιών.

c. Ο γραμματέας του υπουργού που έφτασε νωρίτερα από τους καλεσμένους είδε την αφίσα για το πάρτι καμμένη μέσα σε μια σακούλα σκουπιδιών.

d. Ο γραμματέας του υπουργού που έφτασε νωρίτερα από τους καλεσμένους είδε την αφίσα για το πάρτι καμμένο μέσα σε μια σακούλα σκουπιδιών.

“The secretary of the minister, who arrived earlier than the guests, saw the poster for the feast/party burnt in a garbage bag.”

Item 5b

a. Όταν ο φύλακας τελείωσε τη βάρδια στην κήπο, βρήκε την πόρτα για την έξοδο στολισμένη δίπλα στο ασανσέρ του προσωπικού.

b. Όταν ο φύλακας τελείωσε τη βάρδια στην κήπο, βρήκε την πόρτα για την έξοδο στολισμένο δίπλα στο ασανσέρ του προσωπικού.

c. Όταν ο φύλακας τελείωσε τη βάρδια στην κήπο, βρήκε την πόρτα για το κτίριο στολισμένη δίπλα στο ασανσέρ του προσωπικού.

d. Όταν ο φύλακας τελείωσε τη βάρδια στην κήπο, βρήκε την πόρτα για το κτίριο στολισμένο δίπλα στο ασανσέρ του προσωπικού.

“When the guard finished the shift in the garden, he found the door for the exit/building decorated next to the elevator.”

Item 6b

a. Καθώς ο υπάλληλος τακτοποιούσε τα πράγματα στο μαγαζί, είδε την ομπρέλα για τη βιτρίνα χαλασμένη πάνω στο γραφείο του αφεντικού του.

b. Καθώς ο υπάλληλος τακτοποιούσε τα πράγματα στο μαγαζί, είδε την ομπρέλα για τη βιτρίνα χαλασμένο πάνω στο γραφείο του αφεντικού του.

c. Καθώς ο υπάλληλος τακτοποιούσε τα πράγματα στο μαγαζί, είδε την ομπρέλα για το μπαλκόνι χαλασμένη πάνω στο γραφείο του αφεντικού του.

d. Καθώς ο υπάλληλος τακτοποιούσε τα πράγματα στο μαγαζί, είδε την ομπρέλα για το μπαλκόνι χαλασμένο πάνω στο γραφείο του αφεντικού του.

“While the clerk was clearing the shop up, he saw the umbrella for the showcase/balcony damaged on his boss's desk.”

Item 7b

a. Ο ζωγράφος που αγαπούσε τον τόπο του και ταξίδευε σε ασυνήθιστους προορισμούς, είχε τη διαδρομή για την έρημο γραμμένη μέσα στο μπλοκ ζωγραφικής του.

b. Ο ζωγράφος που αγαπούσε τον τόπο του και ταξίδευε σε ασυνήθιστους προορισμούς, είχε τη διαδρομή για την έρημο γραμμένο μέσα στο μπλοκ ζωγραφικής του.

c. Ο ζωγράφος που αγαπούσε τον τόπο του και ταξίδευε σε ασυνήθιστους προορισμούς, είχε τη διαδρομή για το γήπεδο γραμμένη μέσα στο μπλοκ ζωγραφικής του.

d. Ο ζωγράφος που αγαπούσε τον τόπο του και ταξίδευε σε ασυνήθιστους προορισμούς, είχε τη διαδρομή για το γήπεδο γραμμένο μέσα στο μπλοκ ζωγραφικής του.

“The painter who loved his place and traveled to unusual destinations, had the route to the desert/stadium written in his painting block.”

Item 8b

a. Ο συμπαθητικός τουρίστας που είχε χαθεί ψάχνοντας για αξιοθέατα, είδε την ταμπέλα για τη λεωφόρο ραγισμένη πάνω στη γέφυρα των τρένων.

b. Ο συμπαθητικός τουρίστας που είχε χαθεί ψάχνοντας για αξιοθέατα, είδε την ταμπέλα για τη λεωφόρο ραγισμένο πάνω στη γέφυρα των τρένων.

c. Ο συμπαθητικός τουρίστας που είχε χαθεί ψάχνοντας για αξιοθέατα, είδε την ταμπέλα για το μουσείο ραγισμένη πάνω στη γέφυρα των τρένων.

d. Ο συμπαθητικός τουρίστας που είχε χαθεί ψάχνοντας για αξιοθέατα, είδε την ταμπέλα για το μουσείο ραγισμένο πάνω στη γέφυρα των τρένων.

“The sympathetic tourist, who was lost looking for sightseeing, saw the sign for the avenue/museum cracked on the train bridge.”

Item 9b

a. Όταν ο Αντρέας συμμάζευε το εξοχικό του, είδε την πετσέτα για την άμμο κομμένη δίπλα στο κουτί του απορρυπαντικού.

b. Όταν ο Αντρέας συμμάζευε το εξοχικό του, είδε την πετσέτα για την άμμο κομμένο δίπλα στο κουτί του απορρυπαντικού.

c. Όταν ο Αντρέας συμμάζευε το εξοχικό του, είδε την πετσέτα για το μπάνιο κομμένη δίπλα στο κουτί του απορρυπαντικού.

d. Όταν ο Αντρέας συμμάζευε το εξοχικό του, είδε την πετσέτα για το μπάνιο κομμένο δίπλα στο κουτί του απορρυπαντικού.

“When Andreas was clearing his cottage up, he saw the towel for the bathroom/sand cut next to the detergent box.”

Item 10b

a. Ο θυρωρός που είχε διαβάσει το δελτίο καιρού με τα ακραία φαινόμενα, είχε την ανακοίνωση για τη σκόνη κρεμασμένη πάνω στον πίνακα ανακοινώσεων της πολυκατοικίας.

b. Ο θυρωρός που είχε διαβάσει το δελτίο καιρού με τα ακραία φαινόμενα, είχε την ανακοίνωση για τη σκόνη κρεμασμένο πάνω στον πίνακα ανακοινώσεων της πολυκατοικίας.

c. Ο θυρωρός που είχε διαβάσει το δελτίο καιρού με τα ακραία φαινόμενα, είχε την ανακοίνωση για το χιόνι κρεμασμένη πάνω στον πίνακα ανακοινώσεων της πολυκατοικίας.

d. Ο θυρωρός που είχε διαβάσει το δελτίο καιρού με τα ακραία φαινόμενα, είχε την ανακοίνωση για το χιόνι κρεμασμένο πάνω στον πίνακα ανακοινώσεων της πολυκατοικίας.

“The concierge, who had read the weather report with the extreme phenomena, had the dust/snow report hanging on the panel of the block of flats.”

Item 11b

a. Όταν ο υπομονετικός ράφτης επέστρεψε στη δουλειά του είδε, την κλωστή για την κουρτίνα βρεγμένη δίπλα στο κουτί με τα ραφτικά.

b. Όταν ο υπομονετικός ράφτης επέστρεψε στη δουλειά του είδε, την κλωστή για την κουρτίνα βρεγμένο δίπλα στο κουτί με τα ραφτικά.

c. Όταν ο υπομονετικός ράφτης επέστρεψε στη δουλειά του είδε, την κλωστή για το φόρεμα βρεγμένη δίπλα στο κουτί με τα ραφτικά.

d. Όταν ο υπομονετικός ράφτης επέστρεψε στη δουλειά του είδε, την κλωστή για το φόρεμα βρεγμένο δίπλα στο κουτί με τα ραφτικά.

“When the patient tailor returned to work, he saw the thread for the curtain/dress wet next to the sewing box.”

Item 12b

a. Όταν ο διάσημος ενδυματολόγος επέστρεψε στο μαγαζί του, βρήκε την απόδειξη για τη γραβάτα σκισμένη πάνω στο γραφείο του γραμματέα του.

b. Όταν ο διάσημος ενδυματολόγος επέστρεψε στο μαγαζί του, βρήκε την απόδειξη για τη γραβάτα σκισμένο πάνω στο γραφείο του γραμματέα του.

c. Όταν ο διάσημος ενδυματολόγος επέστρεψε στο μαγαζί του, βρήκε την απόδειξη για το φόρεμα σκισμένη πάνω στο γραφείο του γραμματέα του.

d. Όταν ο διάσημος ενδυματολόγος επέστρεψε στο μαγαζί του, βρήκε την απόδειξη για το φόρεμα σκισμένο πάνω στο γραφείο του γραμματέα του.

“When the famous stylist returned to his shop, he found the recipe for the tie/dress torn on his secretary's desk.”

Item 13b

a. Ο Παύλος που δεν πρόλαβε να συγυρίσει το σπίτι, είχε τη στολή για τη γιορτή πεταμένη δίπλα στο κουστούμι του γάμου του.

b. Ο Παύλος που δεν πρόλαβε να συγυρίσει το σπίτι, είχε τη στολή για τη γιορτή πεταμένο δίπλα στο κουστούμι του γάμου του.

c. Ο Παύλος που δεν πρόλαβε να συγυρίσει το σπίτι, είχε τη στολή για το χιόνι πεταμένη δίπλα στο κουστούμι του γάμου του.

d. Ο Παύλος που δεν πρόλαβε να συγυρίσει το σπίτι, είχε τη στολή για το χιόνι πεταμένο δίπλα στο κουστούμι του γάμου του.

“Paul, who failed to confront the house, had the outfit for the feast/snow thrown next to his wedding suit.”

Item 14b

a. Ο διάσημος ιστορικός που ήταν πολλά χρόνια παντρεμένος, είδε την πρόσκληση για την επέτειο ανοιγμένη δίπλα στο άδειο μπουκάλι ουίσκι.

b. Ο διάσημος ιστορικός που ήταν πολλά χρόνια παντρεμένος, είδε την πρόσκληση για την επέτειο ανοιγμένο δίπλα στο άδειο μπουκάλι ουίσκι.

c. Ο διάσημος ιστορικός που ήταν πολλά χρόνια παντρεμένος, είδε την πρόσκληση για το μουσείο ανοιγμένη δίπλα στο άδειο μπουκάλι ουίσκι.

d. Ο διάσημος ιστορικός που ήταν πολλά χρόνια παντρεμένος, είδε την πρόσκληση για το μουσείο ανοιγμένο δίπλα στο άδειο μπουκάλι ουίσκι.

“The famous historian, who was married for many years, saw the invitation to the museum/anniversary opened next to the empty whiskey bottle.”

Item 15b

a. Όταν ο Αλέξανδρος επέστρεψε από την αγορά, βρήκε τη σκούπα για την άμμο σπασμένη πάνω στο τραπέζι του σαλονιού.

b. Όταν ο Αλέξανδρος επέστρεψε από την αγορά, βρήκε τη σκούπα για την άμμο σπασμένο πάνω στο τραπέζι του σαλονιού.

c. Όταν ο Αλέξανδρος επέστρεψε από την αγορά, βρήκε τη σκούπα για το μπάνιο σπασμένη πάνω στο τραπέζι του σαλονιού.

d. Όταν ο Αλέξανδρος επέστρεψε από την αγορά, βρήκε τη σκούπα για το μπάνιο σπασμένο πάνω στο τραπέζι του σαλονιού.

“When Alexandros returned from the market, he found the broom for the sand/bathroom broken on his coffee table.”

Item 16b

a. Ο υπάλληλος που παρέλαβε τη μυστική παραγγελία, βρήκε την καρφίτσα για την κουρτίνα σκεπασμένη μέσα σε μια κούτα με ρούχα.

b. Ο υπάλληλος που παρέλαβε τη μυστική παραγγελία, βρήκε την καρφίτσα για την κουρτίνα σκεπασμένο μέσα σε μια κούτα με ρούχα.

c. Ο υπάλληλος που παρέλαβε τη μυστική παραγγελία, βρήκε την καρφίτσα για το φουστάνι σκεπασμένη μέσα σε μια κούτα με ρούχα.

d. Ο υπάλληλος που παρέλαβε τη μυστική παραγγελία, βρήκε την καρφίτσα για το φουστάνι σκεπασμένο μέσα σε μια κούτα με ρούχα.

“The employee who received the secret order found the pin for the curtain/dress covered in a box of clothes.”

Item 17b

a. Καθώς ο Γιάννης έφευγε από το παλιό σπίτι, βρήκε τη σκάλα για την έξοδο σκονισμένη δίπλα στον πίνακα του διάσημου ζωγράφου.

b. Καθώς ο Γιάννης έφευγε από το παλιό σπίτι, βρήκε τη σκάλα για την έξοδο σκονισμένο δίπλα στον πίνακα του διάσημου ζωγράφου.

c. Καθώς ο Γιάννης έφευγε από το παλιό σπίτι, βρήκε τη σκάλα για το ισόγειο σκονισμένη δίπλα στον πίνακα του διάσημου ζωγράφου.

d. Καθώς ο Γιάννης έφευγε από το παλιό σπίτι, βρήκε τη σκάλα για το ισόγειο σκονισμένο δίπλα στον πίνακα του διάσημου ζωγράφου.

“While Giannis was leaving the old house, he found the stairway for the exit/ground floor dusty next to the famous painter's painting.”

Item 18b

a. Ο απρόσεκτος μάγειρας που έχανε τα πράγματά του συνεχώς είχε τη συνταγή για την πίτσα ξεχασμένη πάνω στο ράφι της κουζίνας.

b. Ο απρόσεκτος μάγειρας που έχανε τα πράγματά του συνεχώς είχε τη συνταγή για την πίτσα ξεχασμένο πάνω στο ράφι της κουζίνας.

c. Ο απρόσεκτος μάγειρας που έχανε τα πράγματά του συνεχώς είχε τη συνταγή για το ψωμί ξεχασμένη πάνω στο ράφι της κουζίνας.

d. Ο απρόσεκτος μάγειρας που έχανε τα πράγματά του συνεχώς είχε τη συνταγή για το ψωμί ξεχασμένο πάνω στο ράφι της κουζίνας.

“The careless cook who lost his stuff constantly had the recipe for the pizza/bread forgotten on the kitchen shelf.”

Item 19b

a. Καθώς ο διακοσμητής έψαχνε τα κατάλληλα αντικείμενα για τη διακόσμηση του σπιτιού, βρήκε τη γλάστρα για τη βιτρίνα καρφωμένη πάνω στο ράφι της βιβλιοθήκης.

b. Καθώς ο διακοσμητής έψαχνε τα κατάλληλα αντικείμενα για τη διακόσμηση του σπιτιού, βρήκε τη γλάστρα για τη βιτρίνα καρφωμένο πάνω στο ράφι της βιβλιοθήκης.

c. Καθώς ο διακοσμητής έψαχνε τα κατάλληλα αντικείμενα για τη διακόσμηση του σπιτιού, βρήκε τη γλάστρα για το μπαλκόνι καρφωμένη πάνω στο ράφι της βιβλιοθήκης.

d. Καθώς ο διακοσμητής έψαχνε τα κατάλληλα αντικείμενα για τη διακόσμηση του σπιτιού, βρήκε τη γλάστρα για το μπαλκόνι καρφωμένο πάνω στο ράφι της βιβλιοθήκης.

“As the decorator was looking for the appropriate items for decorating the house, he found the flower pot for the showcase/balcony nailed on the library shelf.”

Item 20b

a. Ο ακούραστος τουρίστας που ήθελε να επισκεφτεί διάφορους τόπους και αξιοθέατα, βρήκε την πινακίδα για την έρημο λασπωμένη μέσα στην πυκνή βλάστηση της περιοχής.

b. Ο ακούραστος τουρίστας που ήθελε να επισκεφτεί διάφορους τόπους και αξιοθέατα, βρήκε την πινακίδα για την έρημο λασπωμένο μέσα στην πυκνή βλάστηση της περιοχής.

c. Ο ακούραστος τουρίστας που ήθελε να επισκεφτεί διάφορους τόπους και αξιοθέατα, βρήκε την πινακίδα για το κτίριο λασπωμένη μέσα στην πυκνή βλάστηση της περιοχής.

d. Ο ακούραστος τουρίστας που ήθελε να επισκεφτεί διάφορους τόπους και αξιοθέατα, βρήκε την πινακίδα για το κτίριο λασπωμένο μέσα στην πυκνή βλάστηση της περιοχής.

“The tireless tourist, who wanted to visit various places and sights, found the billboard for the desert/building muddy in the dense vegetation of the area.”

Item 21b

a. Λόγω των έργων που γίνονται στην περιοχή ο Κώστας βρήκε την κατεύθυνση για τη λεωφόρο κλεισμένη δίπλα στους γερανούς της κοινότητας.

b. Λόγω των έργων που γίνονται στην περιοχή ο Κώστας βρήκε την κατεύθυνση για τη λεωφόρο κλεισμένο δίπλα στους γερανούς της κοινότητας.

c. Λόγω των έργων που γίνονται στην περιοχή ο Κώστας βρήκε την κατεύθυνση για το γραφείο κλεισμένη δίπλα στους γερανούς της κοινότητας.

d. Λόγω των έργων που γίνονται στην περιοχή ο Κώστας βρήκε την κατεύθυνση για το γραφείο κλεισμένο δίπλα στους γερανούς της κοινότητας.

“Due to the works made in the area, Kostas found the direction for the avenue/office closed next to the tow trucks of the township.”

Item 22b

a. Ο νεαρός που έπαιζε στις λάσπες με τους φίλους του είχε την κάρτα για την είσοδο λερωμένη μέσα στο μπουφάν του.

b. Ο νεαρός που έπαιζε στις λάσπες με τους φίλους του είχε την κάρτα για την είσοδο λερωμένο μέσα στο μπουφάν του.

c. Ο νεαρός που έπαιζε στις λάσπες με τους φίλους του είχε την κάρτα για το γήπεδο λερωμένη μέσα στο μπουφάν του.

d. Ο νεαρός που έπαιζε στις λάσπες με τους φίλους του είχε την κάρτα για το γήπεδο λερωμένο μέσα στο μπουφάν του.

“The young man who played in the mud with his friends had the card for the entrance/pitch dirty in his jacket.”

Item 23b

a. Ο διάσημος σχεδιαστής μόδας που επέλεγε ασυνήθιστους συνδυασμούς ρούχων, είδε την κορδέλα για τη γραβάτα πεταμένη δίπλα στο κουτί με τις βελόνες.

b. Ο διάσημος σχεδιαστής μόδας που επέλεγε ασυνήθιστους συνδυασμούς ρούχων, είδε την κορδέλα για τη γραβάτα πεταμένο δίπλα στο κουτί με τις βελόνες.

c. Ο διάσημος σχεδιαστής μόδας που επέλεγε ασυνήθιστους συνδυασμούς ρούχων, είδε την κορδέλα για το φουστάνι πεταμένη δίπλα στο κουτί με τις βελόνες.

d. Ο διάσημος σχεδιαστής μόδας που επέλεγε ασυνήθιστους συνδυασμούς ρούχων, είδε την κορδέλα για το φουστάνι πεταμένο δίπλα στο κουτί με τις βελόνες.

“The famous fashion designer who was choosing unusual combinations of clothes, saw the ribbon for the tie/dress shuffled next to the needle box.”

Item 24b

a. Ο Πέτρος που ετοιμαζόταν να βγει έξω, είχε τη μάσκα για τη σκόνη δεμένη μέσα στο μπουφάν που φορούσε.

b. Ο Πέτρος που ετοιμαζόταν να βγει έξω, είχε τη μάσκα για τη σκόνη δεμένο μέσα στο μπουφάν που φορούσε.

c. Ο Πέτρος που ετοιμαζόταν να βγει έξω, είχε τη μάσκα για το χιόνι δεμένη μέσα στο μπουφάν που φορούσε.

d. Ο Πέτρος που ετοιμαζόταν να βγει έξω, είχε τη μάσκα για το χιόνι δεμένο μέσα στο μπουφάν που φορούσε.

“Peter, who was about to get out, had the mask for the dust/snow tied up in the jacket he was wearing.”

Experiment 2

Item 1a

a. Όταν ο Πέτρος τελείωσε τις δουλειές του σπιτιού γύρευε το χαλί για το πάτωμα και το βρήκε δίπλα στο λάστιχο του κήπου.

b. Όταν ο Πέτρος τελείωσε τις δουλειές του σπιτιού γύρευε το χαλί για το πάτωμα και τη βρήκε δίπλα στο λάστιχο του κήπου.

c. Όταν ο Πέτρος τελείωσε τις δουλειές του σπιτιού γύρευε το χαλί για την κουζίνα και το βρήκε δίπλα στο λάστιχο του κήπου.

d. Όταν ο Πέτρος τελείωσε τις δουλειές του σπιτιού γύρευε το χαλί για την κουζίνα και τη βρήκε δίπλα στο λάστιχο του κήπου.

“When Peter finished the housework, he was looking for the carpet for the floor/kitchen and he found it near the hose of the garden”.

Item 2a

a. Ο μάγειρας που διαφήμιζε στον κόσμο τις νέες συνταγές του αναζητούσε το φαγητό το φαγητό για το μαγαζί και το βρήκε μέσα στο ταψί του βοηθού του.

b. Ο μάγειρας που διαφήμιζε στον κόσμο τις νέες συνταγές του αναζητούσε το φαγητό το φαγητό για το μαγαζί και τη βρήκε μέσα στο ταψί του βοηθού του.

c. Ο μάγειρας που διαφήμιζε στον κόσμο τις νέες συνταγές του αναζητούσε το φαγητό το φαγητό για την έκθεση και τη βρήκε μέσα στο ταψί του βοηθού του.

d. Ο μάγειρας που διαφήμιζε στον κόσμο τις νέες συνταγές του αναζητούσε το φαγητό το φαγητό για την έκθεση και τη βρήκε μέσα στο ταψί του βοηθού του.

“The cook, who was advertising his new recipes to the crowd, he was looking for the food for the store/exhibition and he found it in his assistant’s baking tray”.

Item 3a

a. Όταν ο Κώστας μπήκε στην κουζίνα έψαχνε το αυγό για το γλυκό και το είδε δίπλα στο κουτί με τις σοκολάτες.

b. Όταν ο Κώστας μπήκε στην κουζίνα έψαχνε το αυγό για το γλυκό και την είδε δίπλα στο κουτί με τις σοκολάτες.

c. Όταν ο Κώστας μπήκε στην κουζίνα έψαχνε το αυγό για τη σούπα και το είδε δίπλα στο κουτί με τις σοκολάτες.

d. Όταν ο Κώστας μπήκε στην κουζίνα έψαχνε το αυγό για τη σούπα και την είδε δίπλα στο κουτί με τις σοκολάτες.

“When Kostas went into the kitchen, he was looking for the egg for the dessert/soup and he saw it next to the box with the chocolates”.

Item 4a

a. Ο αργοπορημένος επιβάτης γύρευε το εισιτήριο για το ταξίδι και το ανακάλυψε μέσα στην τσάντα της γυναίκας του.

b. Ο αργοπορημένος επιβάτης γύρευε το εισιτήριο για το ταξίδι και το ανακάλυψε μέσα στην τσάντα της γυναίκας του.

c. Ο αργοπορημένος επιβάτης γύρευε το εισιτήριο για την εκδρομή και το ανακάλυψε μέσα στην τσάντα της γυναίκας του.

d. Ο αργοπορημένος επιβάτης γύρευε το εισιτήριο για την εκδρομή και το ανακάλυψε μέσα στην τσάντα της γυναίκας του.

“The late passenger was looking for the ticket for the trip/excursion and he discovered it in his wife’s bag”.

Item 5a

a. Καθώς ο Πέτρος έστρωνε το τραπέζι αναζητούσε το κουτάλι για το γλυκό γύρευε και το εντόπισε πάνω στον πάγκο της κουζίνας.

b. Καθώς ο Πέτρος έστρωνε το τραπέζι αναζητούσε το κουτάλι για το γλυκό και την εντόπισε πάνω στον πάγκο της κουζίνας.

c. Καθώς ο Πέτρος έστρωνε το τραπέζι αναζητούσε το κουτάλι για τη σούπα και το εντόπισε πάνω στον πάγκο της κουζίνας.

d. Καθώς ο Πέτρος έστρωνε το τραπέζι αναζητούσε το κουτάλι για τη σούπα και την εντόπισε πάνω στον πάγκο της κουζίνας.

“When Kostas was setting the table, he was looking for the spoon for the desert/soup and he found it on the kitchen counter.”

Item 6a

a. Καθώς ο Κώστας ετοιμαζόταν να φύγει, έψαχνε το κλειδί για το αμάξι και το ανακάλυψε μέσα στο συρτάρι του κομοδίνου.

b. Καθώς ο Κώστας ετοιμαζόταν να φύγει, έψαχνε το κλειδί για το αμάξι και την ανακάλυψε μέσα στο συρτάρι του κομοδίνου.

c. Καθώς ο Κώστας ετοιμαζόταν να φύγει, έψαχνε το κλειδί για τη μηχανή και το ανακάλυψε μέσα στο συρτάρι του κομοδίνου.

d. Καθώς ο Κώστας ετοιμαζόταν να φύγει, έψαχνε το κλειδί για τη μηχανή και την ανακάλυψε μέσα στο συρτάρι του κομοδίνου.

“When Kostas was about to leave, he was looking for the key for the car/motorbike and he found it in the drawer of the bedside table.”

Item 7a

a. O Βασίλης που φοβόταν πολύ το σκοτάδι, γύρευε το φανάρι για το πατάρι και το εντόπισε δίπλα στις λαμπάδες της Ανάστασης.

b. O Βασίλης που φοβόταν πολύ το σκοτάδι, γύρευε το φανάρι για το πατάρι και την εντόπισε δίπλα στις λαμπάδες της Ανάστασης.

c. O Βασίλης που φοβόταν πολύ το σκοτάδι, γύρευε το φανάρι για την εξοχή και το εντόπισε δίπλα στις λαμπάδες της Ανάστασης.

d. O Βασίλης που φοβόταν πολύ το σκοτάδι, γύρευε το φανάρι για την εξοχή και την εντόπισε δίπλα στις λαμπάδες της Ανάστασης.

“Vasilis, who was afraid of the dark, was looking for the lantern for the attic/countryside and he found it next to the lampades (candles of the Resurrection).”

Item 8a

a. Όταν ο Πέτρος έφτασε στο ακριβό ξενοδοχείο που θα απολάμβανε τις διακοπές του, αναζητούσε το μπουρνούζι για το δωμάτιο και το βρήκε δίπλα στο γραφείο της ρεσεψιόν.

b. Όταν ο Πέτρος έφτασε στο ακριβό ξενοδοχείο που θα απολάμβανε τις διακοπές του, αναζητούσε το μπουρνούζι για το δωμάτιο και τη βρήκε δίπλα στο γραφείο της ρεσεψιόν.

c. Όταν ο Πέτρος έφτασε στο ακριβό ξενοδοχείο που θα απολάμβανε τις διακοπές του, αναζητούσε το μπουρνούζι για την πισίνα και το βρήκε δίπλα στο γραφείο της ρεσεψιόν.

d. Όταν ο Πέτρος έφτασε στο ακριβό ξενοδοχείο που θα απολάμβανε τις διακοπές του, αναζητούσε το μπουρνούζι για την πισίνα και τη βρήκε δίπλα στο γραφείο της ρεσεψιόν.

“When Petros arrived at the expensive hotel where he would enjoy his vacation, his was looking for the bathrobe for the room/pool and he found it near the reception desk”.

Item 9a

a. Όταν ο Δημήτρης επέστρεψε στο σπίτι, έψαχνε το φωτιστικό για το δάπεδο και το ανακάλυψε μέσα στο κουτί της μετακόμισης.

b. Όταν ο Δημήτρης επέστρεψε στο σπίτι, έψαχνε το φωτιστικό για το δάπεδο και την ανακάλυψε μέσα στο κουτί της μετακόμισης.

c. Όταν ο Δημήτρης επέστρεψε στο σπίτι, έψαχνε το φωτιστικό για την ταράτσα και το ανακάλυψε μέσα στο κουτί της μετακόμισης.

d. Όταν ο Δημήτρης επέστρεψε στο σπίτι, έψαχνε το φωτιστικό για την ταράτσα και την ανακάλυψε μέσα στο κουτί της μετακόμισης.

“When Dimitris went back home, he was looking for the lamp for the floor/terrace and he discovered it in the box of the move.”

Item 10a

a. Ο Μάνος που έδωσε εντολή να τακτοποιηθούν όλα τα πράγματα, γύρευε το κιβώτιο για το πατάρι και το εντόπισε πάνω στον καναπέ του σαλονιού.

b. Ο Μάνος που έδωσε εντολή να τακτοποιηθούν όλα τα πράγματα, γύρευε το κιβώτιο για το πατάρι και την εντόπισε πάνω στον καναπέ του σαλονιού.

c. Ο Μάνος που έδωσε εντολή να τακτοποιηθούν όλα τα πράγματα, γύρευε το κιβώτιο για την πιλοτή και το εντόπισε πάνω στον καναπέ του σαλονιού.

d. Ο Μάνος που έδωσε εντολή να τακτοποιηθούν όλα τα πράγματα, γύρευε το κιβώτιο για την πιλοτή και την εντόπισε πάνω στον καναπέ του σαλονιού.

“Manos who ordered all things be arranged, was looking for the box for the attic/pilotis and he found it on the living room sofa.”

Item 11a

a. Ο Πέτρος που ήθελε να διαβάσει περισσότερες πληροφορίες για τον τόπο που μεγάλωσε, αναζητούσε το περιοδικό για το νησί και το βρήκε μέσα στον κάδο απορριμμάτων της πολυκατοικίας.

b. Ο Πέτρος που ήθελε να διαβάσει περισσότερες πληροφορίες για τον τόπο που μεγάλωσε, αναζητούσε το περιοδικό για το νησί και τη βρήκε μέσα στον κάδο απορριμμάτων της πολυκατοικίας.

c. Ο Πέτρος που ήθελε να διαβάσει περισσότερες πληροφορίες για τον τόπο που μεγάλωσε, αναζητούσε το περιοδικό για την πόλη και το βρήκε μέσα στον κάδο απορριμμάτων της πολυκατοικίας.

d. Ο Πέτρος που ήθελε να διαβάσει περισσότερες πληροφορίες για τον τόπο που μεγάλωσε, αναζητούσε το περιοδικό για την πόλη και τη βρήκε μέσα στον κάδο απορριμμάτων της πολυκατοικίας.

“Petros who wanted to read more about the place he grew up, was looking for the magazine for the island/town and he found it into the garbage bin of the block of flats.”

Item 12a

a. Καθώς ο Μάνος περίμενε τον ηλεκτρολόγο, έψαχνε το καλώδιο για το υπόγειο και το είδε πάνω στον καλόγερο της εισόδου.

b. Καθώς ο Μάνος περίμενε τον ηλεκτρολόγο, έψαχνε το καλώδιο για το υπόγειο και την είδε πάνω στον καλόγερο της εισόδου.

c. Καθώς ο Μάνος περίμενε τον ηλεκτρολόγο, έψαχνε το καλώδιο για την ταράτσα και το είδε πάνω στον καλόγερο της εισόδου.

d. Καθώς ο Μάνος περίμενε τον ηλεκτρολόγο, έψαχνε το καλώδιο για την ταράτσα και την είδε πάνω στον καλόγερο της εισόδου.

“While Manos was waiting for the electrician, he was looking for the cable for the basement/terrace and he found it on coat hanger of the entrance.”

Item 13a

a. Ο ιδιοκτήτης της επιχείρησης που ήταν απατεώνας, γύρευε το εμπόρευμα για το μαγαζί και το εντόπισε πάνω στο καρότσι του σούπερ-μάρκετ.

b. Ο ιδιοκτήτης της επιχείρησης που ήταν απατεώνας, γύρευε το εμπόρευμα για το μαγαζί και την εντόπισε πάνω στο καρότσι του σούπερ-μάρκετ.

c. Ο ιδιοκτήτης της επιχείρησης που ήταν απατεώνας, γύρευε το εμπόρευμα για την έκθεση και το εντόπισε πάνω στο καρότσι του σούπερ-μάρκετ.

d. Ο ιδιοκτήτης της επιχείρησης που ήταν απατεώνας, γύρευε το εμπόρευμα για την έκθεση και την εντόπισε πάνω στο καρότσι του σούπερ-μάρκετ.

“The owner of the company who was a scammer, was looking for the commodity for the store/exhibition and he found it on the supermarket stroller.”

Item 14a

a. Ο μάγειρας που προσπαθούσε να εκπαιδεύσει τους τεμπέληδες βοηθούς του, αναζητούσε το ράφι για το γάλα και το εντόπισε δίπλα στον φούρνο μικροκυμάτων.

b. Ο μάγειρας που προσπαθούσε να εκπαιδεύσει τους τεμπέληδες βοηθούς του, αναζητούσε το ράφι για το γάλα και την εντόπισε δίπλα στον φούρνο μικροκυμάτων.

c. Ο μάγειρας που προσπαθούσε να εκπαιδεύσει τους τεμπέληδες βοηθούς του, αναζητούσε το ράφι για την κρέμα και το εντόπισε δίπλα στον φούρνο μικροκυμάτων.

d. Ο μάγειρας που προσπαθούσε να εκπαιδεύσει τους τεμπέληδες βοηθούς του, αναζητούσε το ράφι για την κρέμα και την εντόπισε δίπλα στον φούρνο μικροκυμάτων.

“The cook, who was trying to train his lazy assistants, was looking for the shelf for the milk and he found it next to the microwave.”

Item 15a

a. Καθώς ο Παύλος ετοίμαζε το βραδινό του, έψαχνε το μέλι για το γάλα και το είδε δίπλα στο κέικ σοκολάτας.

b. Καθώς ο Παύλος ετοίμαζε το βραδινό του, έψαχνε το μέλι για το γάλα και την είδε δίπλα στο κέικ σοκολάτας.

c. Καθώς ο Παύλος ετοίμαζε το βραδινό του, έψαχνε το μέλι για την κρέμα και το είδε δίπλα στο κέικ σοκολάτας.

d. Καθώς ο Παύλος ετοίμαζε το βραδινό του, έψαχνε το μέλι για την κρέμα και την είδε δίπλα στο κέικ σοκολάτας.

“While Paul was preparing his dinner, he was looking for the honey for the milk/cream and he saw it next to the chocolate cake.”

Item 16a

a. Ο Αλέξης που περίμενε περισσότερα υλικά για την επιδιόρθωση της ζημιάς. γύρευε το πλακάκι για το πάτωμα και το ανακάλυψε δίπλα στο κέικ σοκολάτας.

b. Ο Αλέξης που περίμενε περισσότερα υλικά για την επιδιόρθωση της ζημιάς. γύρευε το πλακάκι για το πάτωμα και τη ανακάλυψε δίπλα στο κέικ σοκολάτας.

c. Ο Αλέξης που περίμενε περισσότερα υλικά για την επιδιόρθωση της ζημιάς. γύρευε το πλακάκι για την πισίνα και το ανακάλυψε δίπλα στο κέικ σοκολάτας.

d. Ο Αλέξης που περίμενε περισσότερα υλικά για την επιδιόρθωση της ζημιάς. γύρευε το πλακάκι για την πισίνα και τη ανακάλυψε δίπλα στο κέικ σοκολάτας.

“Alexis, who was waiting for more materials to repair the damage, he was looking for the tile for the floor/swimming pool and he found it next to the chocolate cake.”

Item 17a

a. Όταν ο Βασίλης μπήκε στο σαλόνι, αναζητούσε το πρόστιμο για το αμάξι και το βρήκε δίπλα στο τραπεζάκι της τηλεόρασης.

b. Όταν ο Βασίλης μπήκε στο σαλόνι, αναζητούσε το πρόστιμο για το αμάξι και τη βρήκε δίπλα στο τραπεζάκι της τηλεόρασης.

c. Όταν ο Βασίλης μπήκε στο σαλόνι, αναζητούσε το πρόστιμο για τη μηχανή και το βρήκε δίπλα στο τραπεζάκι της τηλεόρασης.

d. Όταν ο Βασίλης μπήκε στο σαλόνι, αναζητούσε το πρόστιμο για τη μηχανή και τη βρήκε δίπλα στο τραπεζάκι της τηλεόρασης.

“When Vasilis went into the living room, he was looking for the fine for the car/motorbike and he found it next to the television table.”

Item 18a

a. O Βασίλης που είχε αρχίσει να πακετάρει, έψαχνε το ρολόι για το δωμάτιο και το είδε πάνω στον τοίχο του σαλονιού.

b. O Βασίλης που είχε αρχίσει να πακετάρει, έψαχνε το ρολόι για το δωμάτιο και την είδε πάνω στον τοίχο του σαλονιού.

c. O Βασίλης που είχε αρχίσει να πακετάρει, έψαχνε το ρολόι για την κουζίνα και το είδε πάνω στον τοίχο του σαλονιού.

d. O Βασίλης που είχε αρχίσει να πακετάρει, έψαχνε το ρολόι για την κουζίνα και την είδε πάνω στον τοίχο του σαλονιού.

“Vasilis, who had begun packing, was looking for the clock for the room/kitchen and he found it on the living room wall.”

Item 19a

a. O έμπειρος ορειβάτης που σκαρφάλωνε στα πιο ψηλά βουνά, γύρευε το σχοινί για το ταξίδι και το εντόπισε πάνω στο σακίδιο της γυναίκας του.

b. O έμπειρος ορειβάτης που σκαρφάλωνε στα πιο ψηλά βουνά, γύρευε το σχοινί για το ταξίδι και την εντόπισε πάνω στο σακίδιο της γυναίκας του.

c. O έμπειρος ορειβάτης που σκαρφάλωνε στα πιο ψηλά βουνά, γύρευε το σχοινί για την εκδρομή και το εντόπισε πάνω στο σακίδιο της γυναίκας του.

d. O έμπειρος ορειβάτης που σκαρφάλωνε στα πιο ψηλά βουνά, γύρευε το σχοινί για την εκδρομή και την εντόπισε πάνω στο σακίδιο της γυναίκας του.

“The experienced climber climbing on the highest mountains was looking for the rope for the trip/excursion and he found it on his wife's backpack.”

Item 20a

a. Όταν ο Σπύρος έφτιαχνε τη διακόσμηση του σπιτιού, αναζητούσε το καλάθι για το σαλόνι και το βρήκε μέσα στον κάδο απορριμμάτων.

b. Όταν ο Σπύρος έφτιαχνε τη διακόσμηση του σπιτιού, αναζητούσε το καλάθι για το σαλόνι και τη βρήκε μέσα στον κάδο απορριμμάτων.

c. Όταν ο Σπύρος έφτιαχνε τη διακόσμηση του σπιτιού, αναζητούσε το καλάθι για την εξοχή και το βρήκε μέσα στον κάδο απορριμμάτων.

d. Όταν ο Σπύρος έφτιαχνε τη διακόσμηση του σπιτιού, αναζητούσε το καλάθι για την εξοχή και τη βρήκε μέσα στον κάδο απορριμμάτων.

“When Spyros made the home decoration, he was looking for the basket for the living room/countryside and he found it into the trash.”

Item 21a

a. Καθώς ο Γιάννης περίμενε τους οικοδόμους να τελειώσουν τη δουλειά, έψαχνε το πατάκι για το δάπεδο και το είδε δίπλα στην πόρτα της αυλής.

b. Καθώς ο Γιάννης περίμενε τους οικοδόμους να τελειώσουν τη δουλειά, έψαχνε το πατάκι για το δάπεδο και το είδε δίπλα στην πόρτα της αυλής.

c. Καθώς ο Γιάννης περίμενε τους οικοδόμους να τελειώσουν τη δουλειά, έψαχνε το πατάκι για τη βεράντα και το είδε δίπλα στην πόρτα της αυλής.

d. Καθώς ο Γιάννης περίμενε τους οικοδόμους να τελειώσουν τη δουλειά, έψαχνε το πατάκι για τη βεράντα και το είδε δίπλα στην πόρτα της αυλής.

“While John was waiting for the builders to finish the job, he was looking for the mat for the floor/veranda and he found it near the courtyard door.”

Item 22a

a. Όταν ο Δημήτρης μπήκε στο σπίτι, γύρευε το έπιπλο για το υπόγειο και το είδε δίπλα στην πολυθρόνα του σαλονιού.

b. Όταν ο Δημήτρης μπήκε στο σπίτι, γύρευε το έπιπλο για το υπόγειο και την είδε δίπλα στην πολυθρόνα του σαλονιού.

c. Όταν ο Δημήτρης μπήκε στο σπίτι, γύρευε το έπιπλο για την ταράτσα και το είδε δίπλα στην πολυθρόνα του σαλονιού.

d. Όταν ο Δημήτρης μπήκε στο σπίτι, γύρευε το έπιπλο για την ταράτσα και την είδε δίπλα στην πολυθρόνα του σαλονιού.

“When Dimitris entered the house, he was looking for the furniture for basement/roof and he found it next to the lounge chair.”

Item 23a

a. Όταν ο Λάμπρος έδινε οδηγίες στους εργάτες που κουβαλούσαν τα πράγματα της μετακόμισης, αναζητούσε το κουτί για το σαλόνι και το είδε μέσα σε μια σακούλα σκουπιδιών.

b. Όταν ο Λάμπρος έδινε οδηγίες στους εργάτες που κουβαλούσαν τα πράγματα της μετακόμισης, αναζητούσε το κουτί για το σαλόνι και την είδε μέσα σε μια σακούλα σκουπιδιών.

c. Όταν ο Λάμπρος έδινε οδηγίες στους εργάτες που κουβαλούσαν τα πράγματα της μετακόμισης, αναζητούσε το κουτί για την πιλοτή και το είδε μέσα σε μια σακούλα σκουπιδιών.

d. Όταν ο Λάμπρος έδινε οδηγίες στους εργάτες που κουβαλούσαν τα πράγματα της μετακόμισης, αναζητούσε το κουτί για την πιλοτή και την είδε μέσα σε μια σακούλα σκουπιδιών.

“When Lambros instructed the workers who were carrying the things of the move, he was looking for the box for the living room/pilotis and he saw it in a garbage bag.”

Item 24a

a. Ο μεγάλος ποιητής που αγαπούσε πολύ τον τόπο του, έψαχνε το ποίημα για το νησί και το βρήκε μέσα σε ένα τετράδιο ποιημάτων.

b. Ο μεγάλος ποιητής που αγαπούσε πολύ τον τόπο του, έψαχνε το ποίημα για το νησί και τη βρήκε μέσα σε ένα τετράδιο ποιημάτων.

c. Ο μεγάλος ποιητής που αγαπούσε πολύ τον τόπο του, έψαχνε το ποίημα για την πόλη και το βρήκε μέσα σε ένα τετράδιο ποιημάτων.

d. Ο μεγάλος ποιητής που αγαπούσε πολύ τον τόπο του, έψαχνε το ποίημα για την πόλη και τη βρήκε μέσα σε ένα τετράδιο ποιημάτων.

“The great poet who loved his place, was looking for the poem about the island/city and he found it in a poem notebook.

Feminine heads

Item 1b

a. Ο Αντώνης που συμμάζευε το σπίτι, γύρευε τη μοκέτα για την είσοδο και τη βρήκε πάνω στο τραπέζι της κουζίνας.

b. Ο Αντώνης που συμμάζευε το σπίτι, γύρευε τη μοκέτα για την είσοδο και το βρήκε πάνω στο τραπέζι της κουζίνας.

c. Ο Αντώνης που συμμάζευε το σπίτι, γύρευε τη μοκέτα για το ισόγειο και τη βρήκε πάνω στο τραπέζι της κουζίνας.

d. Ο Αντώνης που συμμάζευε το σπίτι, γύρευε τη μοκέτα για το ισόγειο και το βρήκε πάνω στο τραπέζι της κουζίνας.

“Antonis, who was clearing the house up, was looking for the carpet for the entrance/ground floor and he found it on the kitchen table.”

Item 2b

a. Όταν ο Αλέξανδρος επέστρεψε από το σούπερ-μάρκετ, αναζητούσε την ζύμη για την πίτσα και την είδε μέσα στη σακούλα με τα ψώνια.

b. Όταν ο Αλέξανδρος επέστρεψε από το σούπερ-μάρκετ, αναζητούσε την ζύμη για την πίτσα και το είδε μέσα στη σακούλα με τα ψώνια.

c. Όταν ο Αλέξανδρος επέστρεψε από το σούπερ-μάρκετ, αναζητούσε την ζύμη για το ψωμί και την είδε μέσα στη σακούλα με τα ψώνια.

d. Όταν ο Αλέξανδρος επέστρεψε από το σούπερ-μάρκετ, αναζητούσε την ζύμη για το ψωμί και το είδε μέσα στη σακούλα με τα ψώνια.

“When Alexander returned from the supermarket, he was looking for the dough for the pizza/bread and he saw it into the shopping bag.”

Item 3b

a. Ο διάσημος δικηγόρος της πολυεθνικής που γιόρταζε είκοσι χρόνια γάμου έψαχνε την τούρτα για την επέτειο και την ανακάλυψε μέσα σε φύλλα αλουμινόχαρτου.

b. Ο διάσημος δικηγόρος της πολυεθνικής που γιόρταζε είκοσι χρόνια γάμου έψαχνε την τούρτα για την επέτειο και το ανακάλυψε μέσα σε φύλλα αλουμινόχαρτου.

c. Ο διάσημος δικηγόρος της πολυεθνικής που γιόρταζε είκοσι χρόνια γάμου έψαχνε την τούρτα για το γραφείο και την ανακάλυψε μέσα σε φύλλα αλουμινόχαρτου.

d. Ο διάσημος δικηγόρος της πολυεθνικής που γιόρταζε είκοσι χρόνια γάμου έψαχνε την τούρτα για το γραφείο και το ανακάλυψε μέσα σε φύλλα αλουμινόχαρτου.

“The famous lawyer of the multinational company who celebrated twenty years of marriage was looking for the cake for the anniversary/office and he discovered it in aluminium foil sheets.”

Item 4b

a. Ο γραμματέας του υπουργού που έφτασε νωρίτερα από τους καλεσμένους γύρευε την αφίσα για τη γιορτή και την είδε μέσα σε μια σακούλα σκουπιδιών.

b. Ο γραμματέας του υπουργού που έφτασε νωρίτερα από τους καλεσμένους γύρευε την αφίσα για τη γιορτή και το είδε μέσα σε μια σακούλα σκουπιδιών.

c. Ο γραμματέας του υπουργού που έφτασε νωρίτερα από τους καλεσμένους γύρευε την αφίσα για το πάρτι και την είδε μέσα σε μια σακούλα σκουπιδιών.

d. Ο γραμματέας του υπουργού που έφτασε νωρίτερα από τους καλεσμένους γύρευε την αφίσα για το πάρτι και το είδε μέσα σε μια σακούλα σκουπιδιών.

“The secretary of the minister, who arrived earlier than the guests, was looking for the poster for the feast/party and he saw it in a garbage bag.”

Item 5b

a. Όταν ο φύλακας τελείωσε τη βάρδια στην κήπο, αναζητούσε την πόρτα για την έξοδο και την εντόπισε δίπλα στο ασανσέρ του προσωπικού.

b. Όταν ο φύλακας τελείωσε τη βάρδια στην κήπο, αναζητούσε την πόρτα για την έξοδο και το εντόπισε δίπλα στο ασανσέρ του προσωπικού.

c. Όταν ο φύλακας τελείωσε τη βάρδια στην κήπο, αναζητούσε την πόρτα για το κτίριο και την εντόπισε δίπλα στο ασανσέρ του προσωπικού.

d. Όταν ο φύλακας τελείωσε τη βάρδια στην κήπο, αναζητούσε την πόρτα για το κτίριο και το εντόπισε δίπλα στο ασανσέρ του προσωπικού.

“When the guard finished the shift in the garden, he was looking for the door for the exit/building and he found it next to the elevator.”

Item 6b

a. Καθώς ο υπάλληλος τακτοποιούσε τα πράγματα στο μαγαζί, έψαχνε την ομπρέλα για τη βιτρίνα και την ανακάλυψε πάνω στο γραφείο του αφεντικού του.

b. Καθώς ο υπάλληλος τακτοποιούσε τα πράγματα στο μαγαζί, έψαχνε την ομπρέλα για τη βιτρίνα και το ανακάλυψε πάνω στο γραφείο του αφεντικού του.

c. Καθώς ο υπάλληλος τακτοποιούσε τα πράγματα στο μαγαζί, έψαχνε την ομπρέλα για το μπαλκόνι και την ανακάλυψε πάνω στο γραφείο του αφεντικού του.

d. Καθώς ο υπάλληλος τακτοποιούσε τα πράγματα στο μαγαζί, έψαχνε την ομπρέλα για το μπαλκόνι και το ανακάλυψε πάνω στο γραφείο του αφεντικού του.

“While the clerk was clearing the shop up, he was looking for the umbrella for the showcase/balcony and he discovered it on his boss's desk.”

Item 7b

a. Ο ζωγράφος που αγαπούσε τον τόπο του και ταξίδευε σε ασυνήθιστους προορισμούς, γύρευε τη διαδρομή για την έρημο και τη βρήκε μέσα στο μπλοκ ζωγραφικής του.

b. Ο ζωγράφος που αγαπούσε τον τόπο του και ταξίδευε σε ασυνήθιστους προορισμούς, γύρευε τη διαδρομή για την έρημο και το βρήκε μέσα στο μπλοκ ζωγραφικής του.

c. Ο ζωγράφος που αγαπούσε τον τόπο του και ταξίδευε σε ασυνήθιστους προορισμούς, γύρευε τη διαδρομή για το γήπεδο και τη βρήκε μέσα στο μπλοκ ζωγραφικής του.

d. Ο ζωγράφος που αγαπούσε τον τόπο του και ταξίδευε σε ασυνήθιστους προορισμούς, γύρευε τη διαδρομή για το γήπεδο και το βρήκε μέσα στο μπλοκ ζωγραφικής του.

“The painter who loved his place and traveled to unusual destinations, was looking for the route to the desert/stadium and he found it in his painting block.”

Item 8b

a. Ο συμπαθητικός τουρίστας που είχε χαθεί ψάχνοντας για αξιοθέατα, αναζητούσε την ταμπέλα για τη λεωφόρο και την είδε πάνω στη γέφυρα των τρένων.

b. Ο συμπαθητικός τουρίστας που είχε χαθεί ψάχνοντας για αξιοθέατα, αναζητούσε την ταμπέλα για τη λεωφόρο και το είδε πάνω στη γέφυρα των τρένων.

c. Ο συμπαθητικός τουρίστας που είχε χαθεί ψάχνοντας για αξιοθέατα, αναζητούσε την ταμπέλα για το μουσείο και την είδε πάνω στη γέφυρα των τρένων.

d. Ο συμπαθητικός τουρίστας που είχε χαθεί ψάχνοντας για αξιοθέατα, αναζητούσε την ταμπέλα για το μουσείο και το είδε πάνω στη γέφυρα των τρένων.

“The sympathetic tourist, who was lost looking for sightseeing, was looking for the sign for the avenue/museum and he saw it on the train bridge.”

Item 9b

a. Όταν ο Αντρέας συμμάζευε το εξοχικό του, έψαχνε την πετσέτα για την άμμο και την εντόπισε δίπλα στο κουτί του απορρυπαντικού.

b. Όταν ο Αντρέας συμμάζευε το εξοχικό του, έψαχνε την πετσέτα για την άμμο και το εντόπισε δίπλα στο κουτί του απορρυπαντικού.

c. Όταν ο Αντρέας συμμάζευε το εξοχικό του, έψαχνε την πετσέτα για το μπάνιο και την εντόπισε δίπλα στο κουτί του απορρυπαντικού.

d. Όταν ο Αντρέας συμμάζευε το εξοχικό του, έψαχνε την πετσέτα για το μπάνιο και το εντόπισε δίπλα στο κουτί του απορρυπαντικού.

“When Andreas was clearing his cottage up, he was looking for the towel for the bathroom/sand and he found it next to the detergent box.”

Item 10b

a. Ο θυρωρός που είχε διαβάσει το δελτίο καιρού με τα ακραία φαινόμενα, αναζητούσε την ανακοίνωση για τη σκόνη και την ανακάλυψε πάνω στον πίνακα ανακοινώσεων της πολυκατοικίας.

b. Ο θυρωρός που είχε διαβάσει το δελτίο καιρού με τα ακραία φαινόμενα, αναζητούσε την ανακοίνωση για τη σκόνη και το ανακάλυψε πάνω στον πίνακα ανακοινώσεων της πολυκατοικίας.

c. Ο θυρωρός που είχε διαβάσει το δελτίο καιρού με τα ακραία φαινόμενα, αναζητούσε την ανακοίνωση για το χιόνι και την ανακάλυψε πάνω στον πίνακα ανακοινώσεων της πολυκατοικίας.

d. Ο θυρωρός που είχε διαβάσει το δελτίο καιρού με τα ακραία φαινόμενα, αναζητούσε την ανακοίνωση για το χιόνι και το ανακάλυψε πάνω στον πίνακα ανακοινώσεων της πολυκατοικίας.

“The concierge, who had read the weather report with the extreme phenomena, was looking for the report for the dust/snow and he found it on the panel of the block of flats.”

Item 11b

a. Όταν ο υπομονετικός ράφτης επέστρεψε στη δουλειά του γύρευε την κλωστή για την κουρτίνα και την ανακάλυψε δίπλα στο κουτί με τα ραφτικά.

b. Όταν ο υπομονετικός ράφτης επέστρεψε στη δουλειά του γύρευε την κλωστή για την κουρτίνα και το ανακάλυψε δίπλα στο κουτί με τα ραφτικά.

c. Όταν ο υπομονετικός ράφτης επέστρεψε στη δουλειά του γύρευε, την κλωστή για το φόρεμα και την ανακάλυψε δίπλα στο κουτί με τα ραφτικά.

d. Όταν ο υπομονετικός ράφτης επέστρεψε στη δουλειά του γύρευε την κλωστή για το φόρεμα και το ανακάλυψε δίπλα στο κουτί με τα ραφτικά.

“When the patient tailor returned to work, he was looking for the thread for the curtain/dress and he discovered it next to the sewing box.”

Item 12b

a. Όταν ο διάσημος ενδυματολόγος επέστρεψε στο μαγαζί του, έψαχνε την απόδειξη για τη γραβάτα και τη βρήκε πάνω στο γραφείο του γραμματέα του.

b. Όταν ο διάσημος ενδυματολόγος επέστρεψε στο μαγαζί του, έψαχνε την απόδειξη για τη γραβάτα και το βρήκε πάνω στο γραφείο του γραμματέα του.

c. Όταν ο διάσημος ενδυματολόγος επέστρεψε στο μαγαζί του, έψαχνε την απόδειξη για το φόρεμα και τη βρήκε πάνω στο γραφείο του γραμματέα του.

d. Όταν ο διάσημος ενδυματολόγος επέστρεψε στο μαγαζί του, έψαχνε την απόδειξη για το φόρεμα και το βρήκε πάνω στο γραφείο του γραμματέα του.

“When the famous stylist returned to his shop, he was looking for the recipe for the tie/dress and he found it on his secretary's desk.”

Item 13b

a. Ο Παύλος που δεν πρόλαβε να συγυρίσει το σπίτι, γύρευε τη στολή για τη γιορτή και την είδε δίπλα στο κουστούμι του γάμου του.

b. Ο Παύλος που δεν πρόλαβε να συγυρίσει το σπίτι, γύρευε τη στολή για τη γιορτή και το είδε δίπλα στο κουστούμι του γάμου του.

c. Ο Παύλος που δεν πρόλαβε να συγυρίσει το σπίτι, γύρευε τη στολή για το χιόνι και την είδε δίπλα στο κουστούμι του γάμου του.

d. Ο Παύλος που δεν πρόλαβε να συγυρίσει το σπίτι, γύρευε τη στολή για το χιόνι και το είδε δίπλα στο κουστούμι του γάμου του.

“Paul, who failed to confront the house, was looking for the outfit for the feast/snow and he found it next to his wedding suit.”

Item 14b

a. Ο διάσημος ιστορικός που ήταν πολλά χρόνια παντρεμένος, αναζητούσε την πρόσκληση για την επέτειο και την ανακάλυψε δίπλα στο άδειο μπουκάλι ουίσκι.

b. Ο διάσημος ιστορικός που ήταν πολλά χρόνια παντρεμένος, αναζητούσε την πρόσκληση για την επέτειο και το ανακάλυψε δίπλα στο άδειο μπουκάλι ουίσκι.

c. Ο διάσημος ιστορικός που ήταν πολλά χρόνια παντρεμένος, αναζητούσε την πρόσκληση για το μουσείο και την ανακάλυψε δίπλα στο άδειο μπουκάλι ουίσκι.

d. Ο διάσημος ιστορικός που ήταν πολλά χρόνια παντρεμένος, αναζητούσε την πρόσκληση για το μουσείο και το ανακάλυψε δίπλα στο άδειο μπουκάλι ουίσκι.

“The famous historian, who was married for many years, was looking for the invitation to the museum/anniversary and he discovered it next to the empty whiskey bottle.”

Item 15b

a. Όταν ο Αλέξανδρος επέστρεψε από την αγορά, έψαχνε τη σκούπα για την άμμο και την εντόπισε πάνω στο τραπέζι του σαλονιού.

b. Όταν ο Αλέξανδρος επέστρεψε από την αγορά, έψαχνε τη σκούπα για την άμμο και το εντόπισε πάνω στο τραπέζι του σαλονιού.

c. Όταν ο Αλέξανδρος επέστρεψε από την αγορά, έψαχνε τη σκούπα για το μπάνιο και την εντόπισε πάνω στο τραπέζι του σαλονιού.

d. Όταν ο Αλέξανδρος επέστρεψε από την αγορά, έψαχνε τη σκούπα για το μπάνιο και το εντόπισε πάνω στο τραπέζι του σαλονιού.

“When Alexander returned from the market, he was looking for the broom for the sand/bathroom and he found it on his coffee table.”

Item 16b

a. Ο υπάλληλος που παρέλαβε τη μυστική παραγγελία, γύρευε την καρφίτσα για την κουρτίνα και την ανακάλυψε μέσα σε μια κούτα με ρούχα.

b. Ο υπάλληλος που παρέλαβε τη μυστική παραγγελία, γύρευε την καρφίτσα για την κουρτίνα και το ανακάλυψε μέσα σε μια κούτα με ρούχα.

c. Ο υπάλληλος που παρέλαβε τη μυστική παραγγελία, γύρευε την καρφίτσα για το φουστάνι και την ανακάλυψε μέσα σε μια κούτα με ρούχα.

d. Ο υπάλληλος που παρέλαβε τη μυστική παραγγελία, γύρευε την καρφίτσα για το φουστάνι και το ανακάλυψε μέσα σε μια κούτα με ρούχα.

“The employee who received the secret order was looking for the pin for the curtain/dress and he discovered it in a box with clothes.”

Item 17b

a. Καθώς ο Γιάννης έφευγε από το παλιό σπίτι, αναζητούσε τη σκάλα για την έξοδο και την εντόπισε δίπλα στον πίνακα του διάσημου ζωγράφου.

b. Καθώς ο Γιάννης έφευγε από το παλιό σπίτι, αναζητούσε τη σκάλα για την έξοδο και το εντόπισε δίπλα στον πίνακα του διάσημου ζωγράφου.

c. Καθώς ο Γιάννης έφευγε από το παλιό σπίτι, αναζητούσε τη σκάλα για το ισόγειο και την εντόπισε δίπλα στον πίνακα του διάσημου ζωγράφου.

d. Καθώς ο Γιάννης έφευγε από το παλιό σπίτι, αναζητούσε τη σκάλα για το ισόγειο και το εντόπισε δίπλα στον πίνακα του διάσημου ζωγράφου.

“While Giannis was leaving the old house, he was looking for the stairway for the exit/ground floor and he found it next to the famous painter's painting.”

Item 18b

a. Ο απρόσεκτος μάγειρας που έχανε τα πράγματά του συνεχώς έψαχνε τη συνταγή για την πίτσα και τη βρήκε πάνω στο ράφι της κουζίνας.

b. Ο απρόσεκτος μάγειρας που έχανε τα πράγματά του συνεχώς έψαχνε τη συνταγή για την πίτσα και το βρήκε πάνω στο ράφι της κουζίνας.

c. Ο απρόσεκτος μάγειρας που έχανε τα πράγματά του συνεχώς έψαχνε τη συνταγή για το ψωμί και τη βρήκε πάνω στο ράφι της κουζίνας.

d. Ο απρόσεκτος μάγειρας που έχανε τα πράγματά του συνεχώς έψαχνε τη συνταγή για το ψωμί και το βρήκε πάνω στο ράφι της κουζίνας.

“The careless cook who lost his stuff constantly was looking for the recipe for the pizza/bread and he found it on the kitchen shelf.”

Item 19b

a. Καθώς ο διακοσμητής έψαχνε τα κατάλληλα αντικείμενα για τη διακόσμηση του σπιτιού, γύρευε τη γλάστρα για τη βιτρίνα και τηνεντόπισε πάνω στο ράφι της βιβλιοθήκης.

b. Καθώς ο διακοσμητής έψαχνε τα κατάλληλα αντικείμενα για τη διακόσμηση του σπιτιού, γύρευε τη γλάστρα για τη βιτρίνα και το εντόπισε πάνω στο ράφι της βιβλιοθήκης.

c. Καθώς ο διακοσμητής έψαχνε τα κατάλληλα αντικείμενα για τη διακόσμηση του σπιτιού, γύρευε τη γλάστρα για το μπαλκόνι και την εντόπισε πάνω στο ράφι της βιβλιοθήκης.

d. Καθώς ο διακοσμητής έψαχνε τα κατάλληλα αντικείμενα για τη διακόσμηση του σπιτιού, γύρευε τη γλάστρα για το μπαλκόνι και το εντόπισε πάνω στο ράφι της βιβλιοθήκης.

“As the decorator was looking for the appropriate items for decorating the house, he was looking for the flower pot for the showcase/balcony nailed on the library shelf.”

Item 20b

a. Ο ακούραστος τουρίστας που ήθελε να επισκεφτεί διάφορους τόπους και αξιοθέατα, αναζητούσε την πινακίδα για την έρημο και την ανακάλυψε μέσα στην πυκνή βλάστηση της περιοχής.

b. Ο ακούραστος τουρίστας που ήθελε να επισκεφτεί διάφορους τόπους και αξιοθέατα, αναζητούσε την πινακίδα για την έρημο και το ανακάλυψε μέσα στην πυκνή βλάστηση της περιοχής.

c. Ο ακούραστος τουρίστας που ήθελε να επισκεφτεί διάφορους τόπους και αξιοθέατα, αναζητούσε την πινακίδα για το κτίριο και την ανακάλυψε μέσα στην πυκνή βλάστηση της περιοχής.

d. Ο ακούραστος τουρίστας που ήθελε να επισκεφτεί διάφορους τόπους και αξιοθέατα, αναζητούσε την πινακίδα για το κτίριο και το ανακάλυψε μέσα στην πυκνή βλάστηση της περιοχής.

“The tireless tourist, who wanted to visit various places and sights, was looking for the sign for the desert/building and he discovered it in the dense vegetation of the area.”

Item 21b

a. Λόγω των έργων που γίνονται στην περιοχή ο Κώστας έψαχνε την κατεύθυνση για τη λεωφόρο και το βρήκε δίπλα στους γερανούς της κοινότητας.

b. Λόγω των έργων που γίνονται στην περιοχή ο Κώστας έψαχνε την κατεύθυνση για τη λεωφόρο και τη βρήκε δίπλα στους γερανούς της κοινότητας.

c. Λόγω των έργων που γίνονται στην περιοχή ο Κώστας έψαχνε την κατεύθυνση για το γραφείο και το βρήκε δίπλα στους γερανούς της κοινότητας.

d. Λόγω των έργων που γίνονται στην περιοχή ο Κώστας έψαχνε την κατεύθυνση για το γραφείο και τη βρήκε δίπλα στους γερανούς της κοινότητας.

“Due to the works made in the area, Kostas was looking for the direction for the avenue/office and he found it next to the tow trucks of the township.”

Item 22b

a. Ο νεαρός που έπαιζε στις λάσπες με τους φίλους του γύρευε την κάρτα για την είσοδο και την ανακάλυψε μέσα στο μπουφάν του.

b. Ο νεαρός που έπαιζε στις λάσπες με τους φίλους του γύρευε την κάρτα για την είσοδο και το ανακάλυψε μέσα στο μπουφάν του.

c. Ο νεαρός που έπαιζε στις λάσπες με τους φίλους του γύρευε την κάρτα για το γήπεδο και την ανακάλυψε μέσα στο μπουφάν του.

d. Ο νεαρός που έπαιζε στις λάσπες με τους φίλους του γύρευε την κάρτα για το γήπεδο και το ανακάλυψε μέσα στο μπουφάν του.

“The young man who played in the mud with his friends was looking for the card for the entrance/pitch and he discovered it in his jacket.”

Item 23b

a. Ο διάσημος σχεδιαστής μόδας που επέλεγε ασυνήθιστους συνδυασμούς ρούχων, αναζητούσε κορδέλα για τη γραβάτα και την είδε δίπλα στο κουτί με τις βελόνες.

b. Ο διάσημος σχεδιαστής μόδας που επέλεγε ασυνήθιστους συνδυασμούς ρούχων, αναζητούσε κορδέλα για τη γραβάτα και το είδε δίπλα στο κουτί με τις βελόνες.

c. Ο διάσημος σχεδιαστής μόδας που επέλεγε ασυνήθιστους συνδυασμούς ρούχων, αναζητούσε κορδέλα για το φουστάνι και την είδε δίπλα στο κουτί με τις βελόνες.

d. Ο διάσημος σχεδιαστής μόδας που επέλεγε ασυνήθιστους συνδυασμούς ρούχων, αναζητούσε κορδέλα για το φουστάνι και το είδε δίπλα στο κουτί με τις βελόνες.

“The famous fashion designer who was choosing unusual combinations of clothes, was looking for the ribbon for the tie/dress and he saw it next to the needle box.”

Item 24b

a. Ο Πέτρος που ετοιμαζόταν να βγει έξω, έψαχνε τη μάσκα για τη σκόνη και την εντόπισε μέσα στο μπουφάν που φορούσε.

b. Ο Πέτρος που ετοιμαζόταν να βγει έξω, έψαχνε τη μάσκα για τη σκόνη και την εντόπισε μέσα στο μπουφάν που φορούσε.

c. Ο Πέτρος που ετοιμαζόταν να βγει έξω, έψαχνε τη μάσκα για το χιόνι και την εντόπισε μέσα στο μπουφάν που φορούσε.

d. Ο Πέτρος που ετοιμαζόταν να βγει έξω, έψαχνε τη μάσκα για το χιόνι και την εντόπισε μέσα στο μπουφάν που φορούσε.

“Peter, who was about to get out, was looking for the the mask for the dust/snow and he found it in the jacket he was wearing.”
